# Novel library synthesis of 3,4-disubstituted pyridin-2(1*H*)-ones via cleavage of pyridine-2-oxy-7-azabenzotriazole ethers under ionic hydrogenation conditions at room temperature

**DOI:** 10.3762/bjoc.17.16

**Published:** 2021-01-18

**Authors:** Romain Pierre, Anne Brethon, Sylvain A Jacques, Aurélie Blond, Sandrine Chambon, Sandrine Talano, Catherine Raffin, Branislav Musicki, Claire Bouix-Peter, Loic Tomas, Gilles Ouvry, Rémy Morgentin, Laurent F Hennequin, Craig S Harris

**Affiliations:** 1 Galderma SA, Rue d'Entre-deux-Villes 10, 1814 Vevey, Switzerland; 2 Edelris, 60, Avenue Rockefeller, Bioparc, Bioserra 1 Building, 69008 Lyon, France

**Keywords:** 7-azabenzotriazole, hinge-binder, ionic hydrogenation, library, pyridine-2(1*H*)-one

## Abstract

In our hands, efficient access to the 4-amino-3-carboxamide disubstituted pyridine-2(1*H*)-one kinase hinge-binder motif proved to be more challenging than anticipated requiring a significant investment in route scouting and optimization. This full paper focuses on the synthesis issues that we encountered during our route exploration and the original solutions we found that helped us to identify two optimized library-style processes to prepare our large kinase inhibitor library.

## Introduction

During a recent medicinal chemistry program targeting a kinase to treat skin disorders, we identified the 4-amino-3-carboxamide disubstituted pyridine-2(1*H*)-one motif (**1**) as an interesting starting point. Recently, both Roche and Genentech researchers have described the first route to this scaffold for their FAK and EGFR mutant programs, respectively [1–2]. Both processes started from the versatile 4-iodo-2-methoxynicotinaldehyde (**3**). Oxidation to the carboxylic acid followed by chlorodehydration afforded the key library building block acid chloride **2**. The libraries were prepared in a 3-step manner: 1) amide coupling; 2) deprotection of the 2-methoxypyridine through hydrolysis at elevated temperatures; and 3) the final S_N_Ar or Ullman step to introduce the amine vector with variable yields and chromatographic purification in between steps.

**Figure 1:**
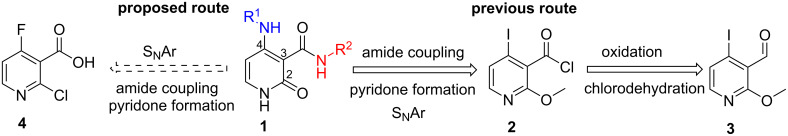
Retrosynthetic disconnection of our privileged kinase scaffold **1**.

As we planned to fix substitution at C-4 to a *cis*-diaminocyclohexane fragment and focus our main exploration from the amide vector at C-3, we envisaged it would be more logical to exploit the cheaper and more readily-available precursor, 2-chloro-4-fluoronicotinic acid (**4**) with a goal of creating a rapid, 3-step route requiring a single preparative LC–MS purification at the end of the sequence (Figure 1).

## Results and Discussion

### Exploration of the C-3 amide vector: formation of the pyridine-2-(1*H*)-one motif by palladium catalysis

**Scheme 1:**
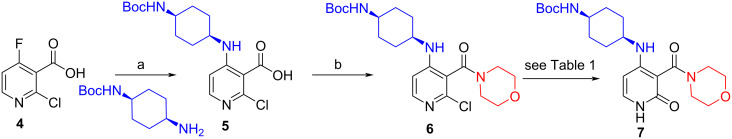
Reagents and conditions: (a) MeOH, DIPEA, reflux, 70%; b) TBTU, DIPEA, DMF, rt, 91%.

We decided to validate the route by preparing morpholine amide **7**. The synthesis started by selective S_N_Ar reaction with **4** and *tert*-butyl ((*cis*)-4-aminocyclohexyl)carbamate affording the intermediate nicotinic acid **5** in 70% yield without the need for chromatography. Subsequent amide coupling using TBTU afforded 2-chloro precursor **6** in excellent yield (Scheme 1).

We anticipated that the transformation of 2-chloropyridine precursor **6** to the final pyridine-2-(1*H*)-one **7** would provide the biggest challenge, especially after introduction of the strong donor cyclohexylamine moiety at C-4 deactivating the pyridine ring towards nucleophilic attack (Table 1). No product was observed via direct S_N_Ar using KOH (Table 1, entry 1) [3]. Acidic conditions (Table 1, entries 2–5) [4], where we can expect protonation thus activation of the pyridine ring towards nucleophilic attack, resulted in only traces of product along with hydrolysis of the amide moiety at C-3. Finally, we turned out attention to transition metal-catalyzed formation of phenols from aryl halides [5]. After another round of screening, we successfully applied palladium-catalyzed conditions discovered by the Buchwald group [6], using KOH as the nucleophile and X-Phos as the ligand, to afford **7** in 83% isolated yield (Table 1, entry 6).

**Table 1:** Selected results from conditions’ screening for pyridin-2-(1*H*)-one formation (**7**).

Entry	Conditions	% Conversion by UV-LC–MS	Isolated yield (%)

1	KOH (5 equiv), DMSO, 100 °C, 1 h	degradation	–
2	HCl in iPrOH, H_2_O, 50–100 °C	trace	–
3	c. HCl_aq_, 1,4-dioxane, 80–120 °C	trace	–
4	NH_4_OAc, AcOH, 120 °C, 3 h	trace	–
5	6 N HCl_aq_, 130 °C, 2 h	trace	–
6	Pd_2_(dba)_3_, X-Phos, KOH, dioxane, 100 °C, 4 h	92	83

**Scheme 2:**
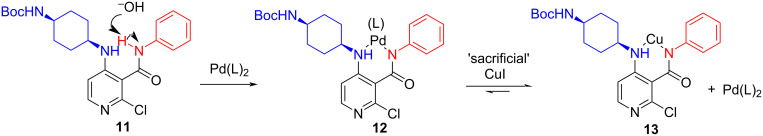
Proposed mechanistic explanation for the liberation of the Pd catalytic cycle after addition of sacrificial Cu(I).

With these conditions in hand, we prepared a small library with different amides at C-3 to test the robustness of this new process before going into library production (Table 2). However, to our surprise, the process worked only for compounds with aliphatic amides at C-3 affording only acceptable yields of pyridine intermediate (**9a–c**) with the major byproduct arising from the dechlorination of **8**. Application of the same conditions with aromatic amides at C-3 (**8d**,**e**) failed with only trace quantities of final product observed. We speculated that the aromatic carboxamide NH was fully deprotonated during the reaction and the amidic anion trapped the Pd catalyst irreversibly affording **12**, thus halting the catalytic cycle (Scheme 2). We overcame this issue by adding 0.2 equiv of Cu(I), reported as a strong binder to the *ortho*-amino aromatic carboxamides [7], which served as a sacrificial metal cation, preventing Pd ligation and permitting the reaction to turnover resulting in albeit poor isolated yields of **9d,e** (Table 2). All attempts to carry out the reaction using only CuI failed, confirming that Pd was necessary to catalyze this transformation. It was preferential to purify the Boc protected intermediates **9a–e** by Prep-LC–MS as the final products **10a–e** were difficult to purify owing to their particularly poor solubility in the mobile phase. The final products were delivered as their HCl salts following Boc deprotection using HCl in dioxane overnight.

**Table 2:** Validation of library conditions.^a^

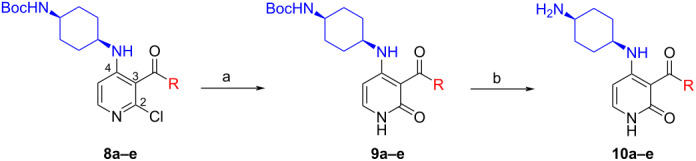			

Compound	Amide (R)	% Conversion of step a by UV-LC–MS	Isolated yield (%)

**10a**	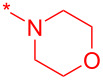	95	80
**10b**	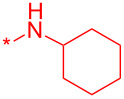	95	44
**10c**	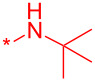	100	66
**10d**^b^	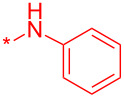	100	37 (0)^c^
**10e**^b^	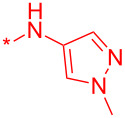	100	19

^a^Reagents and condtions: (a) Pd_2_(dba)_3_, X-Phos, KOH, dioxane/water, 100 °C, 12 h; b) 4 N HCl in dioxane, 12 h. ^b^CuI (0.2 equiv) was added; ^c^same reaction in the absence of Pd_2_dba_3_ and X-phos.

### Exploration of the C-3 amide vector: formation of the pyridine-2(1*H*)-one motif by ionic hydrogenative cleavage of C-2-OAt ether

**Scheme 3:**
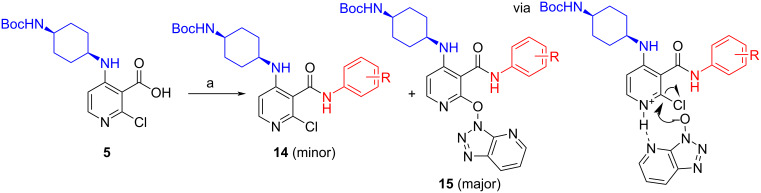
Formation of C2–OAt ether **15** using HATU. Reagents and condtions: (a) HATU, DIPEA, DCM, rt, 16 h, ((**14**) 5%, (**15**) 95% conversion by UV-LC–MS), 58% isolated yield (**15**).

Encouraged by our results, we started the library production planning to use the 3-step process requiring 2 purification steps by preparative LC–MS. However, as we planned to introduce a significant amount of steric and electronic diversity from the C-3 amide vector, we switched the amide coupling agent from TBTU to the more reactive HATU [8–9]. To our surprise, the amide coupling step furnished almost quantitatively the C2–OAt ether **15** [10] and only traces of the expected C2–Cl amide product **14**. Closer inspection of the reaction progression by UV-LC–MS revealed the expected product **14** is formed first and is slowly transformed to **15** overnight. We postulated that the acidic HOAt (p*K*_a_ = 3.76) liberated from the coupling reaction must be protonating thus activating the basic pyridine ring toward S_N_Ar with the ^−^OAt anion in an intermolecular or intramolecular fashion (Scheme 3). We saw this observation as having the potential to solve our hydrolysis issues and decided to explore it further.

Inspection of the literature revealed that activated ethers (e.g., OBt) have been utilized in synthetic strategies as masked leaving groups notably on azines and displaced using an excess of nucleophile under high temperatures. More relevantly, among the few references, hydrolysis of the (het)aryl–OBt bond was most documented in a refluxing mixture of AcOH/H_2_O [11]. We anticipated that under these acidic conditions, hydrolysis of our py–OAt ether **15** would be accompanied by in situ deprotection of the Boc group to afford directly our final pyridin-2-(1*H*)-one products **16** and thereby eliminating a purification stage compared to the previous route. As literature was scarce for this transformation and we were also concerned about hydrolysis of the amide bond under aqueous acidic conditions, we decided to carry out a final reaction conditions’ screening using **15** before starting the library production (Table 3).

**Table 3:** Selected results from conditions screening to form pyridin-2-(1*H*)-one **16**.

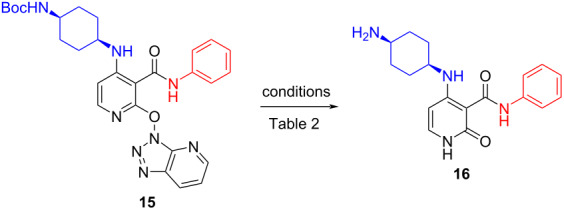			

Entry	Conditions	% Conversion by UV-LC–MS	Isolated yield (%)

1	AcOH/H_2_O (4:1), 140 °C, 1 h	65	45
2	2 N HCl_aq_, MeOH, 100 °C	15	–
3	Zn_solid_, AcOH/H_2_O, 70 °C, 5 h	30	–
4	TFA, TES, DCM, rt, 10 d	76	–
5	TFA, TES, 1,2-DCE, 50 °C, 24 h	57	N/I
6	TFA/TES (4:1), rt, 24 h	62	45
7	TFA/TES/H_2_O (4:1:1), rt, 24 h	69	61
8^a^	neat TFA	–	–
9	TFA/H_2_O (4:1), 96 h	12	N/I

^a^Major product corresponded to loss of Boc group but retention of C2–OAt ether; N/I = not isolated; TES = triethylsilane:

As attested by the results presented in Table 3, the Py–OAt ether was much easier to hydrolyze than the Py–Cl bond (Table 1). Using conditions described in the literature (Table 3, entry 1), we obtained a 45% isolated yield of **16** with side products arising namely from acetylation of the product that were easily removed by preparative LC–MS. Using aqueous HCl led to severe degradation with the dominant impurity coming from cleavage of the amide bond (Table 3, entry 2) and dissolving metal reduction conditions using zinc were quickly excluded as just 30% conversion was achieved with the added work-up complications (Table 3, entry 3). Finally and rather fortuitously, we turned our attention to ionic hydrogenation conditions [12]. Although ionic hydrogenation conditions have never been cited for this type of transformation, we anticipated that, if successful, sample preparation would be further simplified for the final preparative LC–MS purification step owing to the high volatility of the reaction medium compared to AcOH/H_2_O mixtures (Table 3, entry 1). To our delight, our first attempt using CH_2_Cl_2_ as the solvent at rt afforded a very slow but clean conversion to **16** after 10 d at rt (Table 3, entry 4). The reaction time was reduced to 24 h by heating the reaction at 50 °C (Table 3, entry 5).

**Scheme 4:**
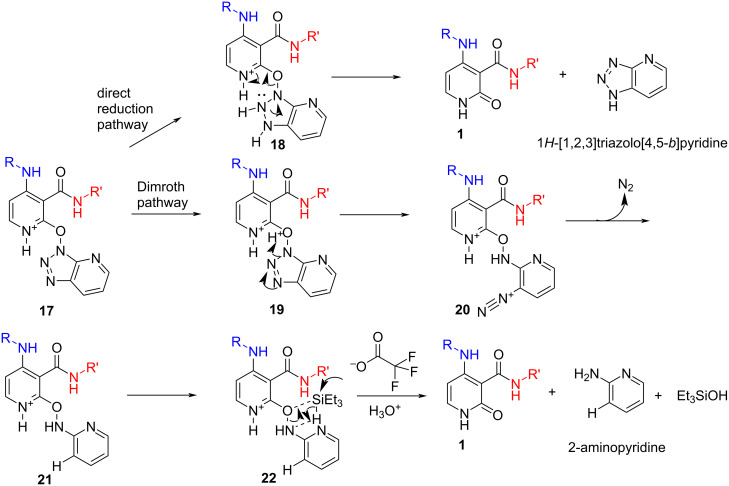
Proposed mechanistic pathways for the transformation of Py–OAt ethers **17** to the pyridin-2*H*-one **1** motif.

To decrease reaction times further, we carried out the reaction in neat TFA/TES mixture (4:1 v/v, 5 volumes) and obtained a 62% conversion at just rt after 24 h (Table 3, entry 6). The product distribution was even further improved by addition of water to the reaction mixture to avoid a trifluoroacetamide impurity, presumably forming by reaction with the cyclohexylamine moiety. In the absence of triethylsilane (TES), only deprotection of the Boc group was observed when using TFA (Table 3, entry 8) and when the reaction was carried out in mixture of TFA/H_2_O (4:1) over 4 days, only 12% of desired pyridine-2-(1*H*)-one **22** was observed (Scheme 4).

Despite literature analogies being scarce in this area [13–14], we propose that the reaction could occur through 2 possible pathways. The first proposition concerns the direct reduction of the N(3)=N(2) double bond of the 7-azabenzotriazole ring to the 7-azabenzotriazoline ring **18** and cleavage of the N–O bond through delocalization of the lone pair at N-2 to liberate **1** and 1*H*-[1,2,3]triazolo[4,5-*b*]pyridine as the byproduct. The second pathway occurs through 4 key steps: a) activation of the pyridine in the acidic media; b) ring-opening of the triazole moiety through a Dimroth rearrangement process affording **20** (reaction becomes instantly bright red); c) reduction of diazonium species to afford intermediate **21**, observed by UV-LC–MS; and finally d) reductive cleavage of the -O–NH- bond, usually carried out under catalytic hydrogenation [15], through addition of the hydride from triethylsilane to afford **1** after in situ hydrolysis of the triethylsilyloxy bond. HOAt alone does not degrade under these conditions and intermediate **21** has been identified and characterized from the reaction medium although we did not identify 2-aminopyridine or any logical end product arising from the OAt ether degradation (Scheme 4) [16].

With these novel conditions in hand, we started the library production based on a 3-step, one-pot process: 1) amide coupling using HATU followed by removal of the solvent by sparging with nitrogen; 2) Boc deprotection with concomitant ionic reduction of the pyridine–OAt bond of the intermediate **23** was achieved by dissolving the residue in the TFA/TES/H_2_O (3:1:1 v/v/v) mixture; 3) removal of the volatiles by sparging followed by purification of the residue by mass-triggered preparative LC–MS to afford the final compounds **24**. In practice, the process was very efficient affording 60 final compounds with a median yield of 47%. Best results were obtained with electron-rich anilines (e.g., compounds **24a–d**) and aliphatic amines (e.g., compounds **24k**–**l**). For electron-deficient anilines (e.g.*,* compounds **24e–g**), the reaction mixtures were heated to 65 °C to complete the amide coupling reaction. For the least reactive anilines (e.g., compounds **24h**,**i**), HATU only afforded trace quantities of the amide even at 65 °C. However, satisfactory results were obtained by generating the acid chloride in situ by adding a 3 fold excess of POCl_3_ and heating the reaction mixtures to 60 °C over 4 h [17]. In turn, the C-2–Cl intermediates were transformed to their C-2–OBt ethers using an excess of HOBt·H_2_O in refluxing DCM to facilitate the final hydrolysis step to the desired pyridine-2-(1*H*)-one products **24h**,**i**. The low yield obtained for the 3-(2-(dimethylamino)ethyl)aniline analogue **24j** was due to the fact the 2-Cl-Py did not transform to the OAt ether in situ and the subsequent hydrolysis step was very sluggish. We postulate that the presence of the more basic *N*,*N*,-dimethylaminoethyl side chain (p*K*_a_ ≈11) must prevent protonation and activation of the pyridine towards S_N_Ar with HOAt (Table 4).

**Table 4:** Selected compounds prepared using the optimized library process.^a^

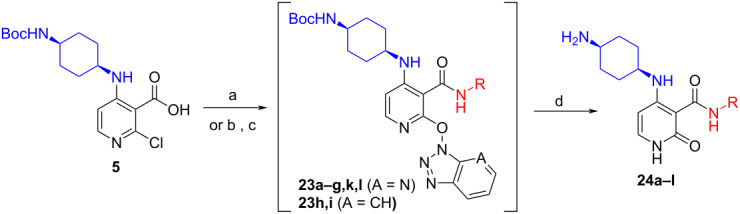		

Compound	R	Isolated yield (%)

**24a**	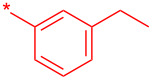	46
**24b**	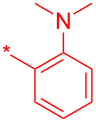	62
**24c**	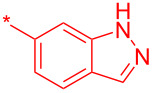	63
**24d**	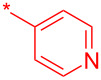	72
**24e**^b^	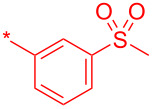	48
**24f**^b^	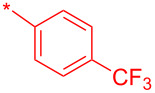	39
**24g**^b^	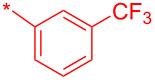	66
**24h**^c^	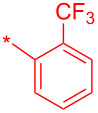	82
**24i**^c^	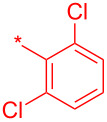	55
**24j**^d^	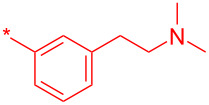	7
**24k**	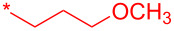	67
**24l**	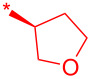	77

^a^Optimized process to delivery pyridin-2-(1*H*)-one library **24a–l**. Reagents and conditions: (a) HATU, DIPEA, DCM, rt, assumed quant.; b) POCl_3_, MeCN, rt, 60 °C, 4 h; c) HOBt·H_2_O, DCM, reflux, assumed quant.; (d) TFA/TES/H_2_O (3:1:1, 5 v/v/v), rt, 24 h, 39–82% or AcOH/H_2_O (4:1), 140 °C, microwave irradiation, 2 h, 7–55%. ^b^HATU reaction mixtures were heated to 65 °C to complete the amide-coupling reaction; ^c^compounds prepared using POCl_3_ method and AcOH/H_2_O (4:1) at 140 °C was used for step d; ^d^only the C-2–Cl intermediate was formed and AcOH/H_2_O (4:1) at 140 °C was used for step d.

### Exploration of the C-4 amine vector

**Scheme 5:**
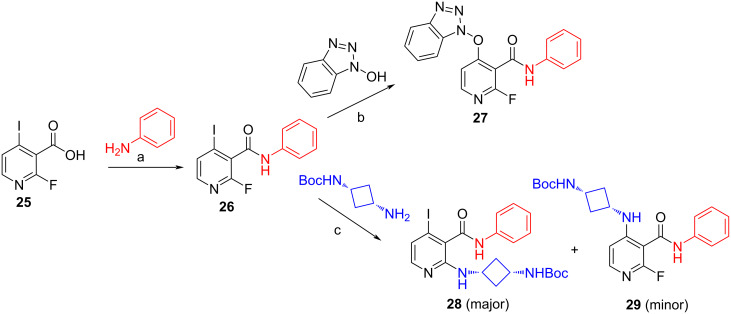
Failure to exploit logical convergent building block **26**. Reagents and conditions: a) HATU, DIPEA, DCM, rt, 16 h, 96%; b) HOBt·H_2_O, K_2_CO_3_, DMF, 80 °C, 87%; c) DIPEA, DMF, 80 °C, **28** (80%), **29** (20%).

As we moved forwards in the program, we were eager to develop our understanding of SARs (structure–activity relationships) from the C-4 vector. Although we could have adopted the same methodology as described in Scheme 5 for this exploration, we decided to focus on developing a more convergent library process than Genentech’s 3-step sequence from **2** (Scheme 1), aiming at introducing the amine moiety at C-4 at the end of the process. We envisaged a rapid, 2-step one-pot library process (amide coupling, sparging to dryness followed by C-4–I displacement) with the key pyridine-2-(1*H*)-one hinge-binding motif revealed from the beginning of the process.

Our exploration started from commercially available 2-fluoro-4-iodonicotinic acid (**25**). Amide coupling using HATU led solely to **26** with no traces of the C-2–OAt ether presumably due to the lower basicity of **26** compared to **8**, therefore, activation via protonation of the pyridine was not occurring. With a view to capitalize on our knowledge acquired so far on this scaffold, we decided to force the transformation to the OBt ether by S_N_Ar under basic conditions. To our surprise, the iodide at C-4 was selectively displaced to afford only **27**. We propose that this observation can be explained by HSAB (hard-soft-acid-base) theory whereby the soft ^–^OBt anion preferentially reacts at the soft C-4–I center [18]. As expected, reacting **26** first with *tert*-butyl ((*cis*)-3-aminocyclobutyl)carbamate afforded the undesired regioisomer **28** as the majority product (Scheme 5).

**Scheme 6:**
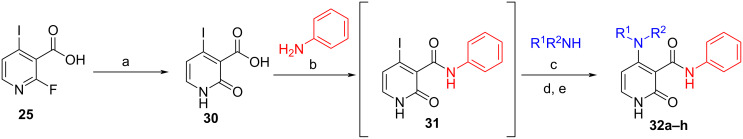
Library route to **32**. Reagents and conditions: a) 4 M HCl_aq_, reflux, 1 h, 81%; (b) EDCI, pyridine, Pfp-OH, DMF, rt, 80%; b) RNH_2_ (3–5 equiv), DIPEA, DMF, 60 °C, 16 h, 44–71% **32a–h**); d) TFA, rt, 30 min. for Boc protected amines (**32b**,**c**); e) Pd(OAc)_2_, TES, DCM/MeOH, rt, 16 h for Cbz protected amines.

As we were unable to exploit intermediate **26**, we turned our attention to the pyridine-2-(1*H*)-one building block **30**, easily prepared by hydrolysis of the C-2–F bond in high yield and without the need for chromatography [19]. Amide coupling was best carried out under acidic coupling conditions by pre-activating **30** using EDCI, pentylfluorophenol (Pfp-OH) in the presence of one equivalent pyridine and adding aniline dropwise over a few minutes. Under basic coupling conditions (e.g., HATU, DIPEA), complex mixtures were obtained with less than 5% conversion to the desired amide product **31**. The library process was completed by carrying out the nucleophilic aromatic substitution with a 3-fold excess of amine in DMF at 60 °C overnight to afford **32a–h**, after a removal of Boc or Cbz groups [20] where present (Scheme 6).

**Table 5:** Selected compounds prepared using the optimized library process to explore the C-4 vector.

Compound	R^1^	R^2^	Isolated yield (%)

**32a**	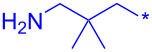	H	44
**32b**	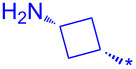	H	56
**32c**	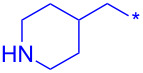	H	57
**32d**	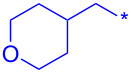	H	55
**32e**	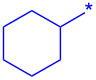	H	66
**32f**	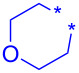		71
**32g**	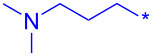	H	55
**32h**	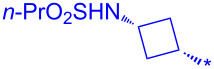	H	59

## Conclusion

In conclusion, we have developed novel, complementary multi-parallel synthetic routes permitting the exploitation of the C-3 then C-4 vectors or vice versa to deliver our library of novel 3,4-disubstituted pyridin-2(1*H*)-one kinase inhibitors starting from readily-available 2-chloro-4-fluoronicotinic acid and 2-fluoro-4-iodonicotinic acid, respectively. Perhaps the highlight of our library route development was the novel transformation to the desired pyridin-2(1*H*)-one motif via in situ formation of the C2–OAt ether during HATU coupling and its cleavage under ionic hydrogenation conditions at just room temperature.

## Supporting Information

File 1 Detailed experimental protocols and supporting ^1^H, ^13^C NMR, LC–MS characterization data and spectra for all compounds.
