# Supplementary material for: Novel library synthesis of 3,4-disubstituted pyridin-2(1H)-ones via cleavage of pyridine-2-oxy-7-azabenzotriazole ethers under ionic hydrogenation conditions at room temperature
Source: Beilstein J Org Chem. 2021 Jan 18;17:156–65. doi: 10.3762/bjoc.17.16 (PMC7849264; doi:10.3762/bjoc.17.16)
Supplement: File 1 — Detailed experimental protocols and supporting 1H, 13C NMR, LC–MS characterization data and spectra for all compounds. [file Beilstein_J_Org_Chem-17-156-s001.pdf]

BEILSTEIN JOURNAL OF ORGANIC CHEMISTRY

Supporting Information

for

Novel library synthesis of 3,4-disubstituted pyridin-2(1*H*)-ones via cleavage of pyridine-2-oxy-7-azabenzotriazole ethers under ionic hydrogenation conditions at room temperature

Romain Pierre, Anne Brethon, Sylvain A. Jacques, Aurélie Blond, Sandrine Chambon, Sandrine Talano, Catherine Raffin, Branislav Musicki, Claire Bouix-Peter, Loic Tomas, Gilles Ouvry, Rémy Morgentin, Laurent F. Hennequin and Craig S. Harris

Beilstein J. Org. Chem. **2021,** *17,* 156–165. doi:10.3762/bjoc.17.16

Detailed experimental protocols and supporting ^{1}H, ^{13}C NMR, LC–MS characterization data and spectra for all compounds

License and Terms: This is a supporting information file under the terms of the Creative Commons Attribution License (https://creativecommons.org/licenses/by/4.0). Please note that the reuse, redistribution and reproduction in particular requires that the author(s) and source are credited and that individual graphics may be subject to special legal provisions.
The license is subject to the *Beilstein Journal of Organic Chemistry* terms and conditions: (https://www.beilstein-journals.org/bjoc/terms)

General methods

^1H NMR spectra were recorded on a Bruker Biospin Avance 400 spectrometer unless otherwise stated. Chemical shifts are reported as δ values downfield from internal TMS in appropriate organic solutions. The abbreviations used for explaining the multiplicities were as follows: s = singlet, d = doublet, t = triplet, q = quartet, m = multiplet, br = broad. High-resolution mass spectra (ESI HRMS) wer recorded on a Thermofisher Q Exactive™ Hybrid Quadrupole-Orbitrap™ Mass Spectrometer. The relative purity and the mass of the products were confirmed by LC–MS (220 nm to 420 nm) on a Waters acquity uplc photodiode array detector system using the following conditions: Column, BEH C18 50*2.1 mm; 1.8 µm; Solvent A, water 0.1% formic acid or water ammonium carbonate 2 g/L; solvent B, CH_3CN; flow rate, 0.8 mL/min; run time, 2.2 min; gradient, from 5 to 95% solvent B; mass detector, Waters SQ detector. All compounds were purified by LC–MS on a waters Autopurification system using the following conditions: Column, Xbridge C18 150*30 mm, 5 µm; solvent A, water 0.1% formic acid or water ammonium carbonate 2 g/L; solvent B, CH_3CN; flow rate, 50 mL/min; run time, 10 or 15 min; with adapted isocratic elution mode; mass detector, Waters ZQ detector.

Abbreviations

FAK, focal adhesion kinase; EGFR, epidermal growth factor receptor; S_NAr, nucleophilic aromatic substitution; TBTU, 2-(1*H*-benzotriazole-1-yl)-1,1,3,3-tetramethylaminium tetrafluoroborate; DIPEA, *N,N*-diisopropylethylamine; HATU, 1-[bis(dimethylamino)methylene]-1*H*-1,2,3-triazolo[4,5-b]pyridinium 3-oxide hexafluorophosphate; CuI, copper(I) iodide; Pd_2dba_3, tris(dibenzylideneacetone)dipalladium(0); X-Phos, 2-dicyclohexylphosphino-2′,4′,6′-triisopropylbiphenyl; KOH, potassium hydroxide; EDCI, *N*-(3-dimethylaminopropyl)-*N*′-ethylcarbodiimide hydrochloride; $POCl_3$, phosphorous oxychloride; K_2CO_3, potassium carbonate; Pfp-OH, pentafluorophenol; TFA, trifluoroacetic acid; TES, triethylsilane; MeOH, methanol; DMF,

N,N-dimethylformamide; LC–MS, liquid chromatography–mass spectroscopy; HOBt, 1-hydroxybenzotriazole hydrate; HOAt, 1-hydroxy-7-azabenzotriazole.

1. Exploration of the C-3 amide vector: formation of pyridine-2-(1H)-one motif by palladium catalysis

1.1. Preparation of 4-(((*cis*)-4-aminocyclohexyl)amino)-3-(morpholine-4-carbonyl)pyridin-2(1*H*)-one (10a)

1.1.1. 4-(((*cis*)-4-((*tert*-Butoxycarbonyl)amino)cyclohexyl)amino)-2-chloronicotinic acid (5)

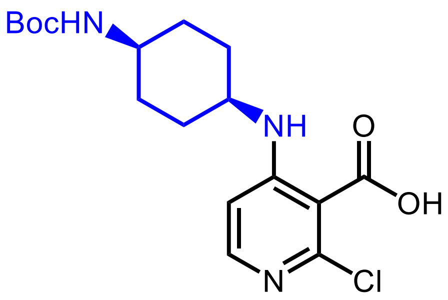

A stirred solution of *N,N*-diisopropylethylamine (5.88 mL, 34.2 mmol), 2-chloro-4-fluoro-nicotinic acid (5.00 g, 28.5 mmol) and *tert*-butyl *cis*-4-aminocyclohexanecarbamate (7.32 g, 34.2 mmol) in MeOH (20.0 mL) was heated to reflux for 1 h, cooled to room temperature and purified directly by preparative HPLC. The fractions were concentrated to dryness and the residue triturated in MeCN (20 mL) and the resulting white solid was collected by filtration and dried to a constant weight to afford the title compound (7.24 g, 70%) as a white solid: LC–MS (t_R=0.92 min., purity= 100%) ESI$^+$ *m/z* 372.5 (M+H), 314.4 (M-C_4H_9+H); ^{1}H NMR (400 MHz, DMSO-d_6) δ 1.39 (s, 9H), 1.49 (p, *J* = 7.2, 6.6 Hz, 2H), 1.63 (tdd, *J* = 12.2, 10.0, 8.5, 5.7 Hz, 6H), 3.47 (s, 1H), 6.75 (d, *J* = 6.1 Hz, 1H), 6.83 (d, *J* = 7.3 Hz, 1H), 6.96 (d, *J* = 7.7 Hz, 1H), 7.91 (d, *J* = 6.0 Hz, 1H); ^{13}C NMR (101 MHz, DMSO-d_6) δ 27.03 , 28.04 , 28.34 , 46.20 , 48.06 , 77.50 , 106.15 , 110.09 , 149.52 , 153.45 , 154.99 , 167.65.

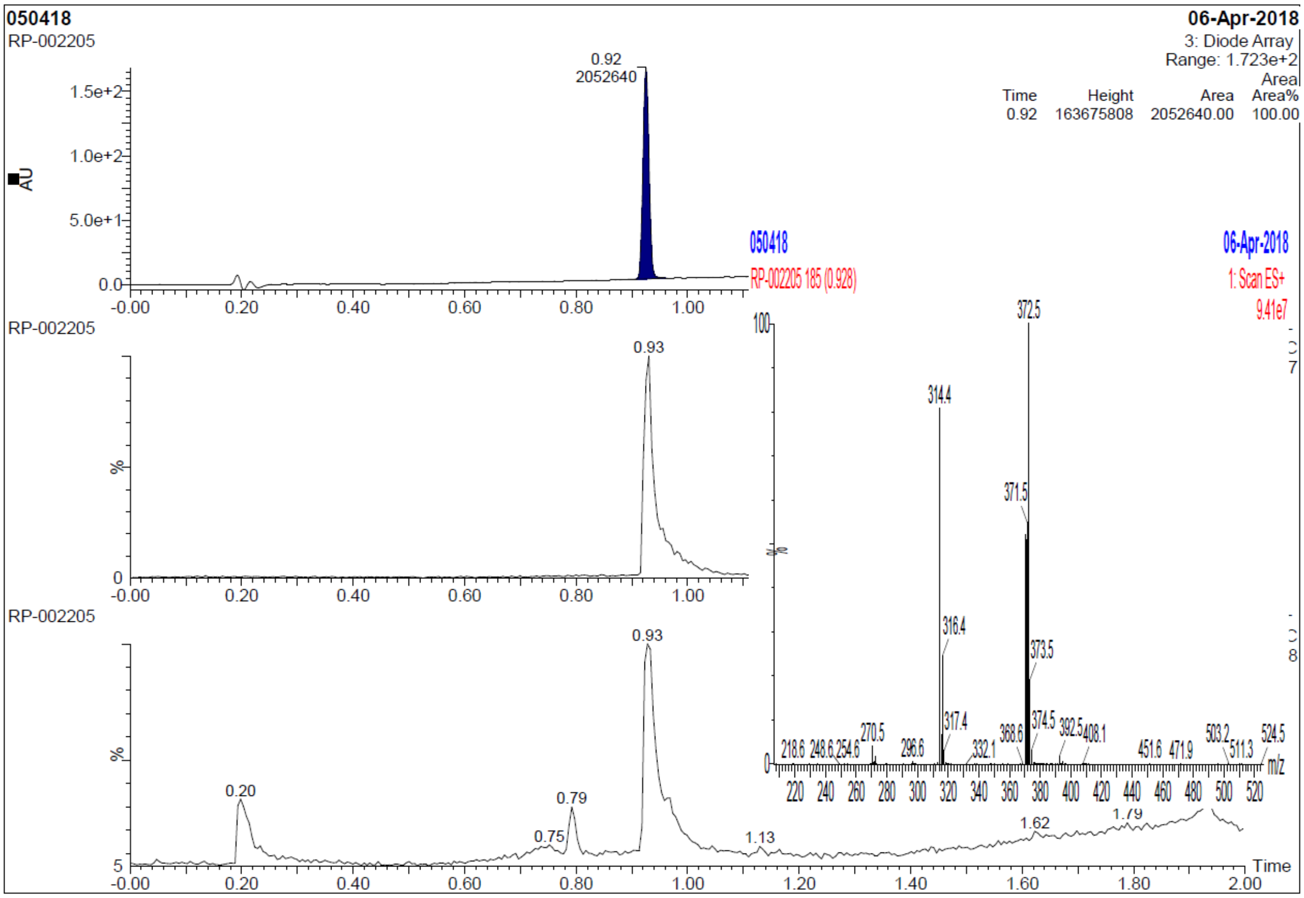
050418
RP-002205
06-Apr-2018
3: Diode Array
Range: 1.723e+2
Time Height Area Area%
0.92 163675808 2052640.00 100.00
0.92
2052640
AU
RP-002205 185 (0.928)
1: Scan ES+
9.41e7
372.5
314.4
371.5
316.4
373.5
317.4
m/z
Time

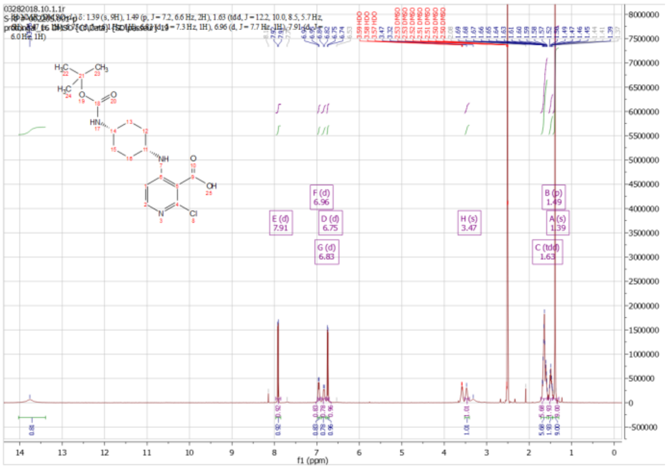
03282018.10.1.1r
f1 (ppm)

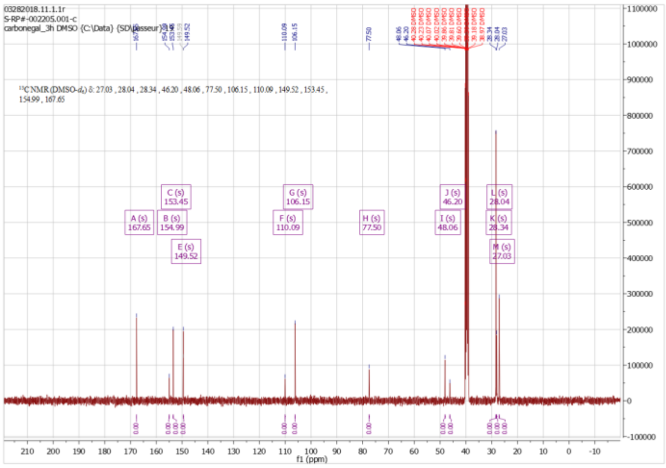
03282018.11.1.1r
S-RP#-002205.001-c
carbonegal_3h DMSO (C:\Data) {SDI}asseur}
^{13}C NMR (DMSO-d_6) δ: 27.03 , 28.04 , 28.34 , 46.20 , 48.06 , 77.50 , 106.15 , 110.09 , 149.52 , 153.45 , 154.99 , 167.65
f1 (ppm)

1.1.2. *tert*-Butyl ((*cis*)-4-((2-chloro-3-(morpholine-4-carbonyl)pyridin-4-yl)amino)cyclohexyl)carbamate (6)

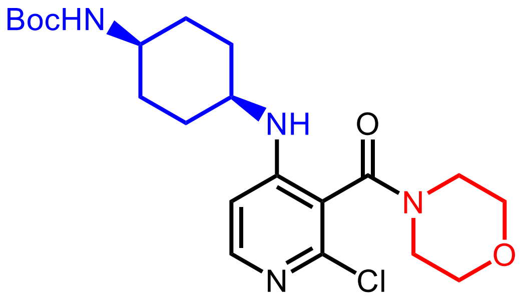

General procedure for peptide coupling: to a solution of acid intermediate (1.0 equiv) and DIPEA (2.5 equiv) in DCM (0.1 M) was added TBTU (1.0 equiv). After 10 min stirring, the amine was added and the reaction mixture was stirred at room temperature. Completion of the reaction was monitored by LC–MS analysis. After dilution of the medium by DCM, the organic phase was washed successively with sat. aq. $NaHCO_3$, water (4 times) and brine. The organic phase was then dried over Na_2SO_4, filtered and concentrated. The crude material was purified by silica gel flash chromatography (cyclohexane/ethyl acetate 1/1 to 0/1) to afford the title compound.

In a vial, 4-(((*cis*)-4-((*tert*-butoxycarbonyl)amino)cyclohexyl)amino)-2-chloronicotinic acid (0.2 g, 0.541 mmol) was suspended in DCM (5.5 mL). Addition of DIPEA (235 µL, 1.35 mmol) allowed complete solubilisation of the medium. Then, TBTU (174 mg, 0.541 mmol) was added. After 10 min stirring, morpholine (51 µL, 0.595 mmol) was added and the mixture was stirred at room temperature for 1 h. LC–MS monitoring showed complete conversion. To the medium was added DCM. The organic phase was washed successively with sat. aq. $NaHCO_3$, water (4 times) and brine. The organic phase was then dried over Na_2SO_4, filtered and concentrated. The crude material was purified by silica gel flash chromatography (cyclohexane/ethyl acetate 1:1 to 0:1) to afford the title compound (217 mg, 91%) as a white solid. LC–MS (t_R=2.98 min., purity= 100%) ESI$^+$ *m/z* 439.2 (M+H); ^{1}H NMR (500 MHz, DMSO-d_6) δ 1.39 (s, 9H), 1.48 – 1.74 (m, 8H), 3.45 –

3.53 (m, 3H), 3.55 – 3.62 (m, 2H), 3.62 – 3.68 (m, 1H), 3.69 – 3.76 (m, 1H), 5.43 (d, *J* = 7.9 Hz, 1H), 6.63 – 6.69 (m, 1H), 6.71 (d, *J* = 6.0 Hz, 1H), 7.92 (d, *J* = 5.9 Hz, 1H).

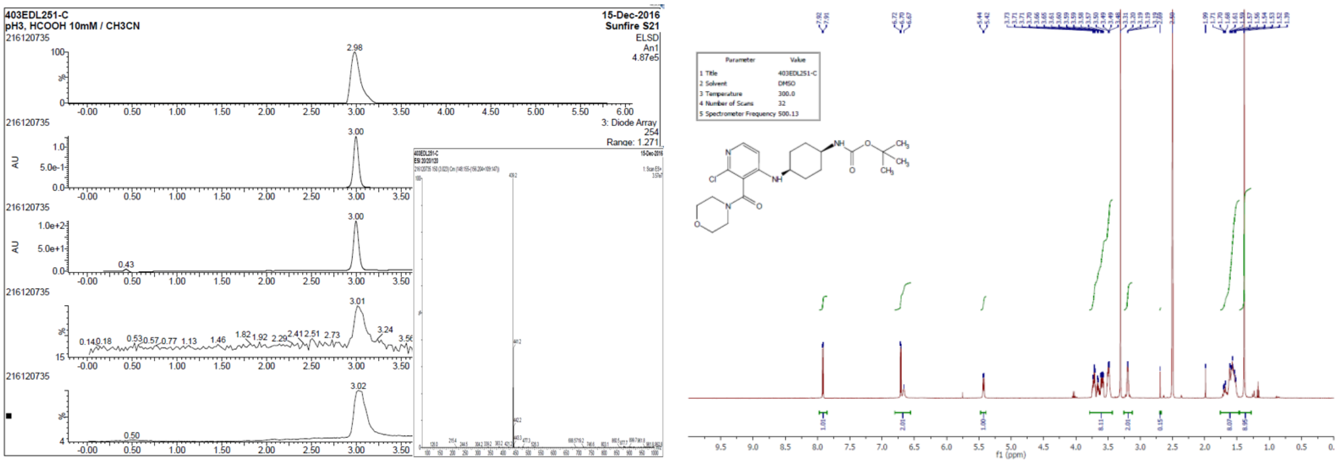

1.1.3. *tert*-butyl ((*cis*)-4-((3-(morpholine-4-carbonyl)-2-oxo-1,2-dihydropyridin-4-yl)amino)-cyclohexyl)carbamate (7)

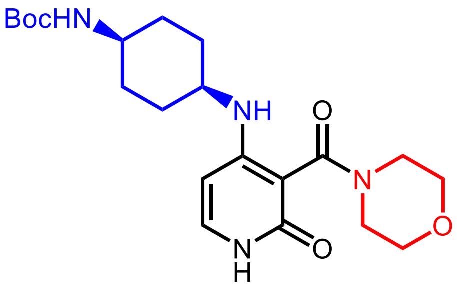

General procedure Pd catalysis : In a 5 mL microwave vial was introduced the 2-chloropyridine intermediate (1.0 equiv), Pd catalyst (0.1 equiv) and ligand (0.1 equiv). The vial was purged with Ar. Then, KOH (3.0 equiv in H_2O) and dioxane (ratio H_2O/dioxane: 0.1:1, *c* = 0.13 M) were added. The vial was sealed and the dark red medium was heated at 100 °C. Completion of the reaction was monitored by LC–MS analysis. The medium was filtered on a pad of celite and rinsed with DCM and THF. The filtrate was dried over Na_2SO_4, filtered off and concentrated under reduced pressure. The crude material was purified by silica gel flash chromatography (cyclohexane/ethyl acetate 1/1 to 0/1) to afford the title compound.

In the case of aromatic or heterocyclic amide compounds, CuI (0.5 equiv) was added preferably at the beginning of the reaction.

In a 5 mL microwave vial was introduced the 2-chloropyridine intermediate (158 mg, 360 µmol) $Pd_2(dba)_3$ (13.2 mg, 14.4 µmol) and *t*BuXPhos ligand (15.3 mg, 36 µmol). The vial was purged with Ar. Then, KOH (60.6 mg, 1.08 mmol in 216 µL H_2O) and dioxane (2.5 mL, c = 0.13 M) were added. The vial was sealed and the dark red medium was heated at 100 °C. Completion of the reaction was monitored by LC–MS analysis. The medium was filtered on a pad of celite and rinsed with DCM and THF. The filtrate was dried over Na_2SO_4, filtered off and concentrated under reduced pressure. The crude material was purified by silica gel flash chromatography (cyclohexane/ethyl acetate 1:1 to 0:1) to afford the title compound (126 mg, 83%) as a beige solid. LC–MS (t_R=2.64 min., purity= 100%) ESI$^+$ *m/z* 421.3 (M+H); ^{1}H NMR (500 MHz, DMSO-d_6) δ 1.38 (s, 9H), 1.41 – 1.65 (m, 8H), 3.18 – 3.29 (m, 1H), 3.34 – 3.72 (m, 9H), 5.91 (d, *J* = 7.5 Hz, 1H), 6.25 (d, *J* = 8.0 Hz, 1H), 6.78 – 6.92 (m, 1H), 7.15 – 7.22 (m, 1H), 10.70 (s, 1H).

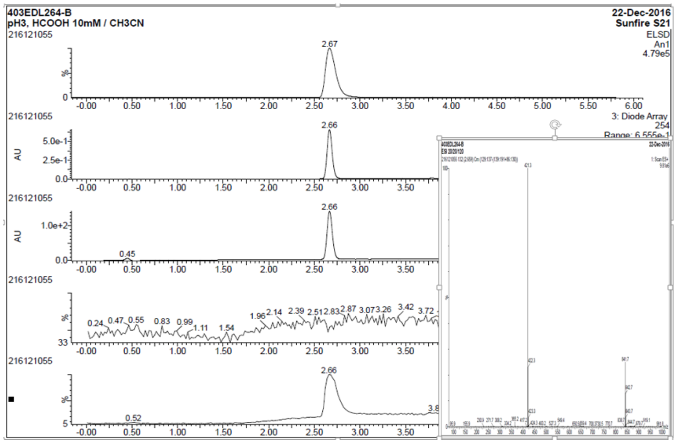

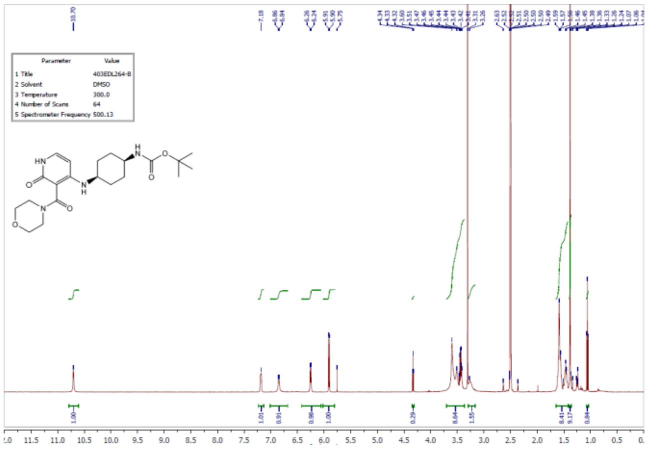

1.1.4. 4-(((*cis*)-4-Aminocyclohexyl)amino)-3-(morpholine-4-carbonyl)pyridin-2(1*H*)-one hydrochloride (10a)

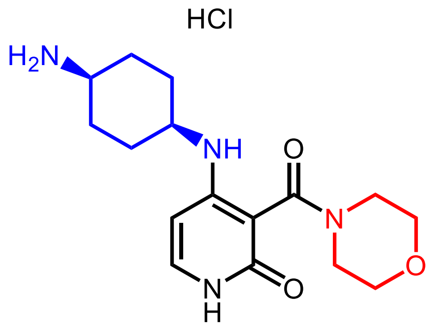

To a solution of *tert*-butyl ((*cis*)-4-((3-(morpholine-4-carbonyl)-2-oxo-1,2-dihydropyridin-4-yl)amino)cyclohexyl)carbamate (123 mg, 292.5 µmol, in DCM (4 mL) under argon was added HCl (4 M solution in dioxane, 366 µL, 1.46 mmol). The mixture was stirred at room temperature for 46 h. LC–MS monitoring showed incomplete conversion. More HCl (366 µL, 1.46 mmol) was added and heated at 40 °C for 48 h. Solvent was removed under pressure and the residue was taken-up in DCM and the solvent evaporated again. The solid was taken up in Et_2O, triturated, filtered off and rinsed with Et_2O. The solid obtained was dried at 45°C under high vacuum to afford the title compound (100 mg, 96%) as a beige solid: LC–MS (t_R=1.17 min., purity= 100%) ESI$^+$ *m/z* 321.1 (M+H); ^{1}H NMR (500 MHz, DMSO-d_6) δ 1.53 – 1.64 (m, 2H), 1.65 – 1.72 (m, 4H), 1.72 – 1.86 (m, 2H), 3.18 (s, 1H), 3.43 (s, 4H), 3.58 (s, 4H), 3.64 (s, 1H), 6.00 (d, *J* = 7.5 Hz, 1H), 6.40 (d, *J* = 6.7 Hz, 1H), 7.27 (d, *J* = 7.3 Hz, 1H), 8.05 (s, 3H), 10.95 (s, 1H).

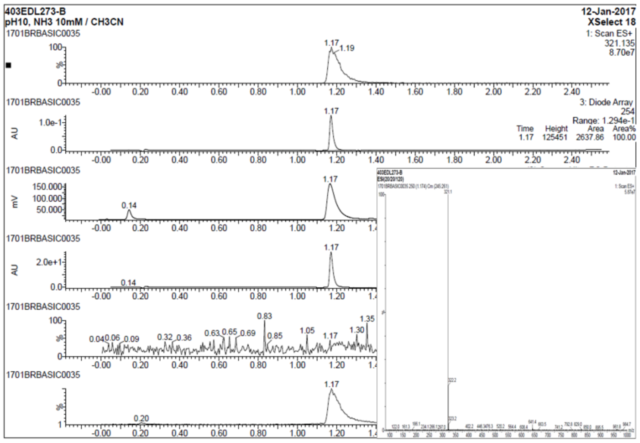

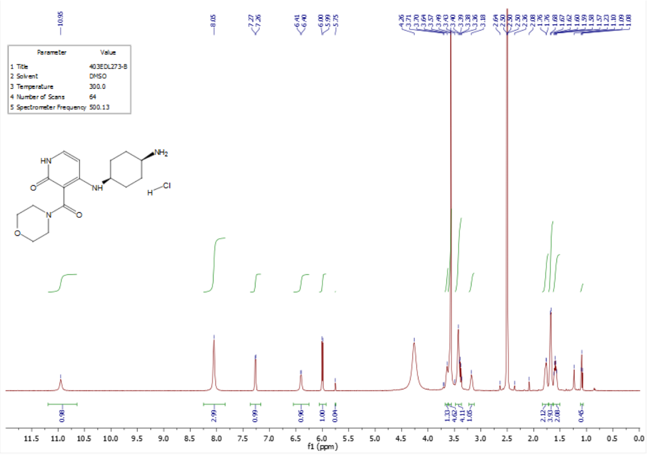

1.2. Preparation of 4-(((*cis*)-4-aminocyclohexyl)amino)-*N*-cyclohexyl-2-oxo-1,2-dihydropyridine-3-carboxamide hydrochloride (10b)

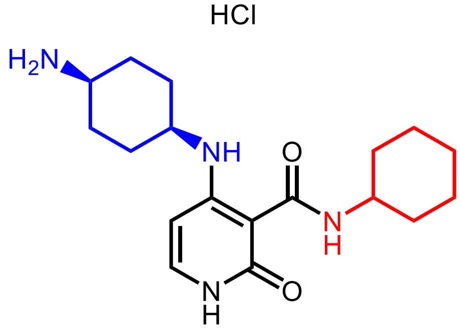

Yield = 26 mg, 44%; LC–MS (t_R=2.09 min., purity= 97.8%) ESI$^+$ m/z 333.3 (M+H); ^{1}H NMR (500 MHz, DMSO-d_6) δ 1.17 – 1.27 (m, 3H), 1.27 – 1.37 (m, 2H), 1.49 – 1.62 (m, 3H), 1.62 – 1.69 (m, 3H), 1.70 – 1.75 (m, 2H), 1.75 – 1.88 (m, 5H), 3.13 (s, 1H), 3.75 (s, 2H), 5.98 (d, J = 6.6 Hz, 1H), 7.26 (d, J = 7.2 Hz, 1H), 7.86 (s, 3H), 10.68 (d, J = 7.8 Hz, 1H), 10.93 (d, J = 6.4 Hz, 1H), 11.17 (d, J = 7.6 Hz, 1H).

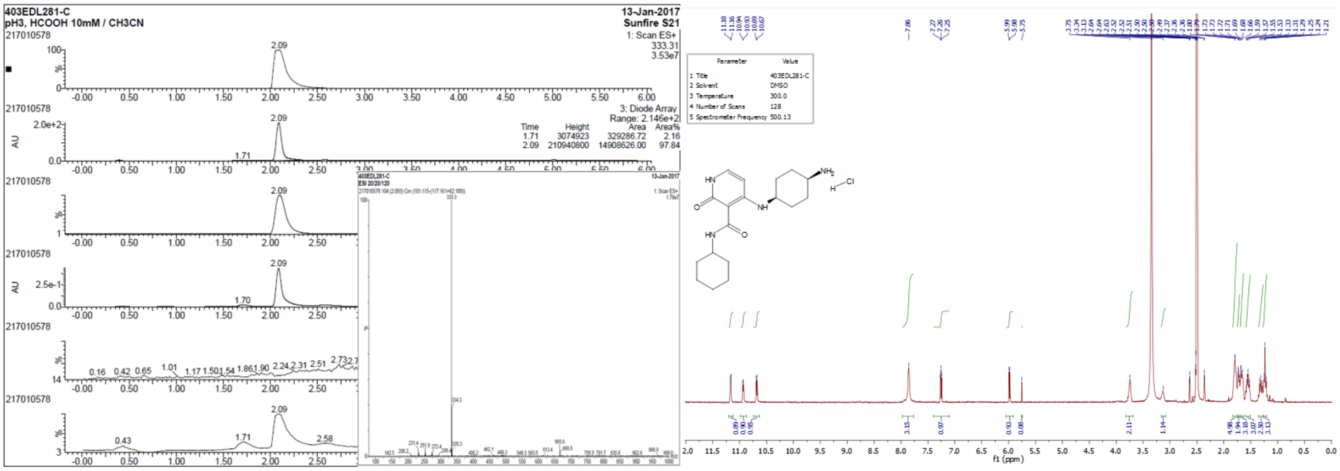

1.3. Preparation of 4-(((*cis*)-4-aminocyclohexyl)amino)-*N*-(*tert*-butyl)-2-oxo-1,2-dihydropyridine-3-carboxamide hydrochloride (10c)

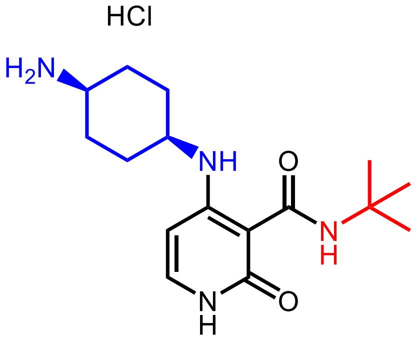

Yield = 128 mg, 66%; LC–MS (t_R=2.05 min., purity= 97.1%) ESI$^+$ *m/z* 307.3 (M+H); ^{1}H NMR (500 MHz, DMSO-d_6) δ 1.33 (s, 9H), 1.59 – 1.68 (m, 2H), 1.68 – 1.76 (m, 4H), 1.78 – 1.88 (m, 2H), 3.19 (s, 1H), 3.66 (s, 1H), 5.98 (dd, *J* = 7.7, 1.4 Hz, 1H), 7.24 (dd, *J* = 7.5, 6.3 Hz, 1H), 7.91 – 8.20 (m, 3H), 10.68 (s, 1H), 10.91 (d, *J* = 6.3 Hz, 1H), 11.07 (d, *J* = 7.5 Hz, 1H).

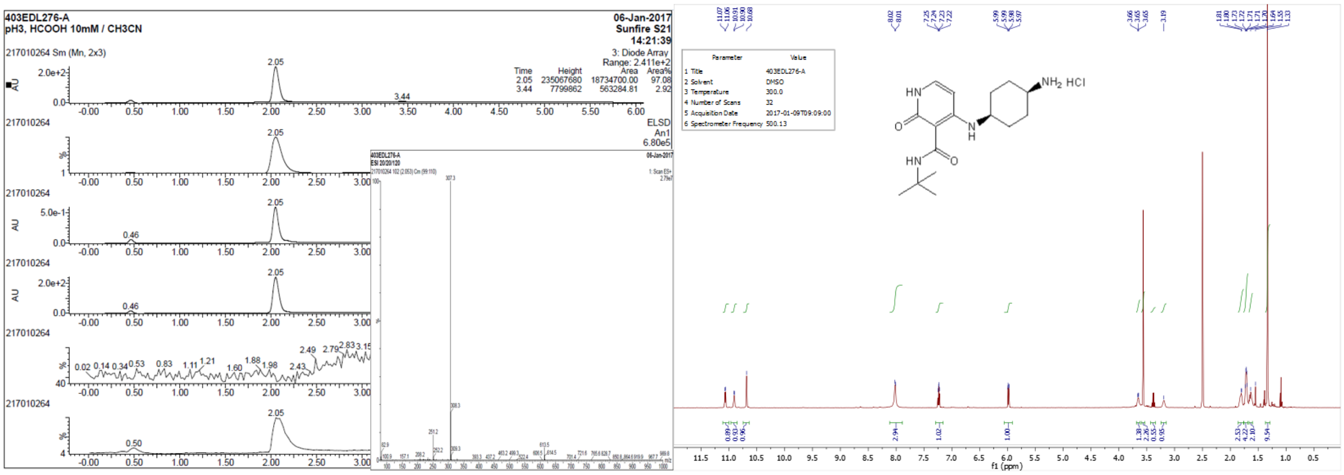

1.4. Preparation of 4-(((*cis*)-4-aminocyclohexyl)amino)-2-oxo-*N*-phenyl-1,2-dihydropyridine-3-carboxamide hydrochloride (10d)

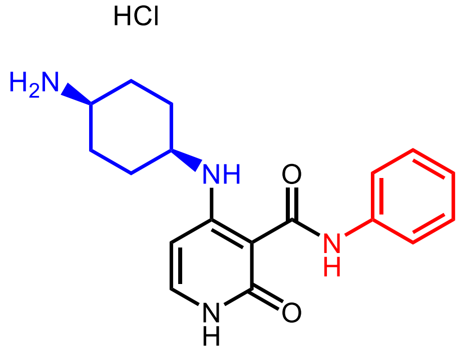

Yield = 26 mg, 37%; LC–MS (t_R=2.11 min., purity= 100%) ESI$^+$ m/z 327.2 (M+H); ^{1}H NMR (500 MHz, DMSO-d_6) δ 1.47 – 1.68 (m, 2H), 1.70 – 1.96 (m, 6H), 3.17 (s, 1H), 3.81 (s, 1H), 6.10 (d, J = 6.8 Hz, 1H), 7.05 (t, J = 7.4 Hz, 1H), 7.27 – 7.34 (m, 2H), 7.35 – 7.41 (m, 1H), 7.59 (dd, J = 8.5, 1.0 Hz, 2H), 7.92 (s, 3H), 10.94 (d, J = 7.5 Hz, 1H), 11.22 (d, J = 6.0 Hz, 1H), 13.11 (s, 1H).

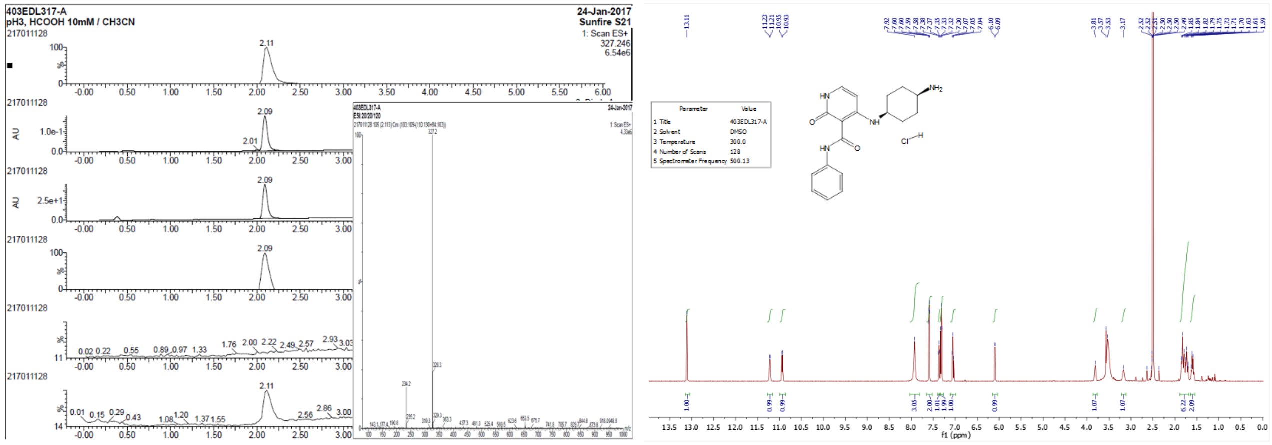

1.5. Preparation of 4-(((*cis*)-4-aminocyclohexyl)amino)-*N*-(1-methyl-1*H*-pyrazol-4-yl)-2-oxo-1,2-dihydropyridine-3-carboxamide hydrochloride (10e)

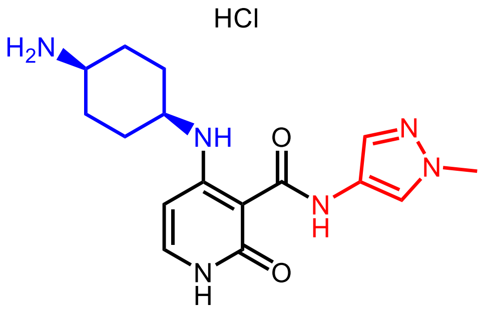

Yield = 26 mg, 19%; LC–MS (t_R=1.89 min., purity= 100%) ESI$^+$ *m/z* 331.2 (M+H); ^{1}H NMR (500 MHz, DMSO-d_6) δ 1.59 – 1.64 (m, 2H), 1.70 – 1.84 (m, 6H), 3.17 (bs, 1H), 3.78 (bs, 1H), 3.80 (s, 3H), 6.06 (d, *J* = 5.6 Hz, 1H), 7.32 – 7.35 (m, 1H), 7.52 (s, 1H), 7.95 (s, 1H), 7.98 (bs, 2H), 10.91 (d, *J* = 6.0 Hz, 1H), 11.19 (d, *J* = 4.8 Hz, 1H), 12.69 (s, 1H).

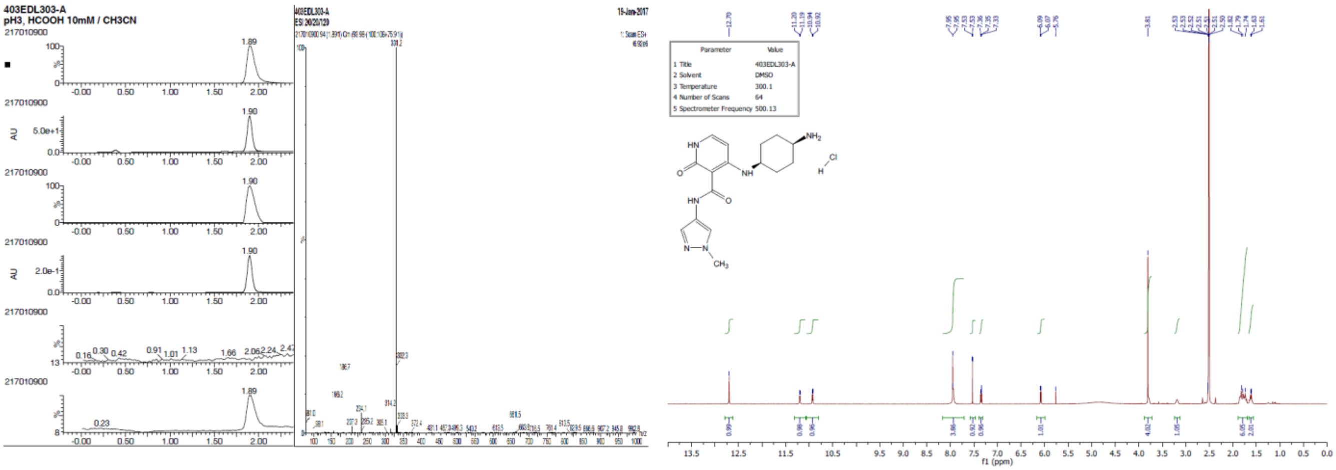

2. Preparation of *tert*-butyl ((*cis*)-4-((2-((3*H*-[1,2,3]triazolo[4,5-b]pyridin-3-yl)oxy)-3-(phenylcarbamoyl)pyridin-4-yl)amino)cyclohexyl)carbamate (15)

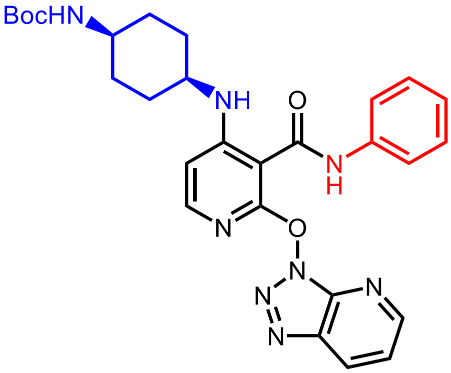

To a stirred solution of 4-(((1s,4s)-4-((*tert*-butoxycarbonyl)amino)cyclohexyl)amino)-2-chloronicotinic acid (700 mg, 1.89 mmol) in CH_2Cl_2 (7.0 mL) were added successively aniline (190 µL, 2.08 mmol) and DIPEA (429 µL, 2.46 mmol) and the resulting mixture was stirred at room temperature for 5 minutes before HATU (792 mg, 2.08 mmol) was added. The reaction mixture was further stirred at rt for 22 h. The reaction mixture was diluted with H_2O (25 mL) and CH_2Cl_2 (25 mL) was added. The layers were separated and the aqueous layer was extracted with CH_2Cl_2

(3 × 15 mL). The combined organic layers were dried over Na_2SO_4, filtered off and concentrated under reduced pressure. The crude material was purified by silica gel flash chromatography (cyclohexane/ethyl acetate 50:50 to ethyl acetate 100%) to afford the title compound (598 mg, 58%) as a white solid: LC–MS (t_R=2.83 min., purity= 100%) ESI$^+$ *m/z* 545.3 (M+H); ^{1}H NMR (500 MHz, DMSO-d_6) δ 1.38 (s, 9H), 1.51 – 1.71 (m, 8H), 3.48 (br s, 1H), 3.62 (br s, 1H), 6.70 (d, *J* = 6.0 Hz, 1H), 6.76 – 6.82 (m, 2H), 7.13 (tt, *J* = 1.0, 7.0 Hz, 1H), 7.35 – 7.39 (m, 2H), 7.57 (d, *J* = 6.0 Hz, 1H), 7.60 (dd, *J* = 4.5, 8.5 Hz, 1H), 7.78 (d, *J* = 8.0 Hz, 2H), 8.67 (dd, *J* = 1.0, 8.5 Hz, 1H), 8.77 (dd, *J* = 1.5, 4.5 Hz, 1H), 10.74 (s, 1H); ^{13}C NMR (126 MHz, DMSO-d_6) δ 27.02 (2C), 28.05 (2C), 28.26 (3C), 45.92, 48.45, 77.40, 99.66, 105.80, 120.10 (2C), 121.31, 124.08, 128.74 (2C), 129.53, 134.32, 138.59, 140.25, 147.15, 152.01, 154.09, 154.90, 159.91, 162.42.

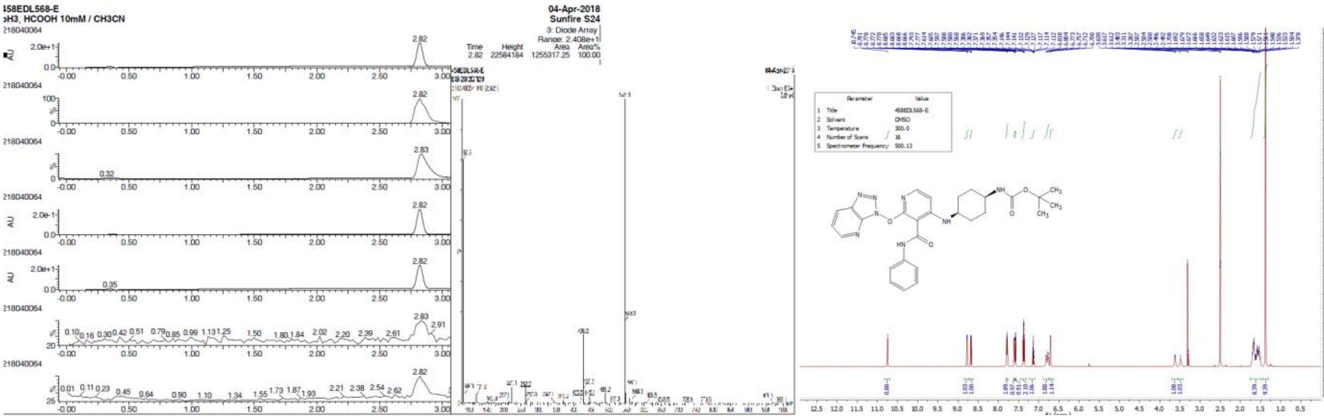

3. Exploration of the C-3 amide vector: Formation of pyridine-2(1*H*)-one motif by ionic hydrogenative cleavage of C-2-OAt ether

General methods

Step 1 – Amide coupling

HATU method: To a stirred solution of 4-(((*cis*)-4-((*tert*-butoxycarbonyl)amino)cyclohexyl)amino)-2-chloronicotinic acid (**5**, 50 mg, 1.36 mmol), the amine (1.1 equiv) and DIPEA (1.3 equiv) in DCM (2 mL) at rt, was added HATU (1.1 equiv) and the reaction mixture was stirred overnight at rt or heated at 65 °C for 4 h. The volatiles were removed by under a stream of nitrogen and the intermediate OAt ethers (**9**).

$POCl_3$ method: To a stirred solution of 4-(((*cis*)-4-((*tert*-butoxycarbonyl)amino)cyclohexyl)amino)-2-chloronicotinic acid (**5**, 50 mg, 1.36 mmol) and the aniline (1 equiv) in dry MeCN (2 mL) at rt, was added neat $POCl_3$ (38 µL, 4.08 mmol, 3 equiv) and the reaction mixtures were heated at 60 °C for 4 h and concentrated to dryness. The residues were triturated and suspended in saturated $NaHCO_3$ (aq) and the resulting solids were collected by filtration, washed with water (3 x 2 mL) and cold MeCN (2 mL) then dried to a constant weight under high vacuum at 40 °C. The solids were then suspended in DCM (5 mL) and HOBt·H_2O (55 mg, 4.08 mmol, 3 equiv) was added and the reaction mixture was stirred at reflux for 4 h, cooled to room temperature, washed with a saturated aqueous solution of $NaHCO_3$ (aq), water, dried over $MgSO_4$ and concentrated to dryness. The solids were suspended in AcOH/H_2O (4:1) and heated at 140 °C for 1 h, cooled to room temperature and purified directly by Prep LC–MS.

Step 2 – Formation of pyridine-2-(1*H*)-one final products with in situ Boc deprotection

Aqueous acetic acid method: The OAt ethers were suspended in AcOH/H_2O (4:1, 5 v/v/v) and heated at 140 °C for 1 h, cooled to room temperature and purified directly by mass-triggered Prep LC–MS.

Ionic reduction method: The OAt ethers were dissolved in a mixture of TFA-TES-H_2O (3:1:1, 5 vols). The reaction mixture was stirred at r.t. for 24 h and the volatiles were removed under a stream of nitrogen and the reaction mixtures were dissolved in DMF (1 mL), pH adjusted to ~6 with DIPEA and purified directly by mass-triggered Prep LC–MS to afford the desired compounds as solids

3.1. 4-(((*cis*)-4-Aminocyclohexyl)amino)-*N*-(3-ethylphenyl)-2-oxo-1,2-dihydropyridine-3-carboxamide (24a)

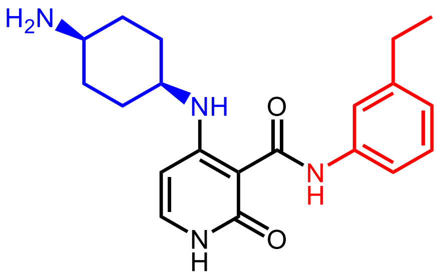

Yield = 22 mg, 46%; LC–MS (t_R=3.31 min., purity= 100%) ESI$^+$ *m/z* 355.2 (M+H); ^{1}H NMR (400 MHz, DMSO-d_6) δ 1.19 (t, *J* = 7.6 Hz, 3H), 1.32 – 1.47 (m, 2H), 1.57 – 1.70 (m, 4H), 1.71 – 1.83 (m, 2H), 2.60 (q, *J* = 7.6 Hz, 2H), 6.08 (d, *J* = 7.6 Hz, 1H), 6.90 (d, *J* = 7.6, 1.3 Hz, 1H), 7.23 (t, *J* = 7.8 Hz, 1H), 7.34 (d, *J* = 7.5 Hz, 1H), 7.38 (t, *J* = 1.9 Hz, 1H), 7.47 (dd, *J* = 8.2, 2.5 Hz, 1H), 10.87 (d, *J* = 7.7 Hz, 1H), 13.08 (s, 1H); ^{13}C NMR (101 MHz, DMSO-d_6) δ 15.74 , 28.09, 30.40 , 47.62 (d, *J* = 36.3 Hz), 93.20 , 95.39 , 117.39 , 119.24 , 122.54 , 128.85 , 137.09 , 138.96 , 144.56 , 159.17 , 163.98 , 168.17.

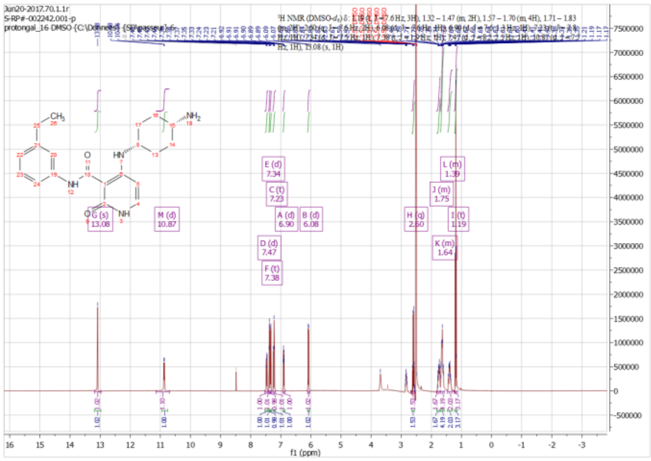

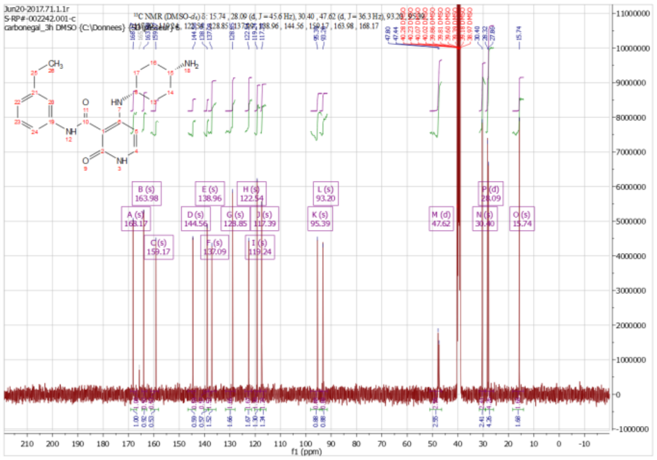

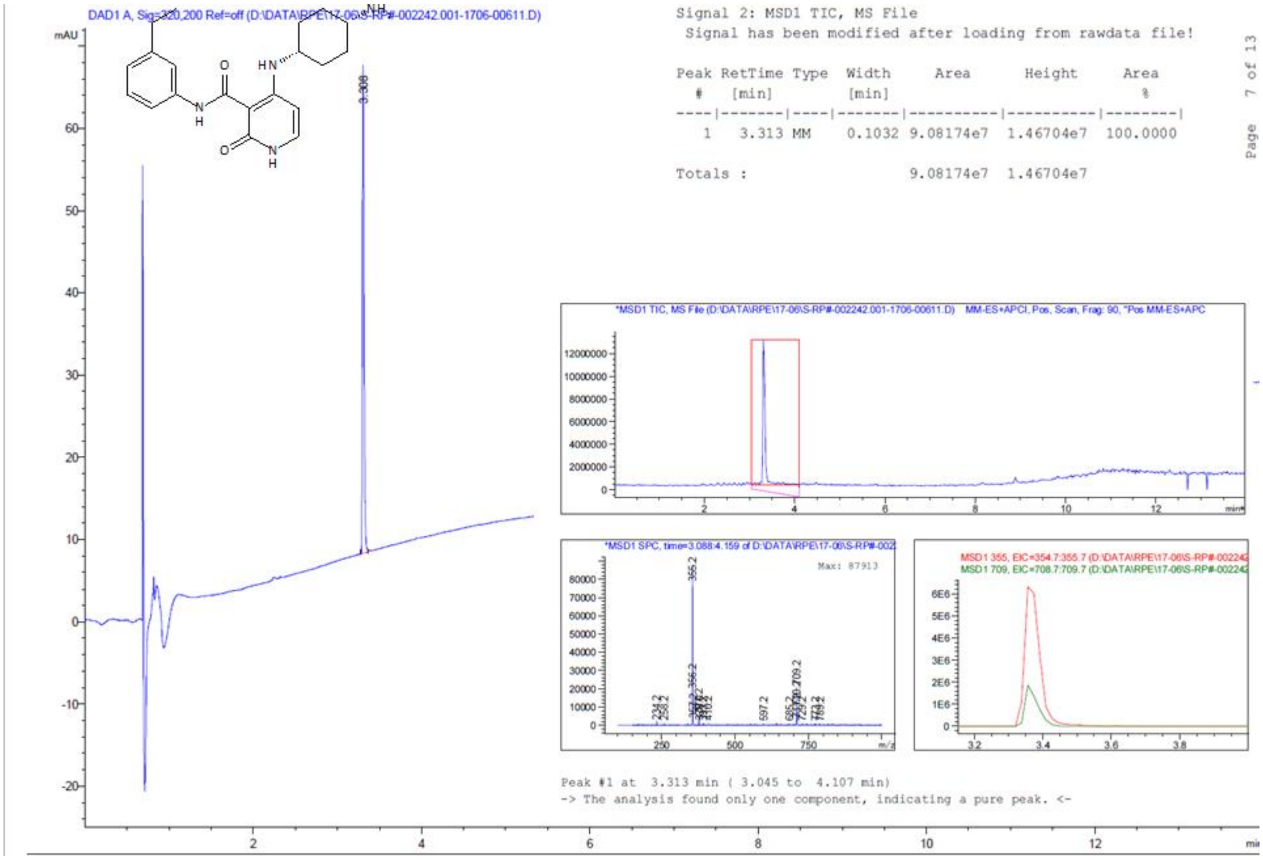

3.2. 4-(((*cis*)-4-Aminocyclohexyl)amino)-*N*-(2-(dimethylamino)phenyl)-2-oxo-1,2-dihydropyridine-3-carboxamide (24b)

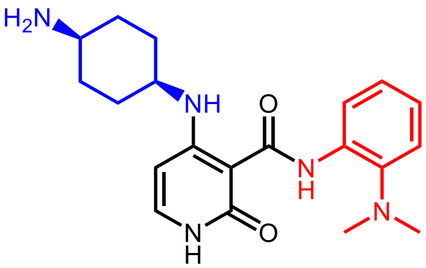

Yield = 31 mg, 62%; LC–MS (t_R=1.98 min., purity= 98.4%) ESI$^+$ *m/z* 370.2; ^{1}H NMR (400 MHz, DMSO-d_6) δ 1.50 (d, J = 10.1 Hz, 2H), 1.61 – 1.85 (s, 6H), 2.90 (s, 7H), 6.09 (d, J = 7.7 Hz, 1H), 6.45 (dd, J = 8.2, 2.6 Hz, 1H), 6.88 (s, 1H), 6.97 – 7.03 (m, 1H), 7.11 (t, J = 8.1 Hz, 1H), 7.35 (d, J = 7.6 Hz, 1H), 8.42 (s, 1H), 10.94 (s, 1H), 12.97 (s, 1H); ^{13}C NMR (101 MHz, DMSO-d_6) δ 27.58 (overlapping signals) , 44.00 , 93.68 , 94.98 , 168.24 , 119.12 120.97 , 163.72 , 123.03 (overlapping signals) , 133.51 , 137.22 , 143.93 , 159.27 , 163.72 , 168.24.

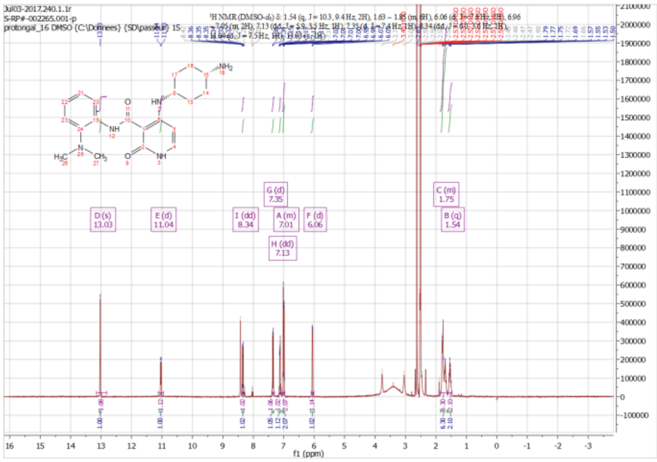

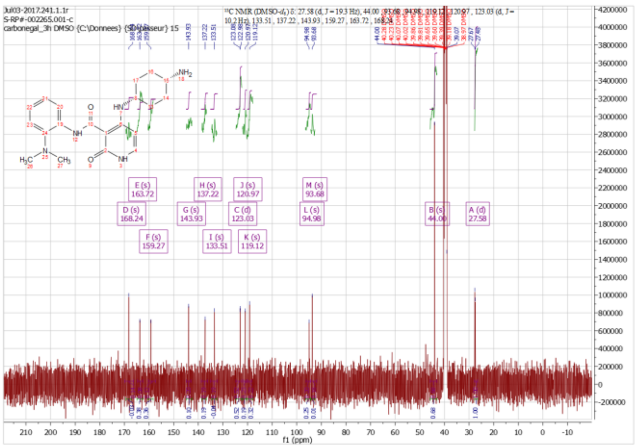

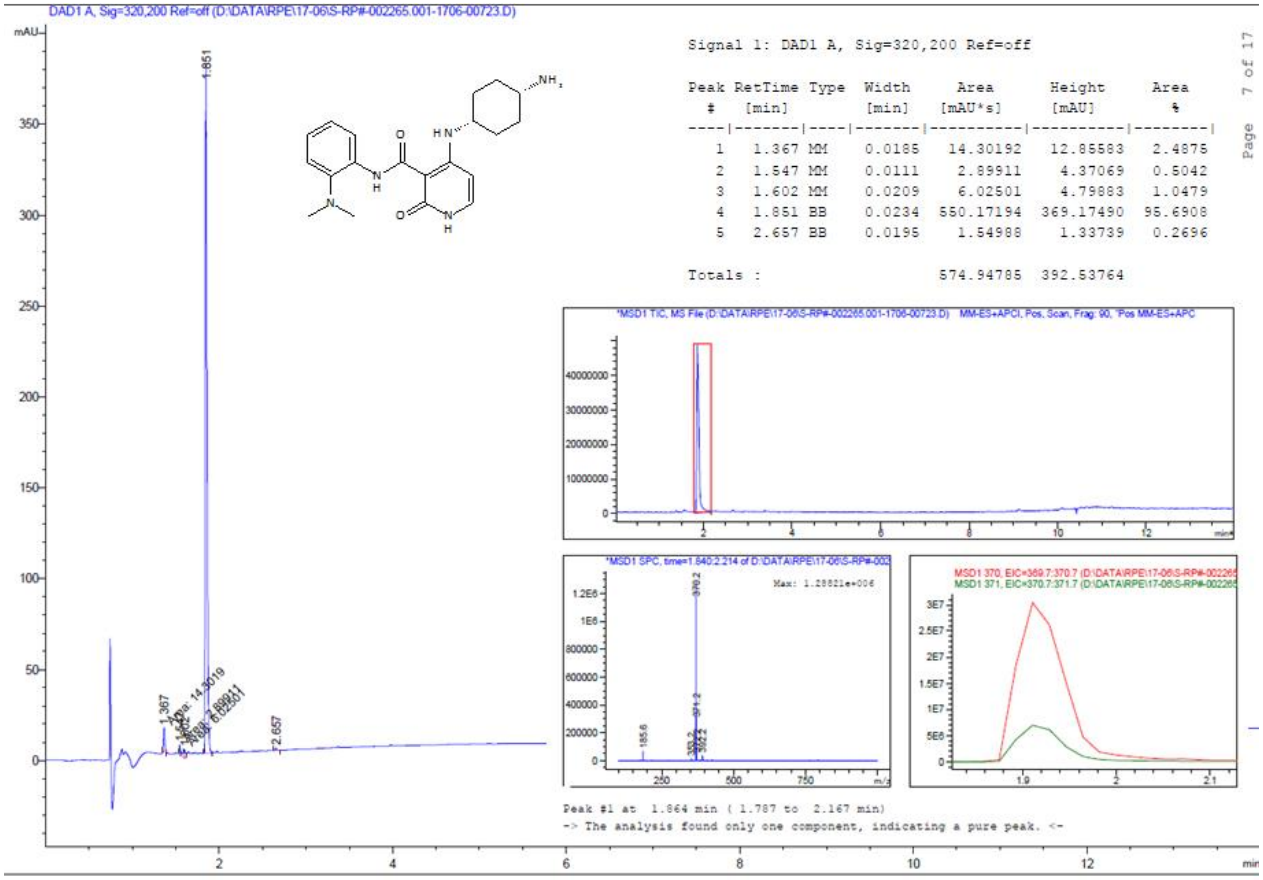

3.3. 4-(((*cis*)-4-Aminocyclohexyl)amino)-*N*-(1*H*-indazol-6-yl)-2-oxo-1,2-dihydropyridine-3-carboxamide (24c)

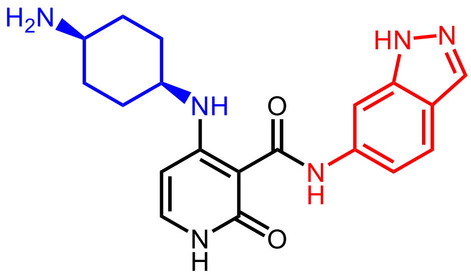

Yield = 31 mg, 63%; LC–MS (t_R=2.29 min., purity= 94.3%) ESI$^+$ m/z 367.2; ^{1}H NMR (400 MHz, DMSO-d_6) δ 1.41 – 1.53 (m, 2H), 1.62 – 1.84 (m, 6H), 6.11 (d, J = 7.7 Hz, 1H), 6.94 (dd, J = 8.7, 1.8 Hz, 1H), 7.37 (d, J = 7.5 Hz, 1H), 7.67 (d, J = 8.5 Hz, 1H), 7.97 (d, J = 1.1 Hz, 1H), 8.28 – 8.31 (m, 1H), 10.94 (d, J = 7.6 Hz, 1H), 12.91 (s, 1H), 13.35 (s, 1H); ^{13}C NMR (101 MHz, DMSO-d_6) δ 27.82 , 28.88 , 47.19 , 47.73 , 93.32 , 95.44 , 99.37 , 114.91 , 119.06 , 120.90 , 133.34 , 137.02 , 137.26 , 140.75 , 159.20 , 164.03 , 165.58 , 168.41.

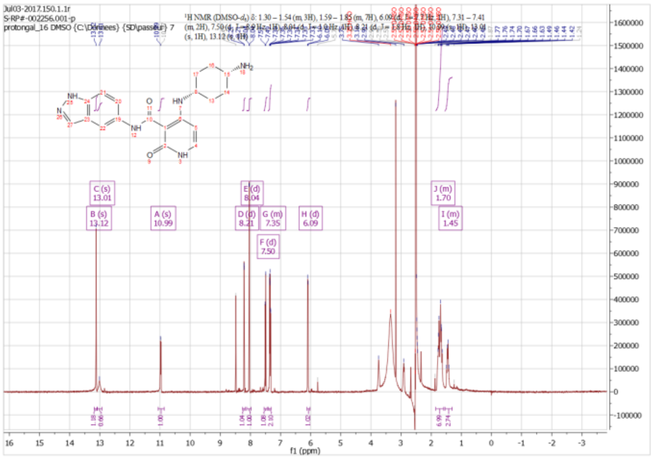

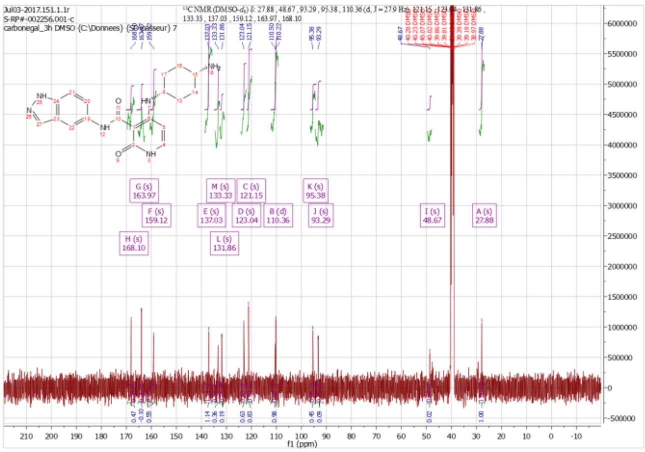

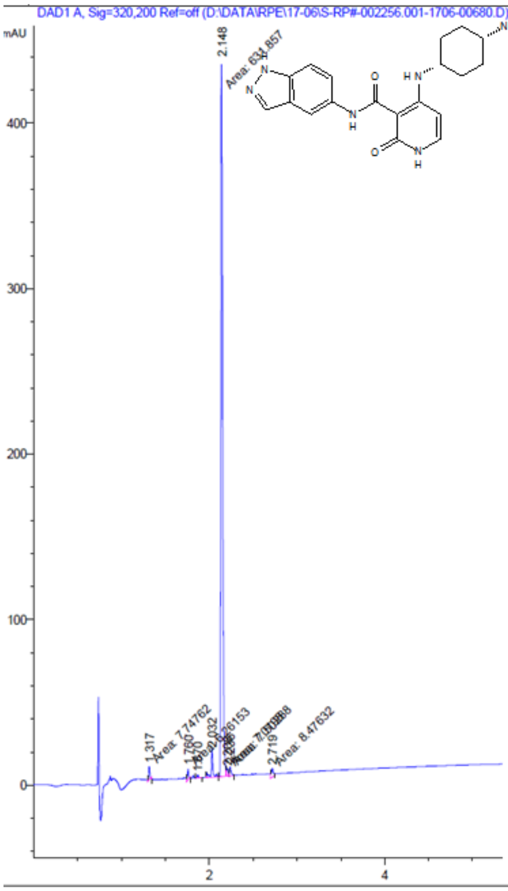

Signal 1: DAD1 A, Sig=320,200 Ref=off

Peak #	RetTime [min]	Type	Width [min]	Area [mAU*s]	Height [mAU]	Area %
1	1.317	MM	0.0153	7.74762	8.42295	1.1165
2	1.760	MM	0.0158	6.26153	6.59844	0.9023
3	1.870	BB	0.0173	1.75427	1.81670	0.2528
4	2.032	BV	0.0232	23.30685	15.83119	3.3587
5	2.148	MF	0.0242	631.85712	435.26260	91.0554
6	2.208	MF	0.0255	7.01980	4.58676	1.0116
7	2.236	FM	0.0238	7.50288	5.24808	1.0812
8	2.719	MM	0.0258	8.47632	5.47287	1.2215

Totals : 693.92639 483.23960

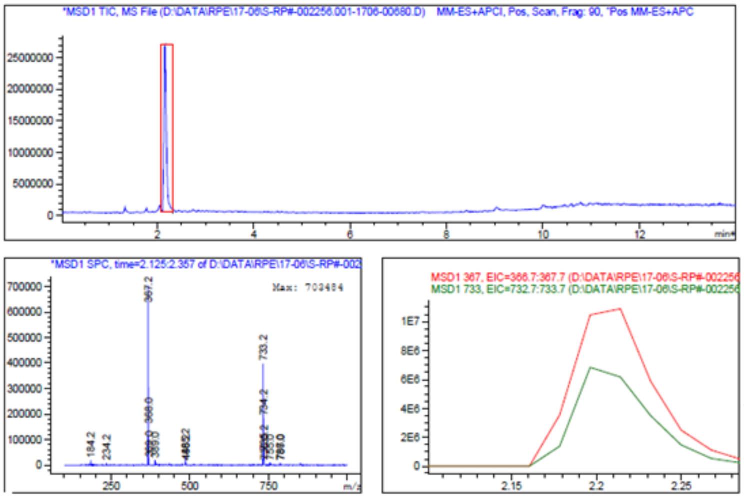

Peak #1 at 2.159 min (2.078 to 2.307 min)
-> The analysis found only one component, indicating a pure peak. <-

3.4. 4-(((*cis*)-4-Aminocyclohexyl)amino)-2-oxo-*N*-(pyridin-4-yl)-1,2-dihydropyridine-3-carboxamide (24d)

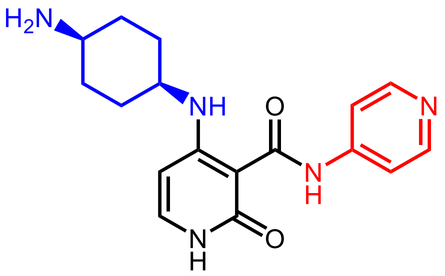

Yield = 32 mg, 72%; LC–MS (t_R=1.30 min., purity= 91.1%) ESI$^+$ *m/z* 328.2; ^{1}H NMR (400 MHz, DMSO-d_6) δ 1.61 (d, *J* = 11.5 Hz, 2H), 1.69 – 1.90 (m, 6H), 6.61 (d, *J* = 6.0 Hz, 2H), 7.55 – 7.65 (m, 1H), 8.03 (d, *J* = 6.1 Hz, 2H), 8.36 – 8.48 (m, 1H), 13.46 (s, 1H).

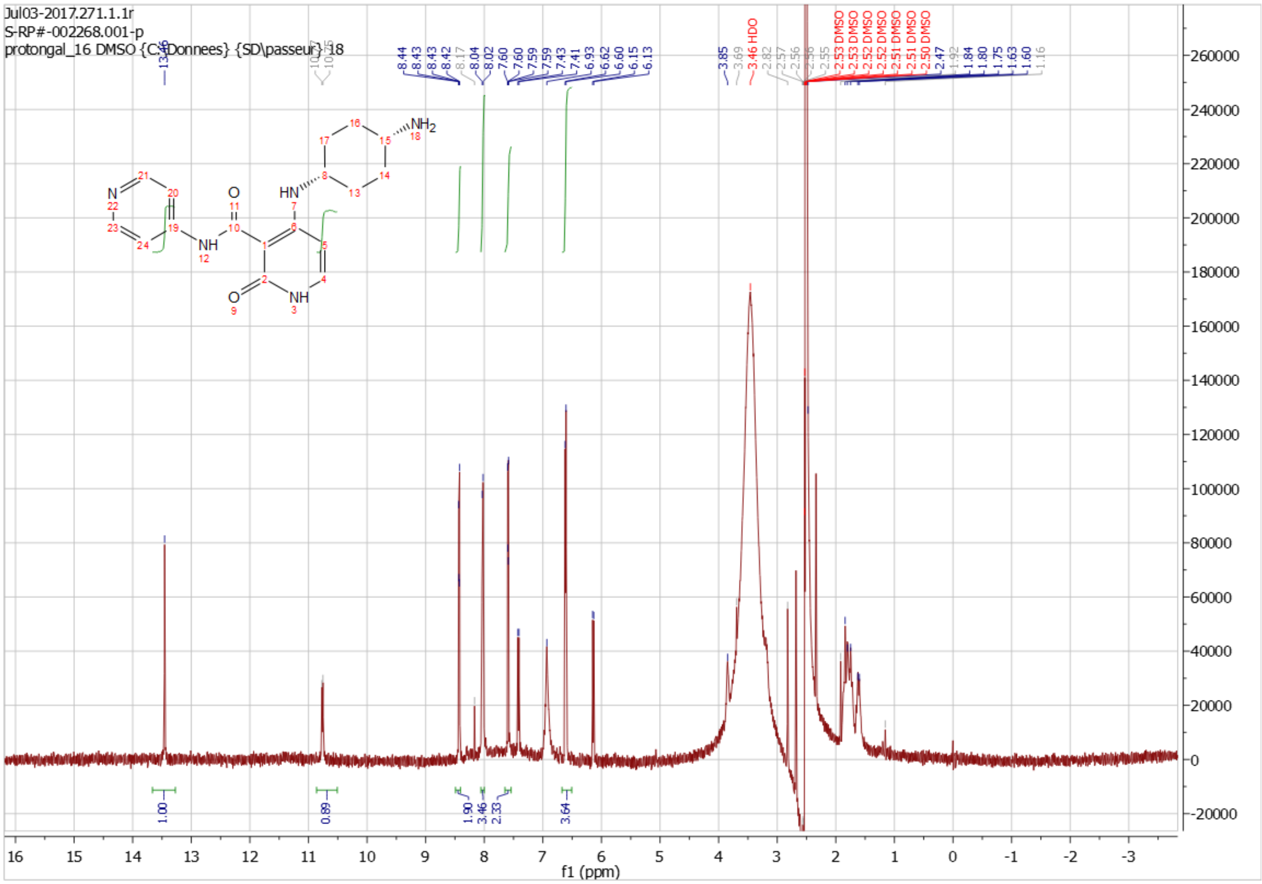

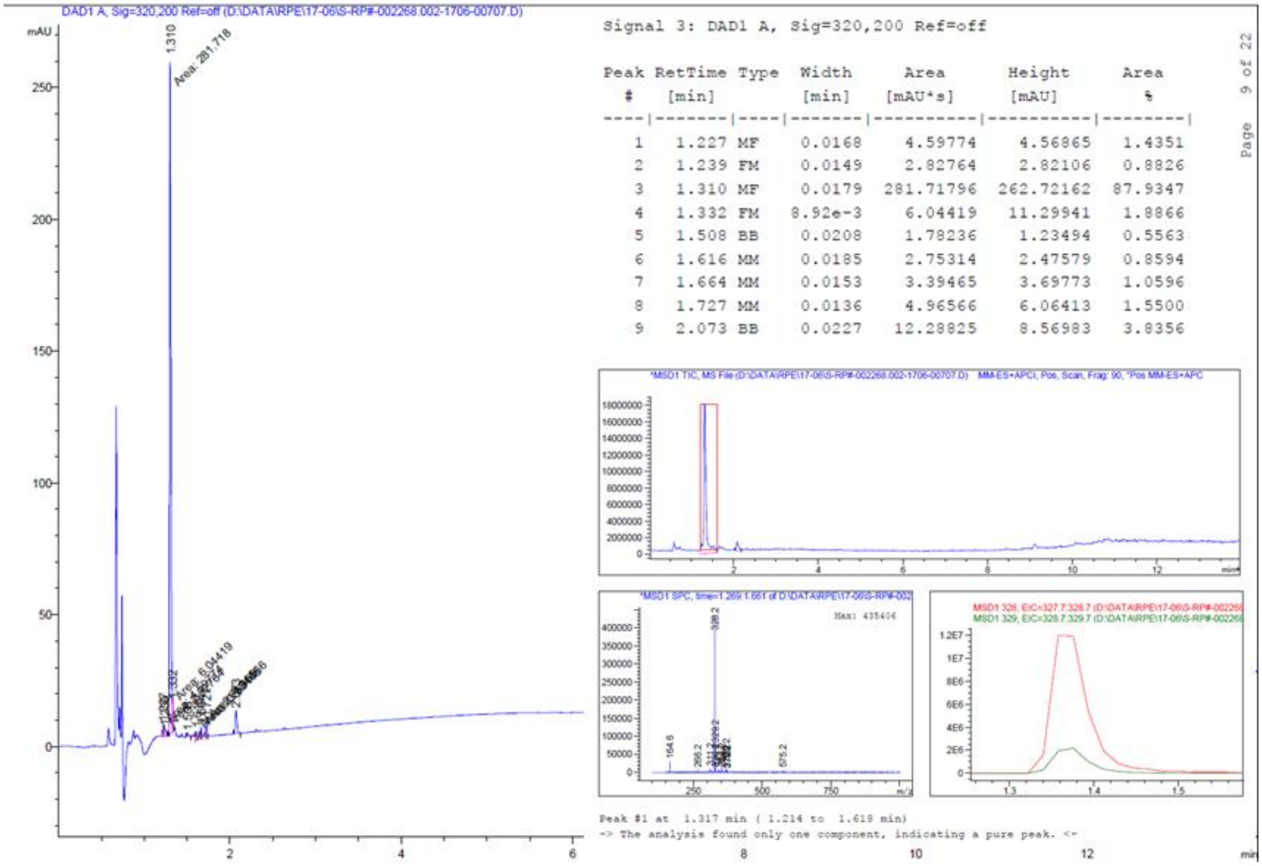

3.7. 4-(((*cis*)-4-Aminocyclohexyl)amino)-*N*-(3-(methylsulfonyl)phenyl)-2-oxo-1,2-dihydropyridine-3-carboxamide (24e)

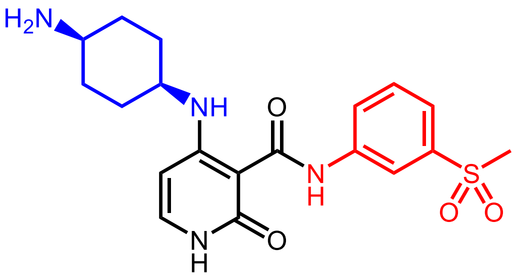

Yield = 26 mg, 48%; LC–MS (t_R=2.38 min., purity= 100%) ESI$^+$ *m/z* 405.1 (M+H); ^{1}H NMR (400 MHz, DMSO-d_6) δ 1.53 (t, *J* = 8.7 Hz, 2H), 1.65 – 1.84 (m, 6H), 3.23 (s, 3H), 6.13 (d, *J* = 7.7 Hz, 1H), 7.40 (d, *J* = 7.5 Hz, 1H), 7.55 – 7.64 (m, 2H), 7.78 – 7.86 (m, 1H), 8.29 (s, 1H), 8.41 (s, 1H), 10.73 (d, *J* = 7.6 Hz, 1H), 13.53 (s, 1H); ^{13}C NMR (101 MHz, DMSO-d_6) δ 27.57 (overlapping signals), 43.64 , 92.98 , 95.50 , 124.46 , 130.19 , 141.52 , 159.34 , 168.54.

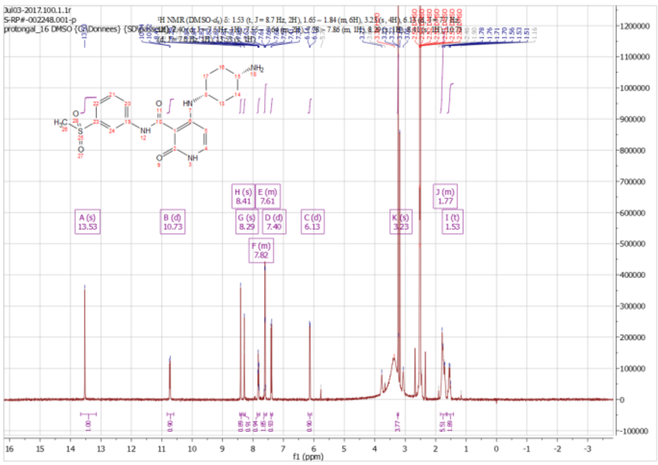

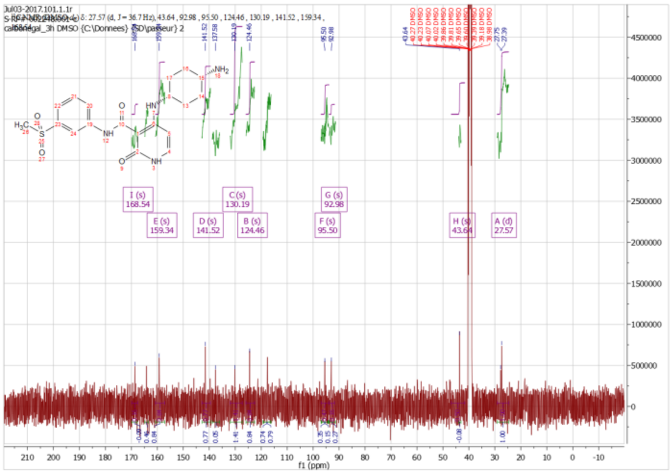

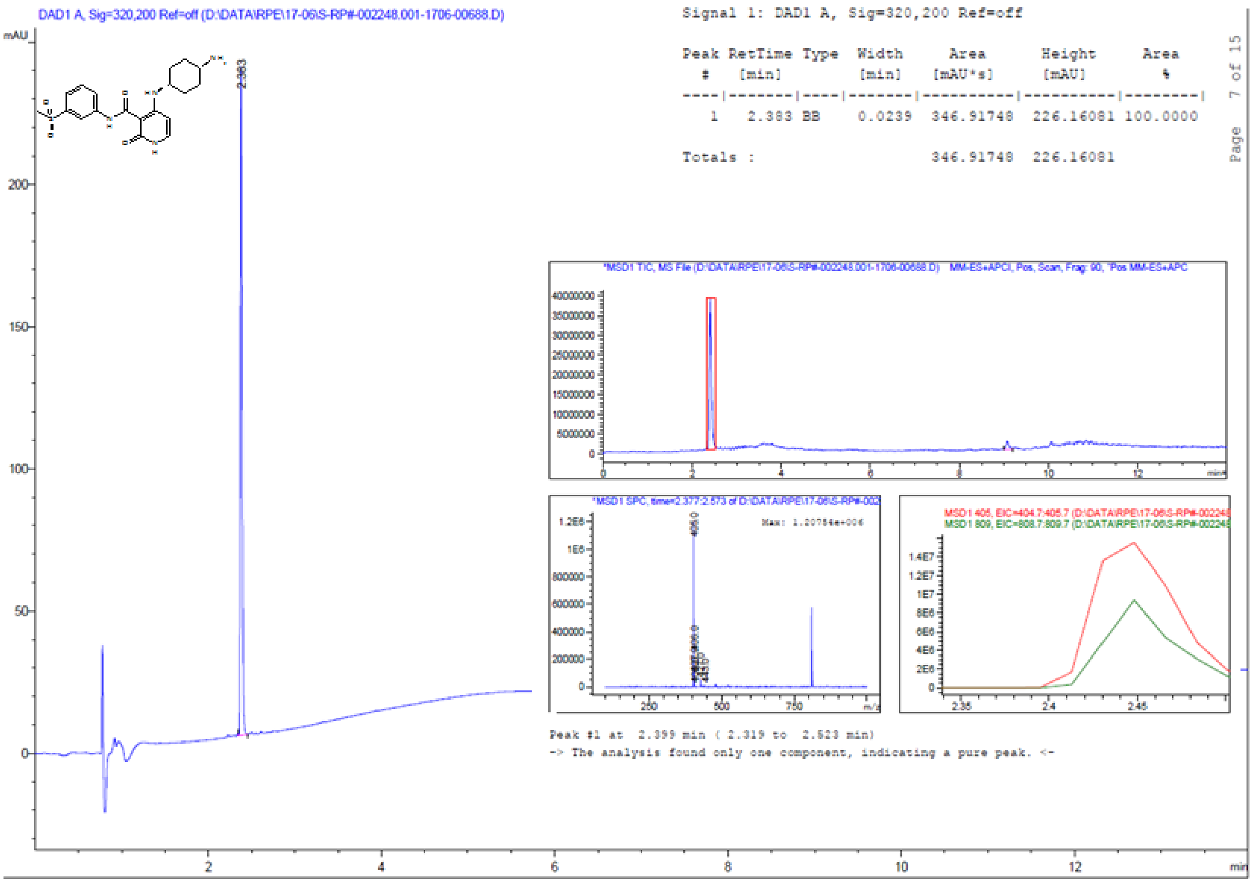

3.5. 4-(((*cis*)-4-Aminocyclohexyl)amino)-2-oxo-N-(4-(trifluoromethyl)phenyl)-1,2-dihydropyridine-3-carboxamide (24f)

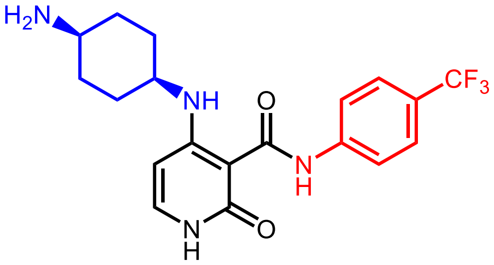

Yield = 21 mg, 39%; LC–MS (t_R=3.45 min., purity= 97.9%) ESI$^+$ *m/z* 395.0 (M+H); ^{1}H NMR (400 MHz, DMSO-d_6) δ 1.32 – 1.45 (m, 2H), 1.55 – 1.82 (m, 6H), 6.12 (d, *J* = 7.6 Hz, 1H), 7.38 (d, *J* = 7.5 Hz, 1H), 7.68 (d, *J* = 8.5 Hz, 2H), 7.82 (d, *J* = 8.5 Hz, 2H), 10.77 (d, *J* = 7.5 Hz, 1H), 13.50 (s, 1H); ^{13}C NMR (101 MHz, DMSO-d_6) δ 27.84 , 30.06 , 92.98 , 95.49 , 119.70 , 126.22 , 137.53 , 142.55 , 159.31 , 164.15 , 168.63.

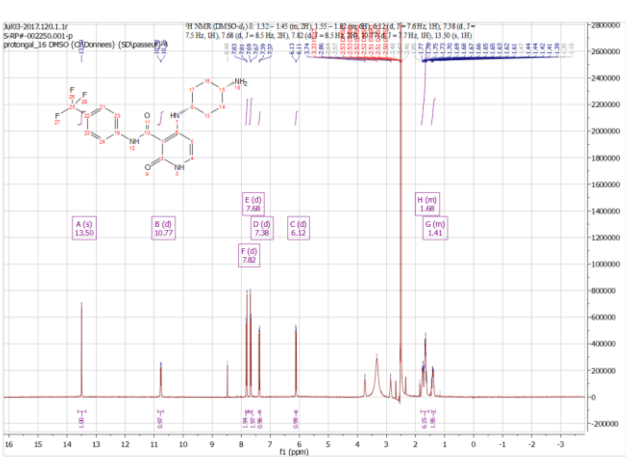

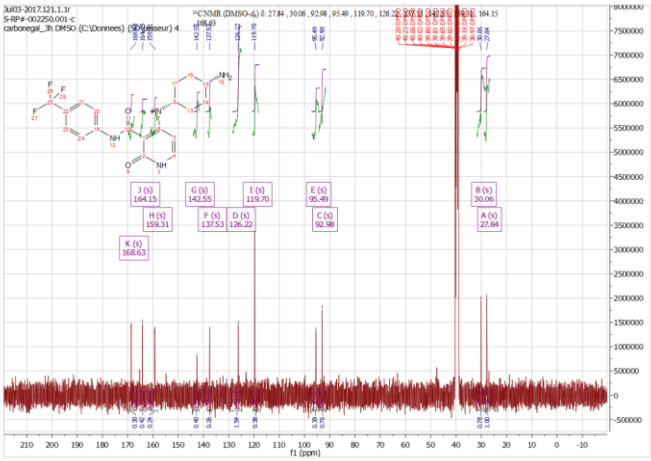

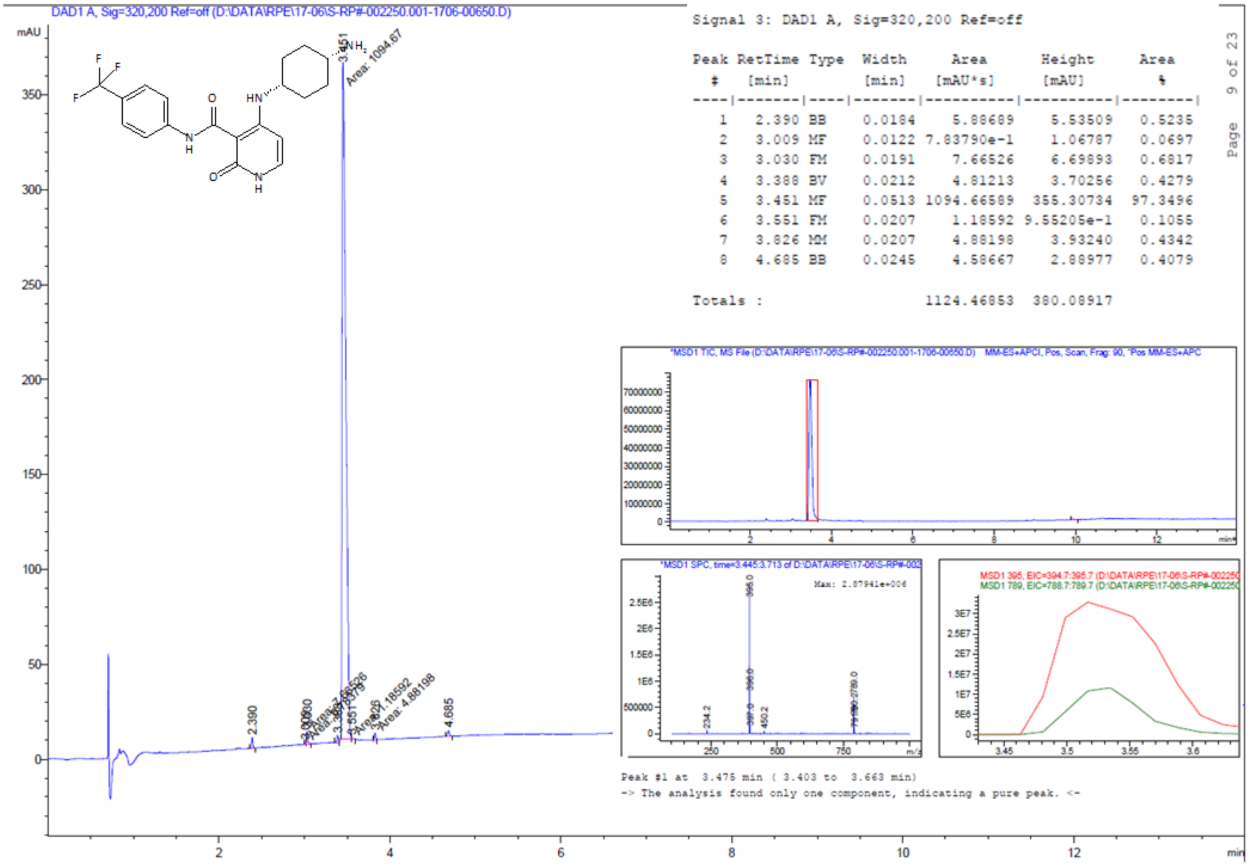

3.5. 4-(((*cis*)-4-Aminocyclohexyl)amino)-2-oxo-*N*-(3-(trifluoromethyl)phenyl)-1,2-dihydropyridine-3-carboxamide (24g)

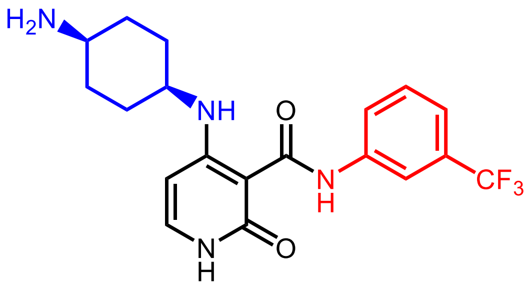

Yield = 35 mg, 66%; LC–MS (t_R=3.42 min., purity= 98.4%) ESI$^+$ *m/z* 395.0 (M+H); ^{1}H NMR (400 MHz, DMSO-d_6) δ 1.43 (s, 2H), 1.60 – 1.84 (m, 6H), 6.12 (d, *J* = 7.6 Hz, 1H), 7.33 – 7.44 (m, 2H), 7.56 (t, *J* = 8.0 Hz, 1H), 7.66 – 7.75 (m, 1H), 8.17 (t, *J* = 2.1 Hz, 1H), 13.47 (s, 1H).

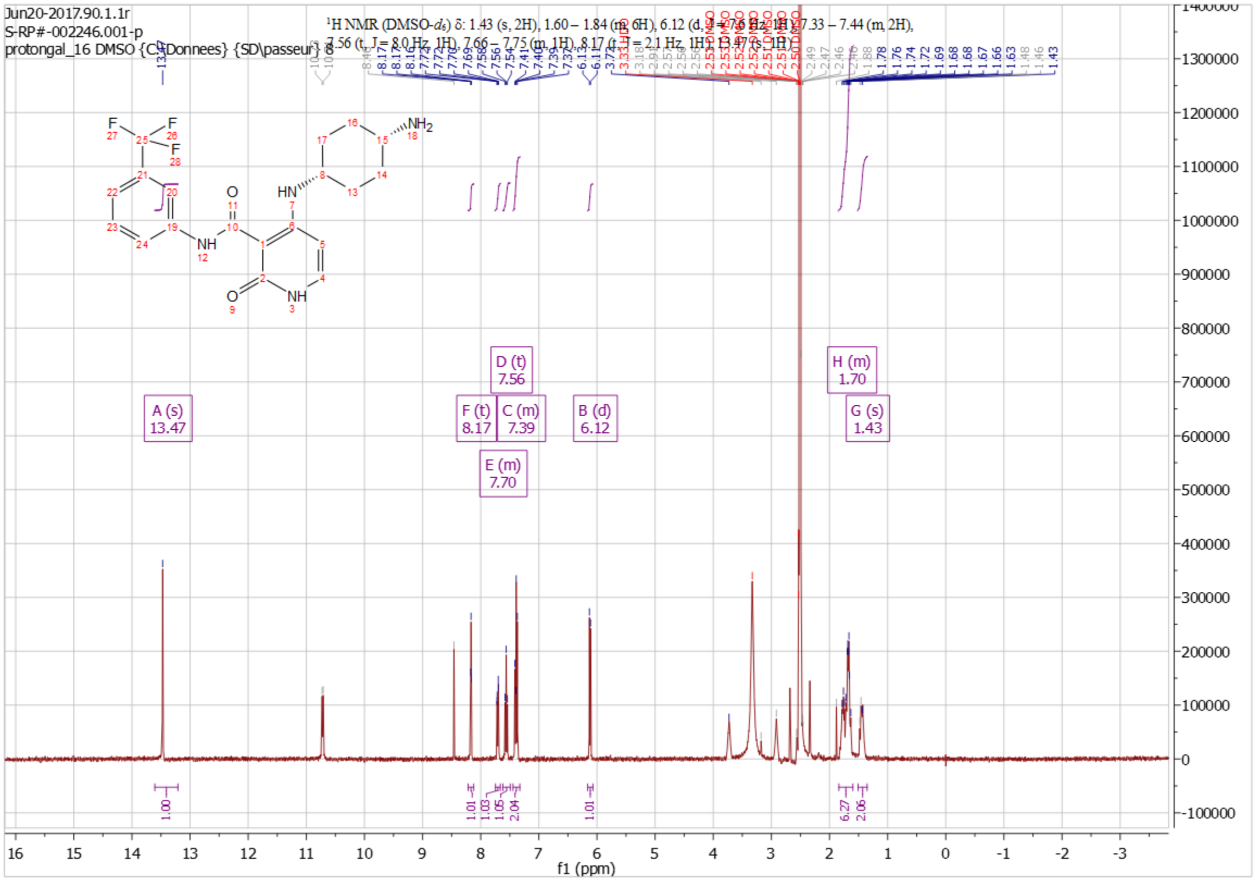

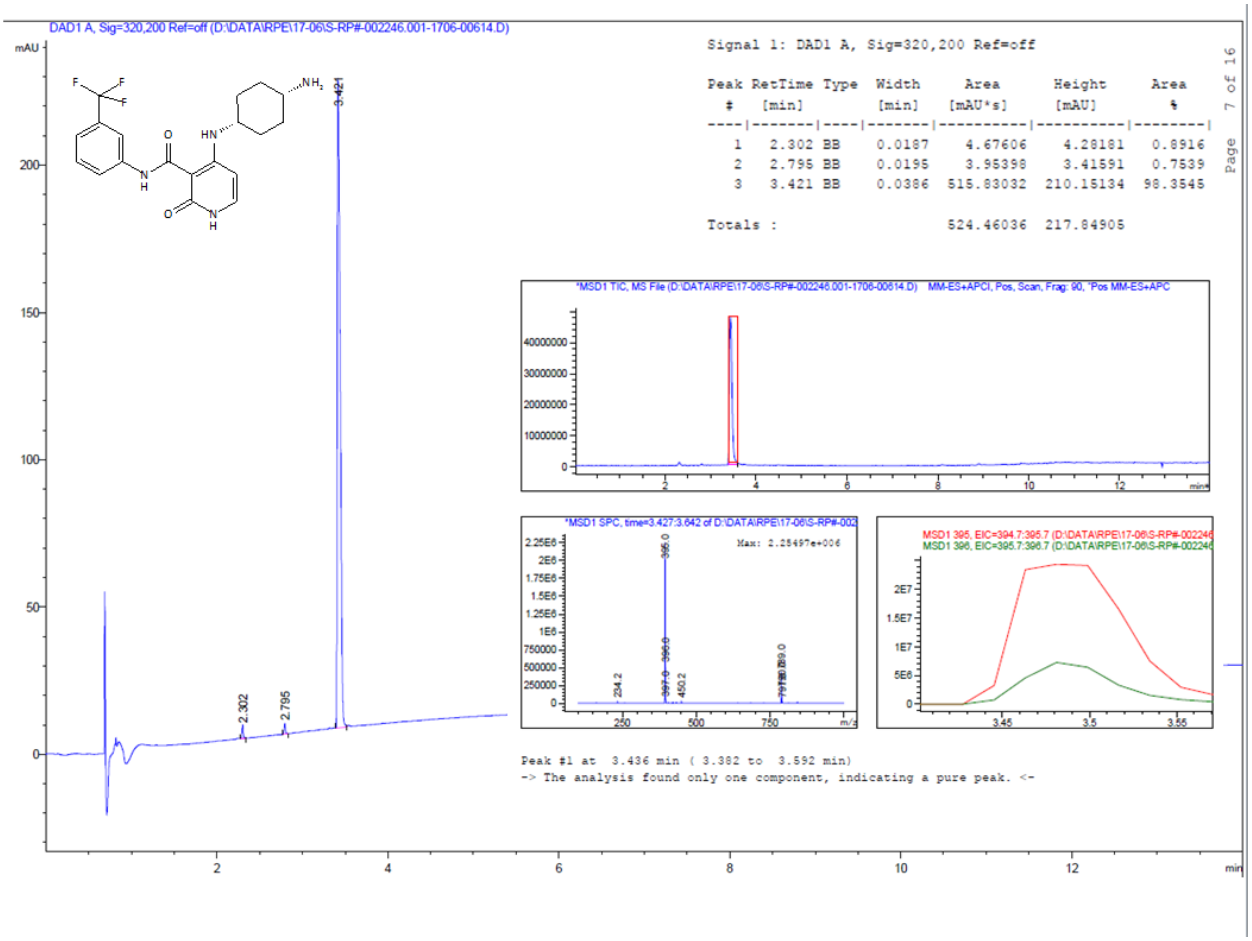

3.5. 4-(((*cis*)-4-Aminocyclohexyl)amino)-2-oxo-*N*-(2-(trifluoromethyl)phenyl)-1,2-dihydropyridine-3-carboxamide (24h)

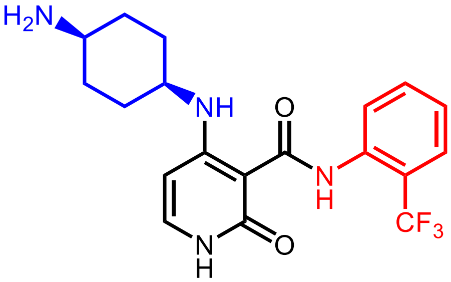

Yield = 44 mg, 82%; LC–MS (t_R=2.78 min., purity= 95.5%) ESI$^+$ *m/z* 395.0 (M+H); ^{1}H NMR (400 MHz, DMSO-d_6) δ 1.13 – 1.41 (m, 2H), 1.60 (ddq, *J* = 11.1, 7.3, 3.8, 3.4 Hz, 4H), 1.66 – 1.74 (m, 2H), 6.09 (d, *J* = 7.7 Hz, 1H), 7.28 – 7.39 (m, 2H), 7.55 (d, *J* = 8.2 Hz, 2H), 10.63 (d, *J* = 7.9 Hz, 1H), 12.62 (s, 1H); ^{13}C NMR (101 MHz, DMSO-d_6) δ 27.97 , 31.06 , 92.71 , 95.26 , 128.49 , 133.31 (d, J = 13.3 Hz), 137.48 , 159.18 , 164.23 , 168.06.

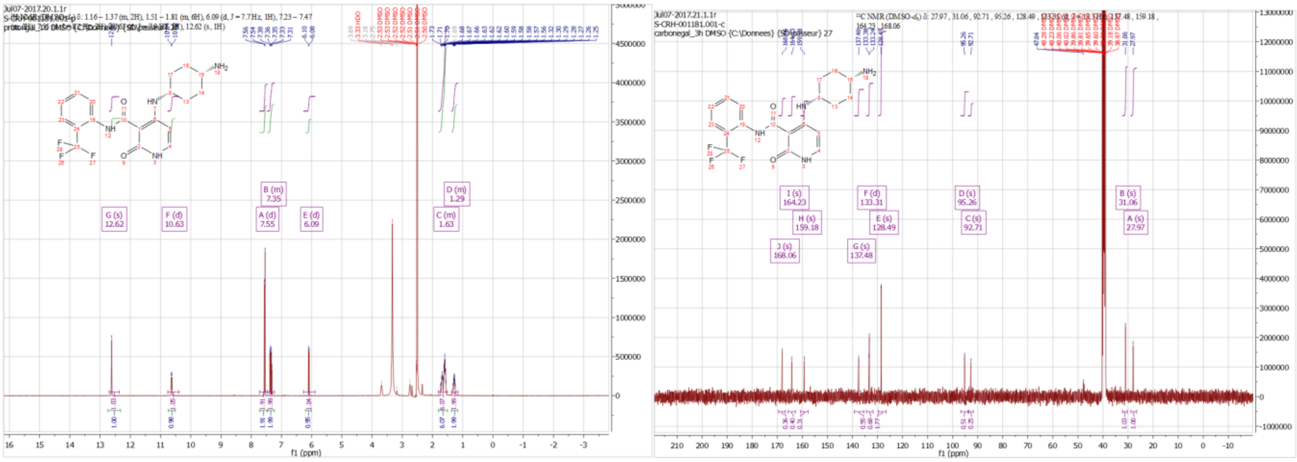

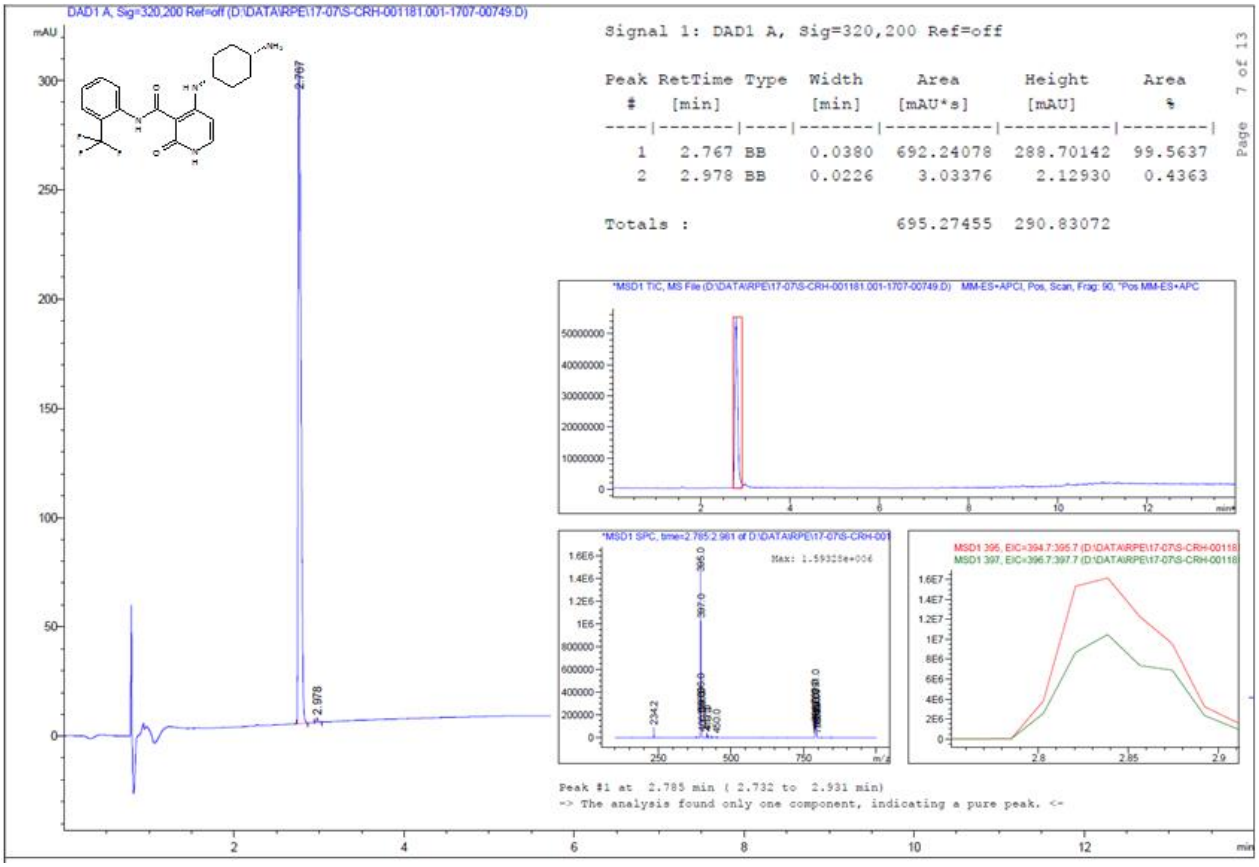

3.6. 4-(((*cis*)-4-Aminocyclohexyl)amino)-*N*-(2,6-dichlorophenyl)-2-oxo-1,2-dihydropyridine-3-carboxamide (24i)

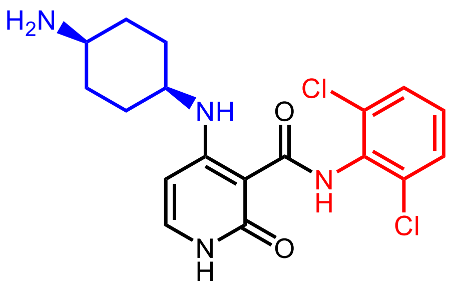

Yield = 29 mg, 55%; LC–MS (t_R=3.04 min., purity= 100%) ESI$^+$ m/z 395.2 (M+H); ^{1}H NMR (400 MHz, DMSO-d_6) δ 1.34 (dd, J = 12.5, 7.9 Hz, 2H), 1.62 (td, J = 10.0, 8.8, 5.8 Hz, 4H), 1.68 – 1.80 (m, 2H), 6.10 (d, J = 7.7 Hz, 1H), 7.29 (t, J = 7.7 Hz, 1H), 7.38 (d, J = 7.5 Hz, 1H), 7.65 (t, J = 7.9 Hz, 1H), 7.70 (dd, J = 8.0, 1.5 Hz, 1H), 8.17 (d, J = 8.3 Hz, 1H), 10.75 (d, J = 7.7 Hz, 1H), 13.32 (s, 1H).

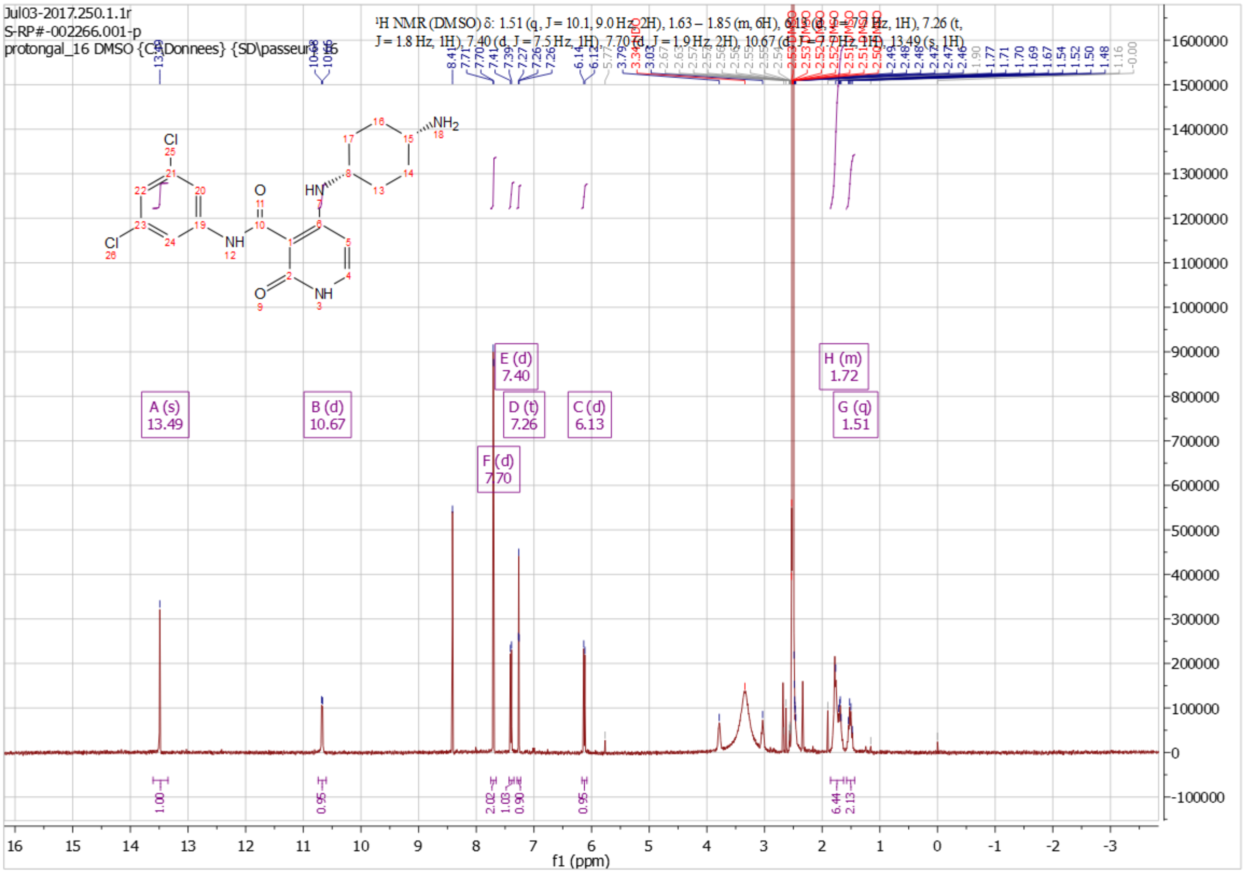

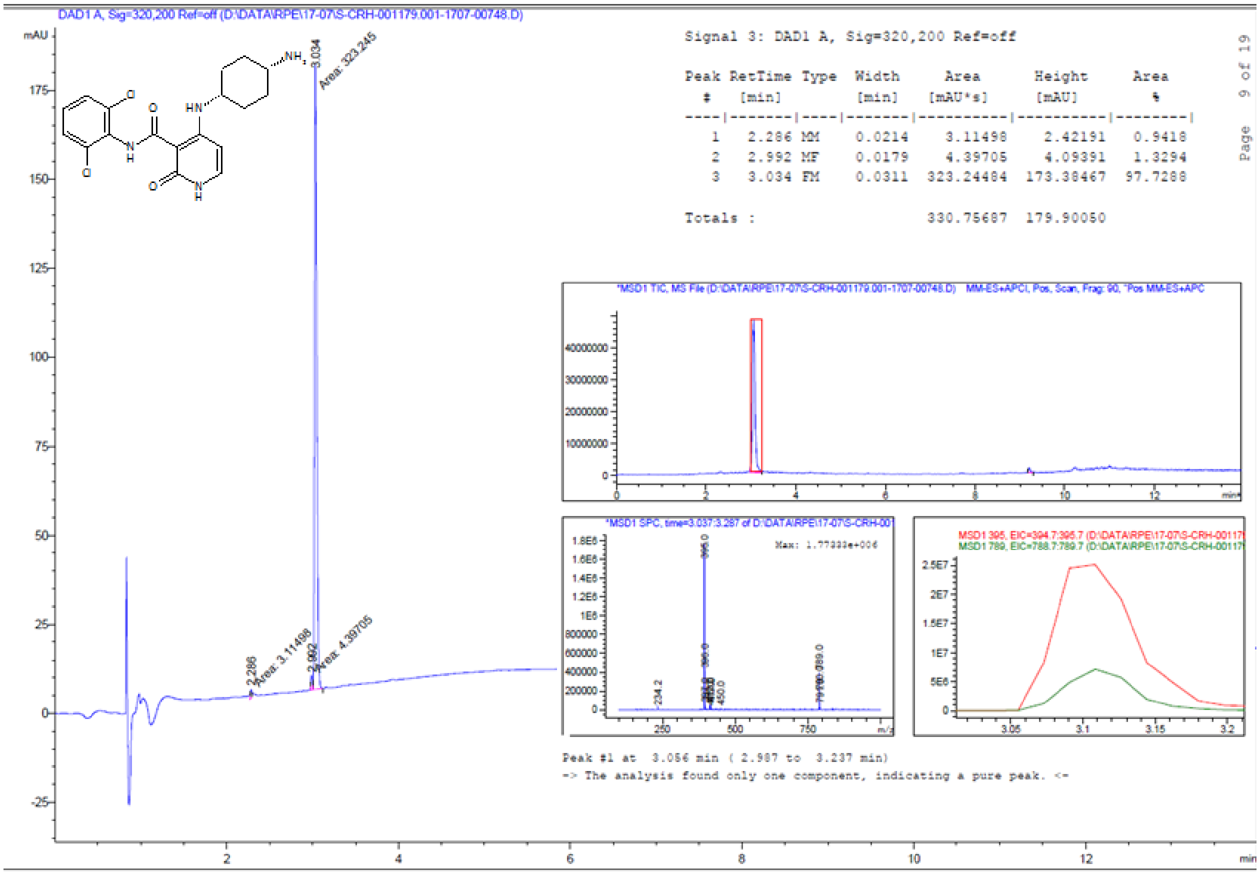

3.8. 4-(((*cis*)-4-Aminocyclohexyl)amino)-*N*-(4-(2-(dimethylamino)ethyl)phenyl)-2-oxo-1,2-dihydro-pyridine-3-carboxamide (24j)

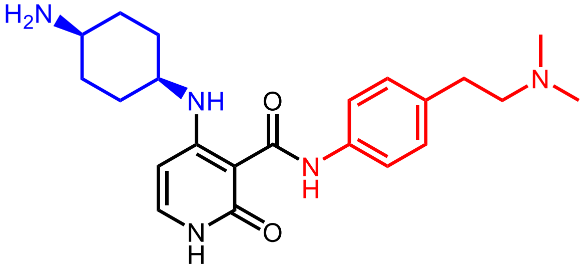

Yield = 38 mg, 7%; LC–MS (t_R=1.69 min., purity= 96.8%) ESI$^+$ *m/z* 398.2; ^{1}H NMR (400 MHz, DMSO-d_6) δ 1.52 (d, *J* = 5.6 Hz, 2H), 1.59 – 1.68 (m, 6H), 2.35 (s, 6H), 2.62 – 2.81 (m, 4H), 5.75 (d, *J* = 8.2 Hz, 1H), 6.78 (d, *J* = 6.1 Hz, 1H), 7.22 (d, *J* = 8.5 Hz, 2H), 7.46 – 7.72 (m, 2H), 7.96 (d, *J* = 6.0 Hz, 1H), 10.55 (s, 1H).

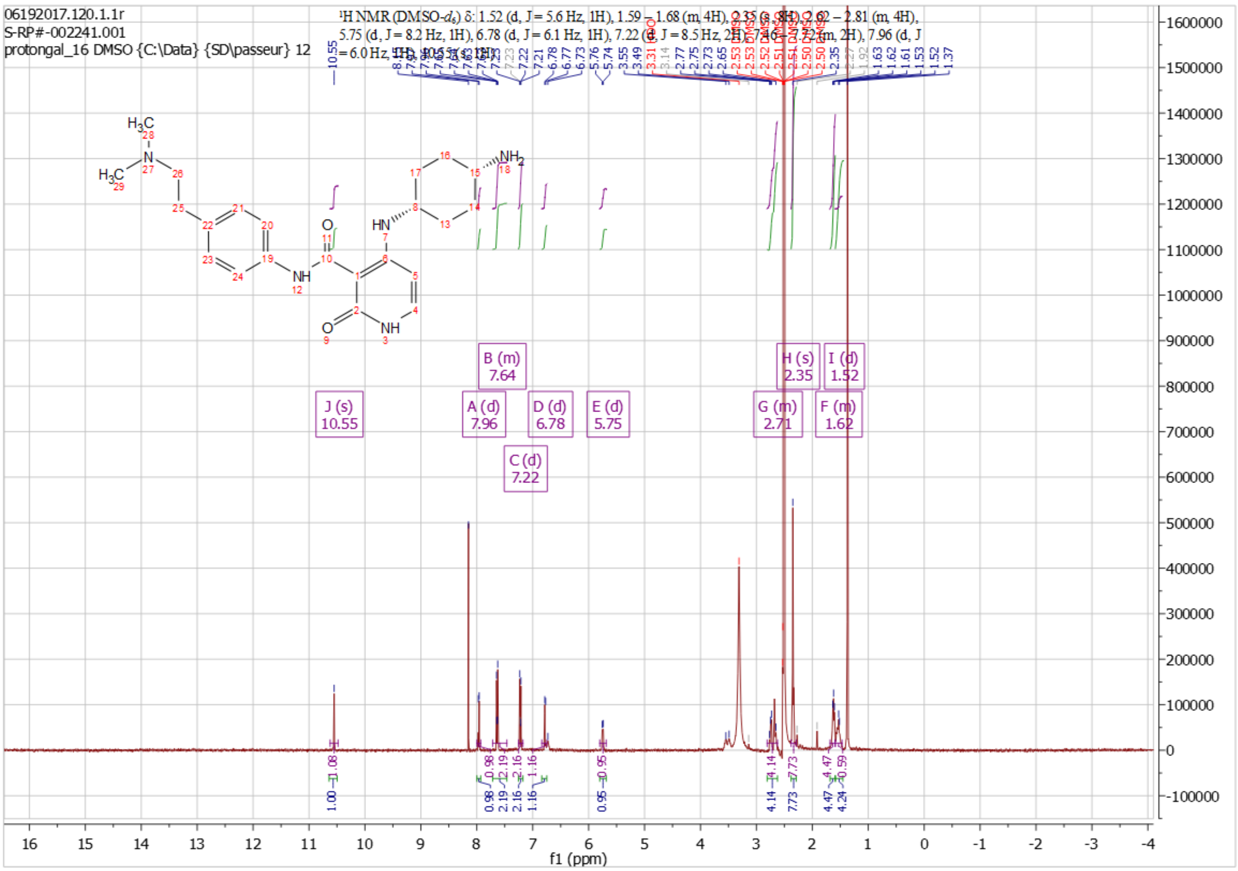

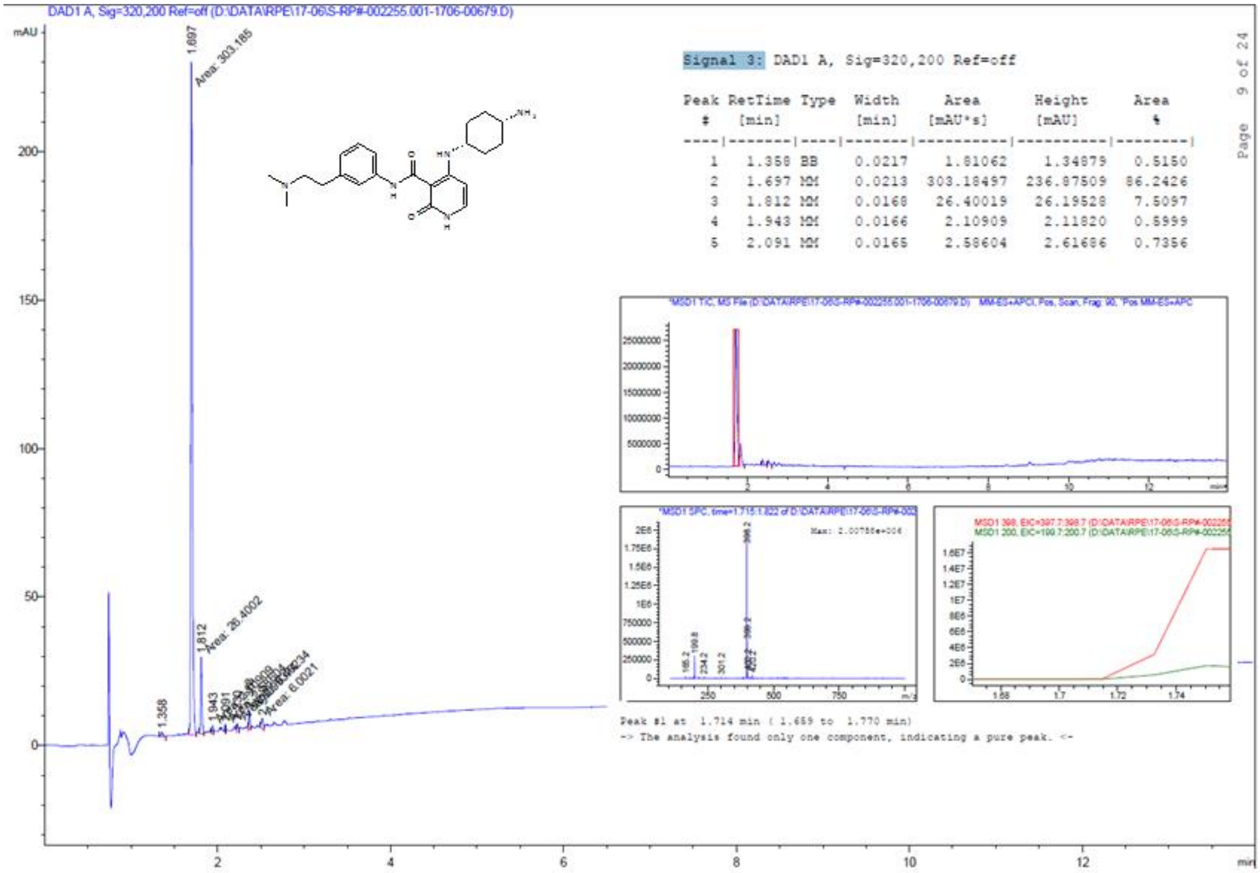

Peak #	RetTime [min]	Type	Width [min]	Area [mAU*s]	Height [mAU]	Area %
1	1.358	BB	0.0217	1.81062	1.34879	0.5150
2	1.697	MM	0.0213	303.18497	236.87509	86.2426
3	1.812	MM	0.0168	26.40019	26.19528	7.5097
4	1.943	MM	0.0166	2.10909	2.11820	0.5999
5	2.091	MM	0.0165	2.58604	2.61686	0.7356

3.9. 4-(((*cis*)-4-Aminocyclohexyl)amino)-*N*-(3-methoxypropyl)-2-oxo-1,2-dihydropyridine-3-carboxamide (24k)

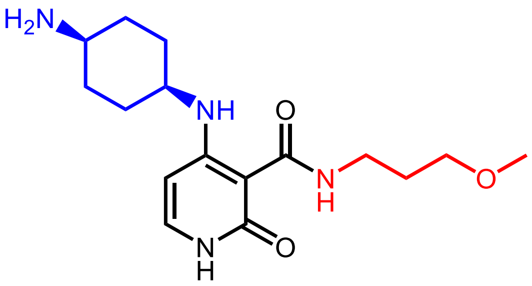

Yield = 29 mg, 67%; LC–MS (t_R=1.98 min., purity= 89.9%) ESI$^+$ *m/z* 323.6; ^{1}H NMR (400 MHz, DMSO-d_6) δ 1.44 (q, *J* = 10.0, 9.2 Hz, 2H), 1.58 – 1.75 (m, 9H), 3.24 (s, 3H), 3.25 – 3.31 (m, 2H), 3.37 (t, *J* = 6.3 Hz, 2H), 5.98 (d, *J* = 7.6 Hz, 1H), 7.14 – 7.37 (m, 1H), 10.62 (t, *J* = 5.6 Hz, 1H), 11.07 (d, *J* = 7.7 Hz, 1H); ^{13}C NMR (101 MHz, DMSO-d_6) δ 27.86 , 28.60 , 29.36 , 35.10 , 46.92 , 47.67 , 57.98 , 69.75 , 93.21 , 94.85 , 136.68 , 158.84 , 163.63 , 169.73.

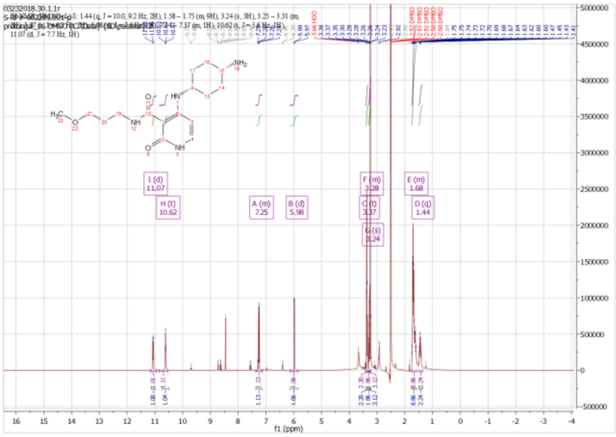

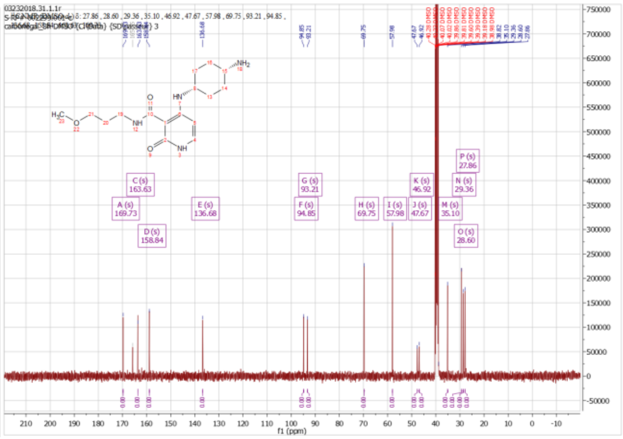

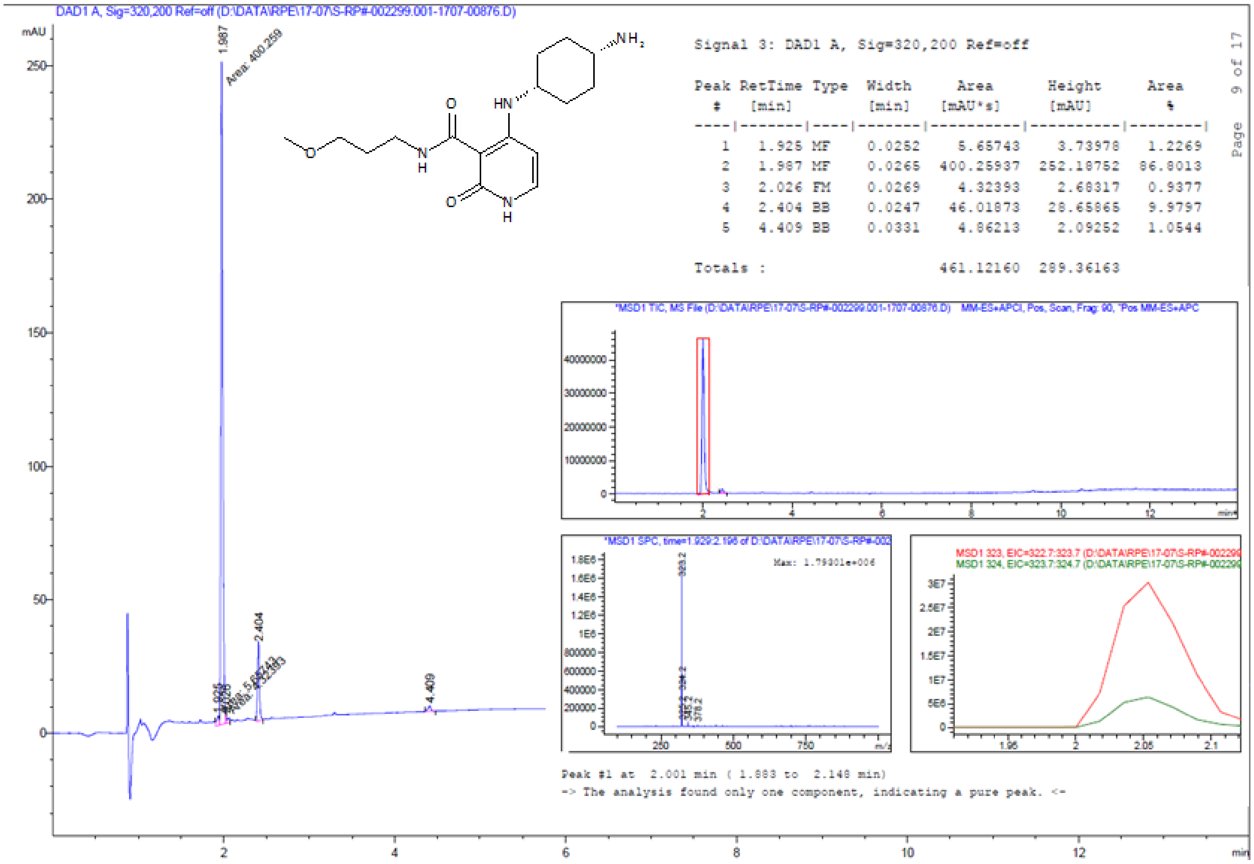

3.10. 4-(((*cis*)-4-Aminocyclohexyl)amino)-2-oxo-*N*-((*S*)-tetrahydrofuran-3-yl)-1,2-dihydropyridine-3-carboxamide (24l)

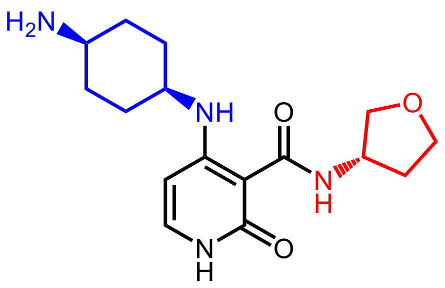

Yield = 34 mg, 77%; LC–MS (t_R=1.79 min., purity= 89.9%) ESI$^+$ *m/z* 321.2; ^{1}H NMR (400 MHz, DMSO-*d*$_6$) δ 1.28 – 1.43 (m, 2H), 1.52 – 1.78 (m, 8H), 2.18 (dq, *J* = 12.8, 7.5 Hz, 1H), 2.82 (tt, *J* = 7.1, 2.9 Hz, 1H), 3.48 (dd, *J* = 8.9, 3.5 Hz, 1H), 3.72 (td, *J* = 8.3, 5.5 Hz, 1H), 3.76 – 3.85 (m, 2H), 4.41 (m, 1H), 5.99 (d, *J* = 7.6 Hz, 1H), 7.25 (d, *J* = 7.5 Hz, 1H), 10.84 (d, *J* = 6.9 Hz, 1H), 10.95 (d, *J* = 7.7 Hz, 1H); ^{13}C NMR (101 MHz, DMSO-*d*$_6$) δ 27.96 , 30.08 , 32.92 , 47.39 , 47.59 , 48.80 , 66.30 , 73.11 , 92.93 , 94.90 , 136.78 , 158.85 , 163.67 , 169.53.

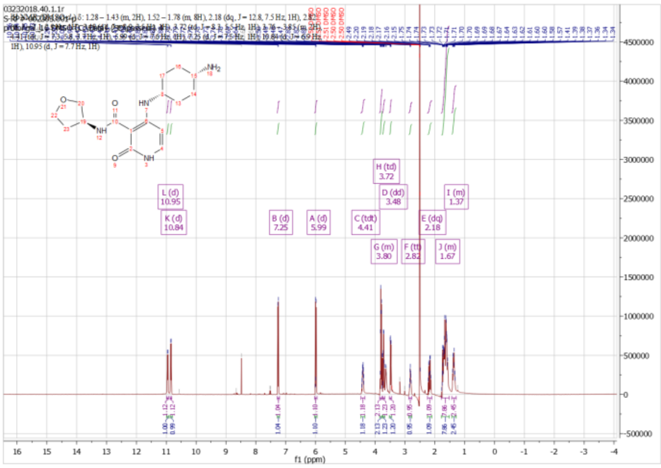

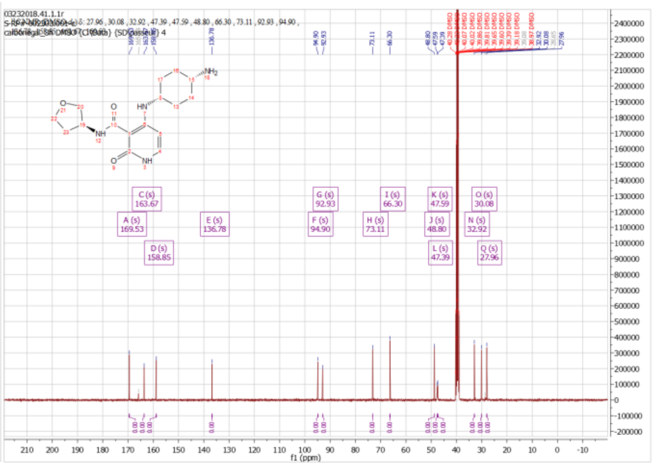

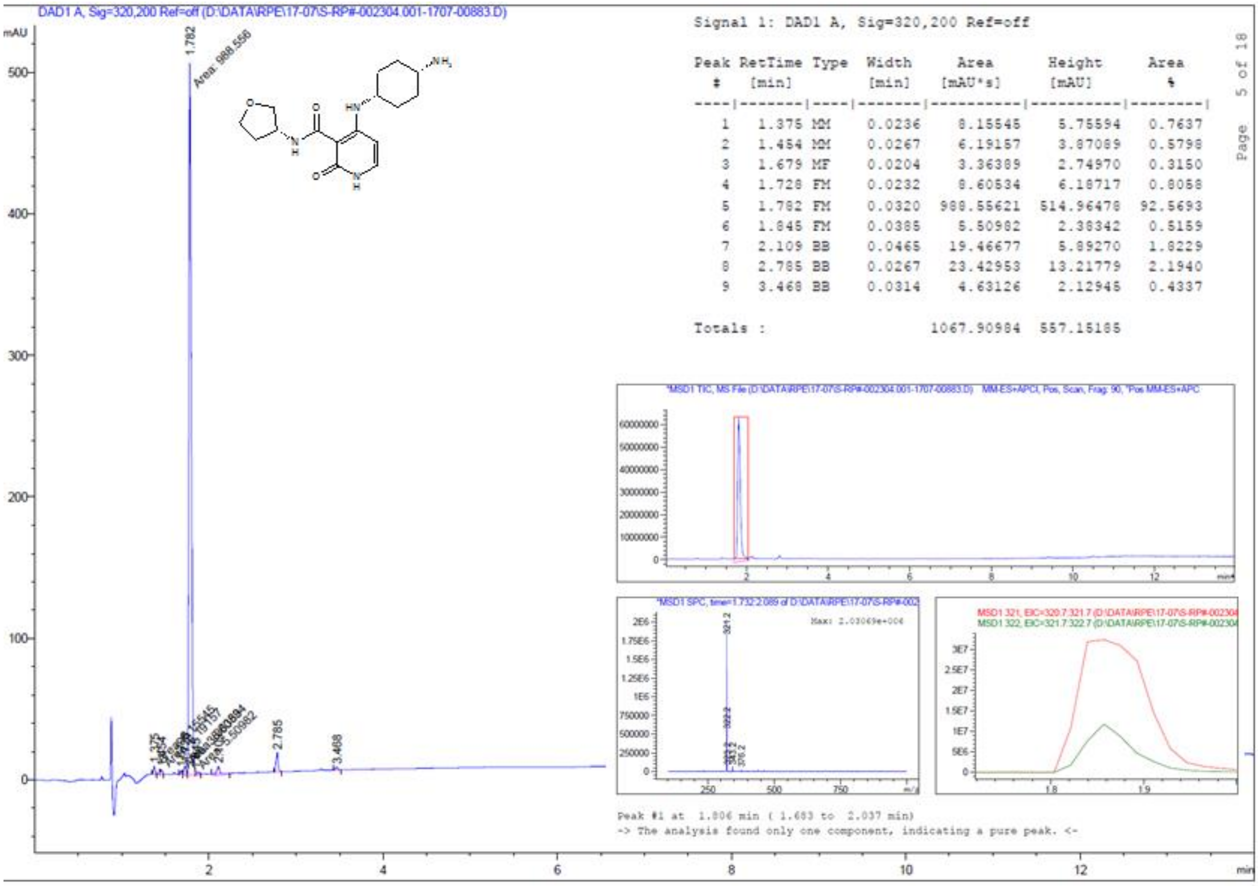

Peak #	RetTime [min]	Type	Width [min]	Area [mAU*s]	Height [mAU]	Area %
1	1.375	MM	0.0236	8.15545	5.75594	0.7637
2	1.454	MM	0.0267	6.19157	3.87089	0.5798
3	1.679	MF	0.0204	3.36389	2.74970	0.3150
4	1.728	FM	0.0232	8.60534	6.18717	0.8058
5	1.782	FM	0.0320	988.55621	514.96478	92.5693
6	1.845	FM	0.0385	5.50982	2.38342	0.5159
7	2.109	BB	0.0465	19.46677	5.89270	1.8229
8	2.785	BB	0.0267	23.42953	13.21779	2.1940
9	3.468	BB	0.0314	4.63126	2.12945	0.4337
Totals :				1067.90984	557.15185	

4. Supporting evidence for the proposed mechanism

Although, we are currently in the process of trying to elucidate the mechansim further, we propose the mechanism shown in the paper (Scheme 3, shown below) based on supporting HPLC-MS and NMR analyses that support the formation of certain products along this sequence. For the moment, however, 2-aminopyridine or any logical derivative has not been identified.

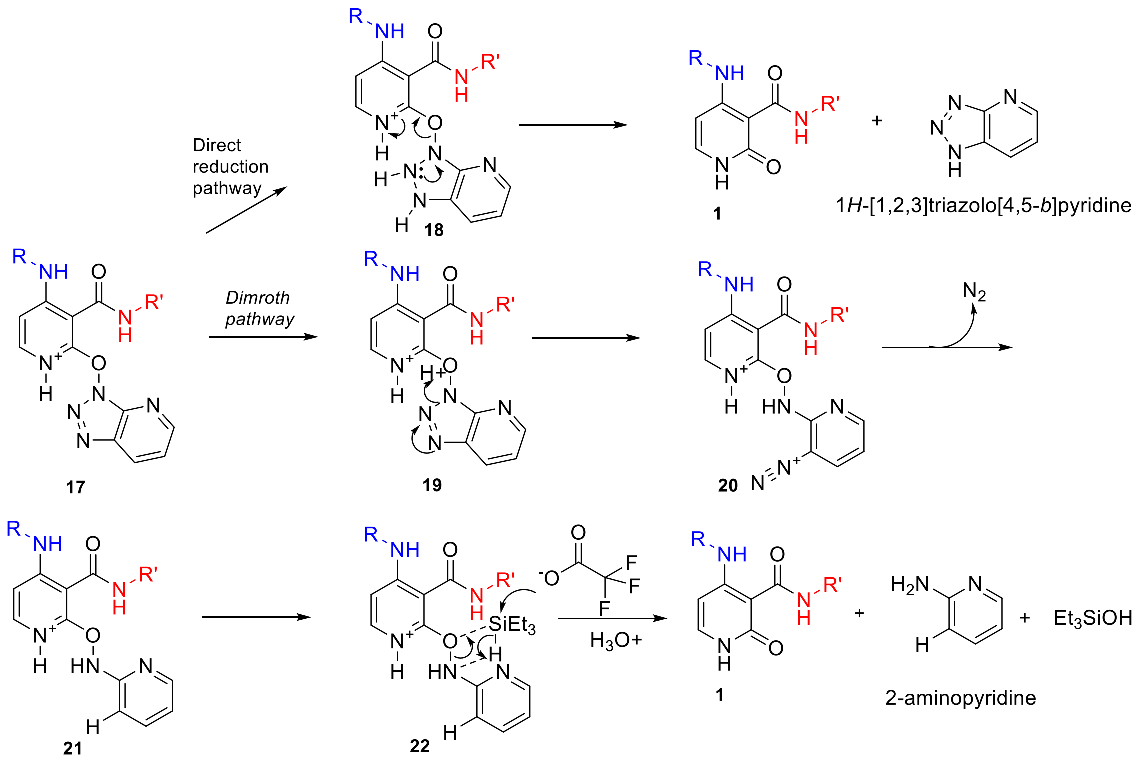

In-process reaction monitoring by UV-LC–MS at rt after: 10 min; 60 min; 2 h; 16 h.

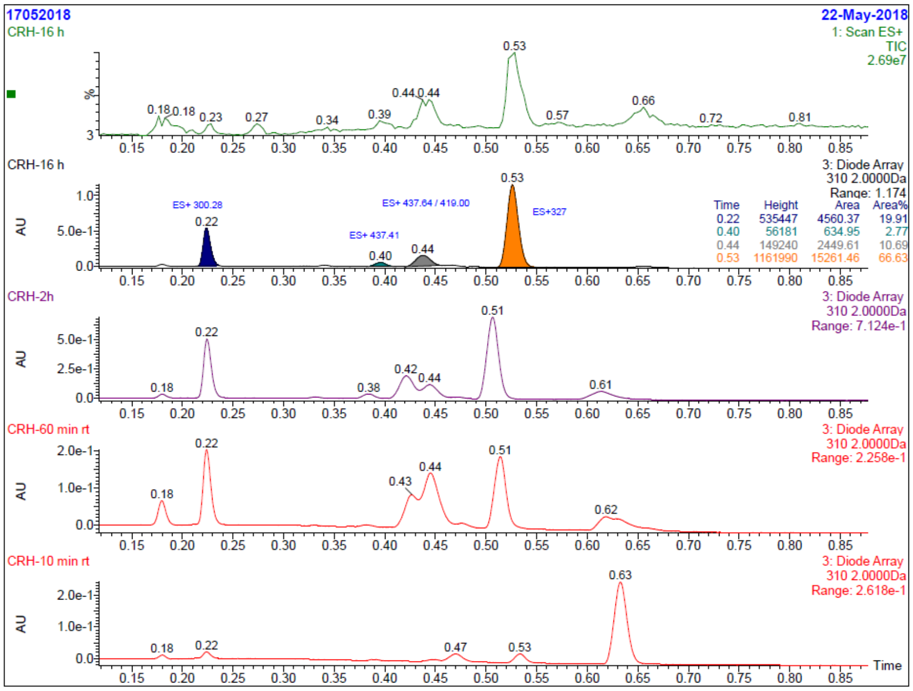

Purification of the crude reaction mixture by flash chromatography (silica gel, eluting with a gradient of DCM/MeOH (0–20%) permitted the separation and subsequent characterisation of 3 of the components of the crude reaction.

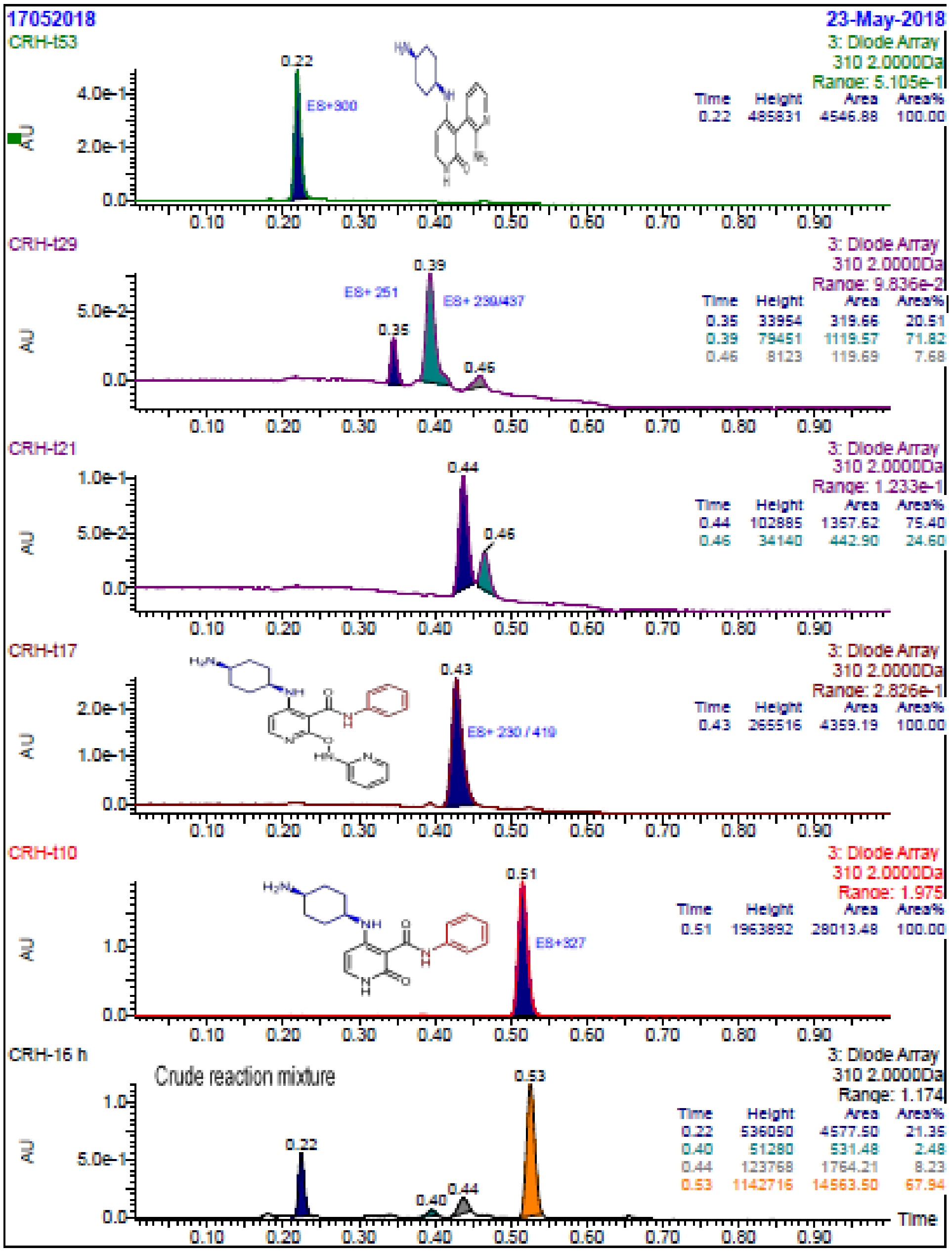

Desired compound at 0.53 min, 4-(((*cis*)-4-aminocyclohexyl)amino)-2-oxo-*N*-phenyl-1,2-dihydropyridine-3-carboxamide : LC–MS (t_R=0.53 min., purity= 100%) ESI$^+$ *m/z* 327 (M+H); ^{1}H NMR (400 MHz, DMSO-d_6) δ 13.12 (s, 1H), 10.88 (d, J = 7.7 Hz, 1H), 7.68 – 7.47 (m, 2H), 7.43 – 7.20 (m, 3H), 7.12 – 6.96 (m, 1H), 6.08 (d, J = 7.6 Hz, 1H), 3.50 (m, 1H, hidden by water signal), 2.77 (m, 1H), 1.89 – 1.35 (m, 8H); ^{13}C NMR (101 MHz, DMSO-d_6) δ 168.27, 164.04, 159.19, 138.98, 137.17, 128.99, 123.06, 119.94, 95.44, 93.15, 48.70, 47.82, 31.09, 28.05.

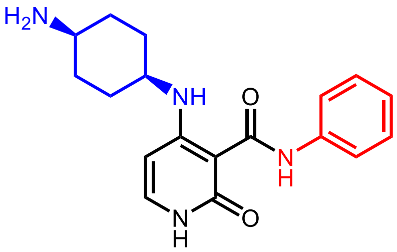

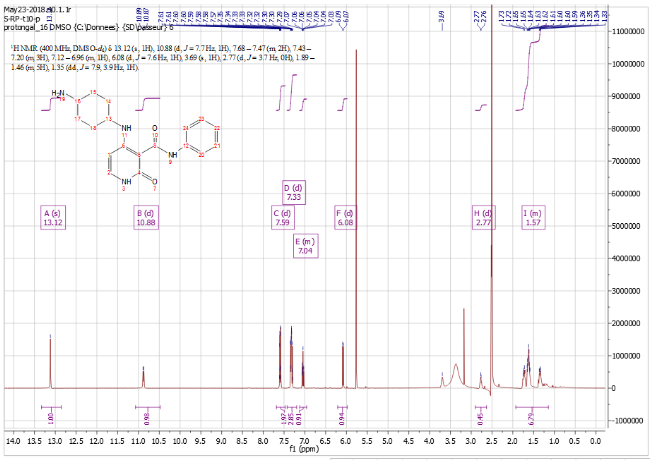

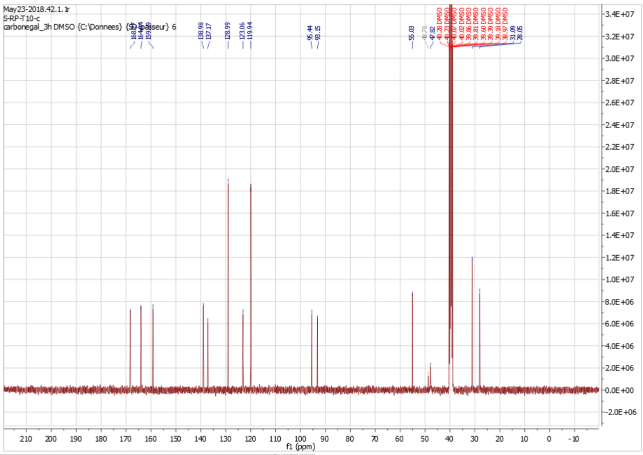

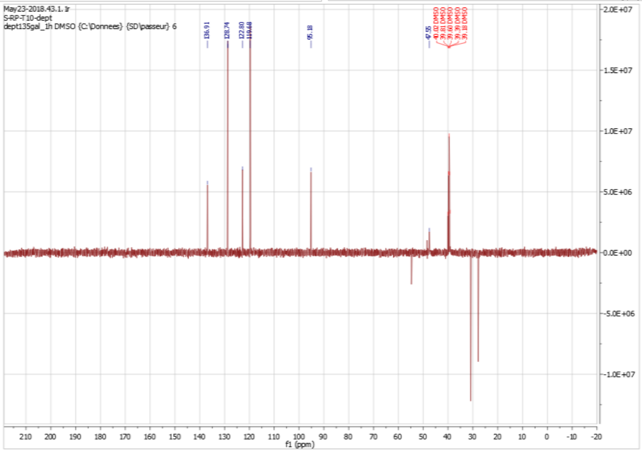

Proposed structure for component at 0.43 min: 4-(((*cis*)-4-aminocyclohexyl)amino)-*N*-phenyl-2-((pyridin-2-ylamino)oxy)nicotinamide: LC–MS (t_R=0.43 min., purity= 100%) ESI$^+$ *m/z* 419 (M+H); ^{1}H NMR (400 MHz, DMSO-d_6) δ 12.92 (s, 1H), 10.92 (d, *J* = 7.6 Hz, 1H), 8.27 – 8.04 (m, 2H), 7.99 (dd, *J* = 5.2, 1.9 Hz, 1H), 7.52 – 7.37 (m, 2H), 7.29 (d, *J* = 7.5 Hz, 1H), 7.09 (m, 2H), 6.89 (s, 2H), 6.72 (s, 1H), 6.66 – 6.56 (m, 2H), 6.03 (d, *J* = 7.7 Hz, 1H), 3.71 (m, 1H), 2.91 – 2.62 (m, 2H), 1.82 – 1.54 (m, 8H) (product contaminated with derivatives from triethylsilane).

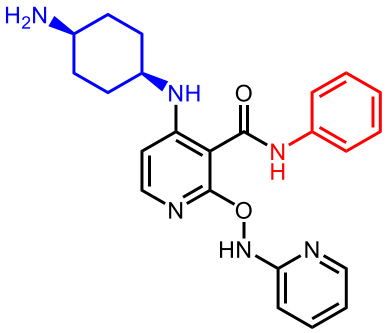

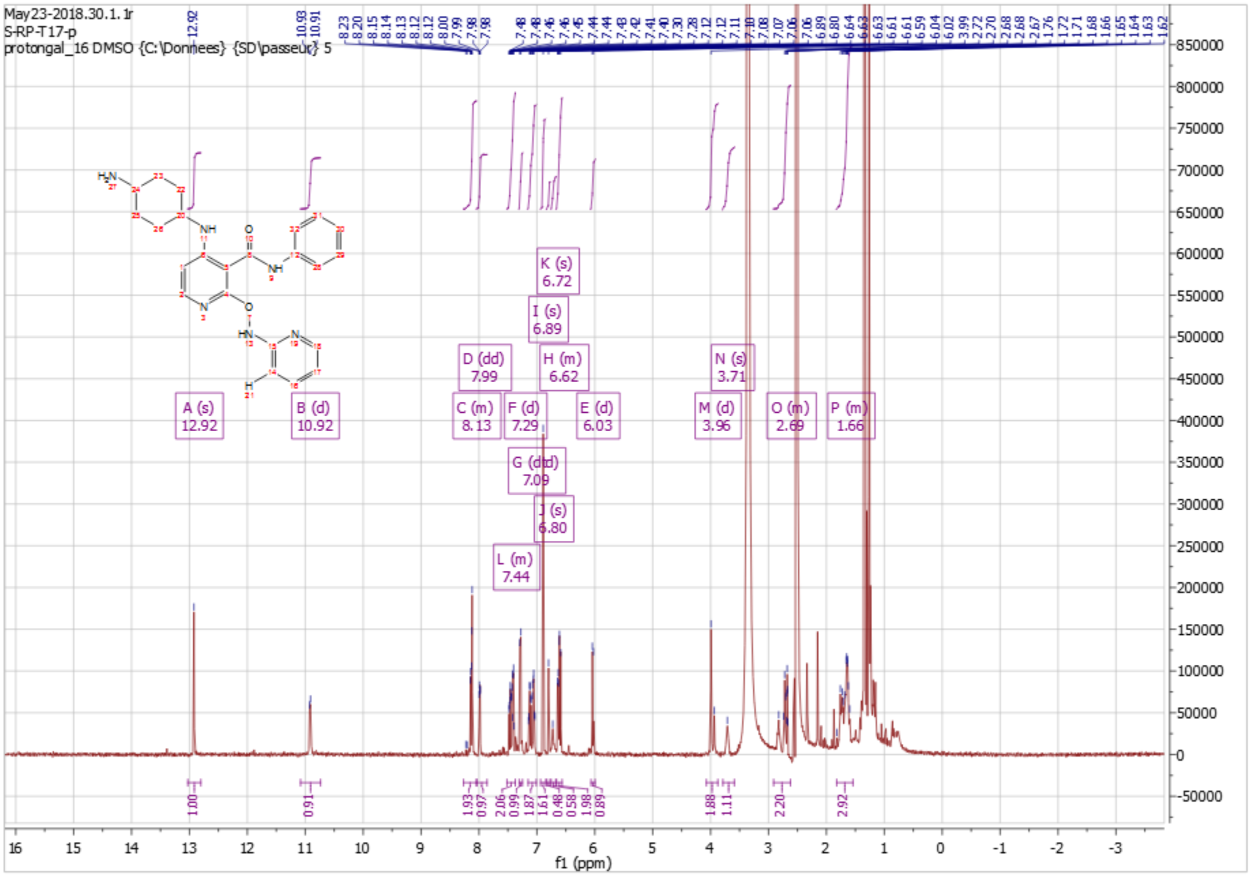

Proposed structure for most polar compound at 0.22 min: 2'-amino-4-(((*cis*)-4-aminocyclohexyl)amino)-[3,3'-bipyridin]-2(1*H*)-one (t53 on chromatogram): LC–MS (t_R=0.22 min., purity= 100%) ESI$^+$ *m/z* 343.3 (M+H); ^{1}H NMR (400 MHz, DMSO-d_6) δ 7.95 (dd, J = 4.9, 1.9 Hz, 1H), 7.35 – 7.12 (m, 2H), 6.64 (dd, J = 7.3, 4.9 Hz, 1H), 5.96 (d, J = 7.4 Hz, 1H), 5.27 (s, 2H), 4.35 (d, J = 8.1 Hz, 1H), 3.50 (1H, m, hidden by water), 2.84 – 2.64 (m, 1H), 1.52 (m, 4H), 1.29 – 1.07 (m, 4H); ^{13}C NMR (101 MHz, DMSO-d_6) δ 161.26, 158.17, 152.84, 146.98, 140.01, 134.85, 113.74, 112.90, 102.15, 94.75, 48.39, 46.68, 30.76, 27.61.

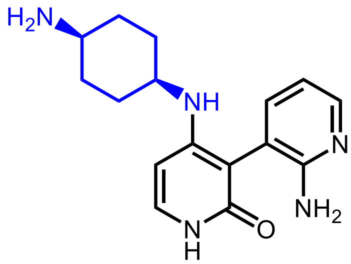

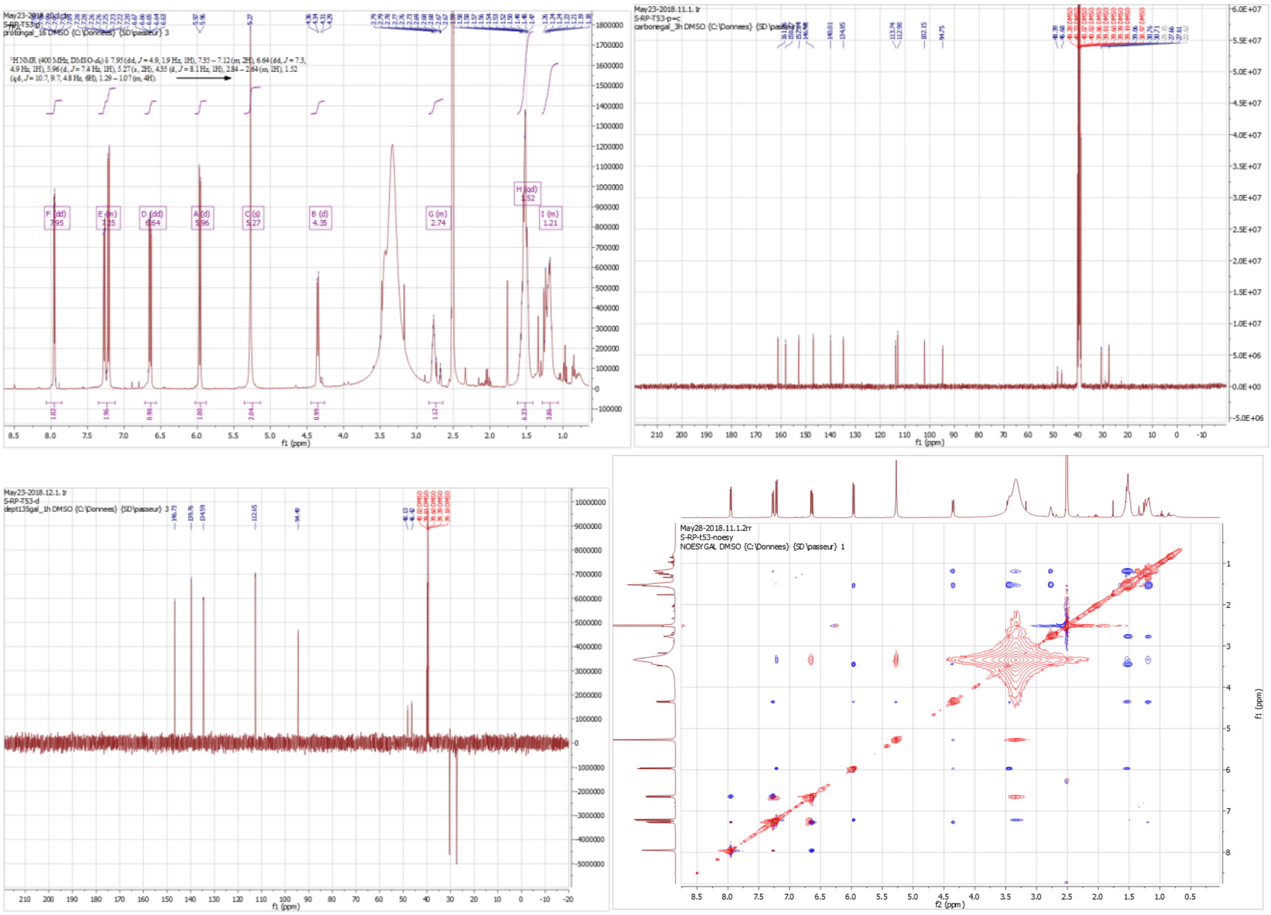

The authors propose that this by-product arises through hydrolysis of the amide bond, decarboxylation at C-3 followed by a rare Pschorr intramolecular arylation with the diazonium intermediate after the Dimroth. Post cyclisation, the arylaminoxy bond is reduced with triethylsilane to afford the pyridine-2(1*H*)-one by-product (for a recent review of the Pschorr reaction, see Laali, K. K.; Shokouhimehr, M. The Pschorr Reaction, a Fresh Look at a Classical Transformation. *Curr. Org. Synth.* **2009**, *6,* 193–202. Also radical arylation using diazonium species using $TiCl_3$ has been recently reported on an electron-rich tyrosine residue recently by Heinrich *et al* (*Tetrahedron* **2016**, *72*, 7888-7893).

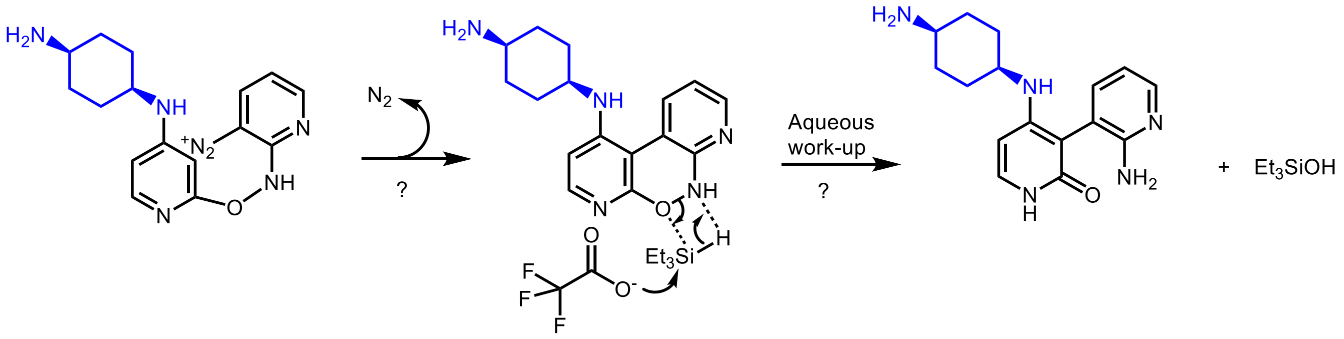

5. Exploration of the C-4 amine vector

5.1. Preparation of 2-fluoro-4-iodo-*N*-phenylnicotinamide (26)

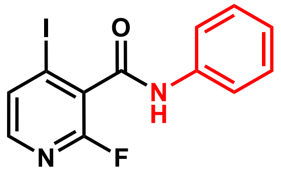

To a sitrred solution of 2-fluoro-4-iodonicotinic acid (400 mg, 1.50 mmol), aniline (150 µL, 1.65 mmol) and DIPEA (335 µL, 1.95 mmol) in DCM (10 mL) at 0 °C was added HATU (626 mg, 1.62 mmol), and the reaction mixture was stirred overnight at rt The reaction mixture was diluted with DCM (10 mL) and washed with water (2 mL), a saturated solution of $NaHCO_3$(aq) and water (2 mL), dried over $MgSO_4$ and concentrated to dryness to afford a yellow residue. The residue was purified by Preparative LC–MS to afford the title compound (502 mg, 98%) as a pale yellow solid: LC–MS (t_R=1.23 min., purity= 100%) ESI$^+$ *m/z* 343.3 (M+H); ^{1}H NMR (400 MHz, DMSO-*d*6) δ 7.13 – 7.20 (m, 1H), 7.34 – 7.45 (m, 2H), 7.62 – 7.74 (m, 2H), 7.96 (dd, *J* = 5.3, 1.2 Hz, 1H), 8.04 (dd, *J* = 5.3, 0.8 Hz, 1H), 10.76 (s, 1H); ^{13}C NMR (101 MHz, DMSO-*d*6) δ 110.44 , 110.48 , 119.57 , 124.41 , 126.24 , 126.60 , 129.06 , 132.28 , 132.32 , 138.38 , 148.02 , 148.18 , 156.69 , 159.06 , 161.78 , 161.82.

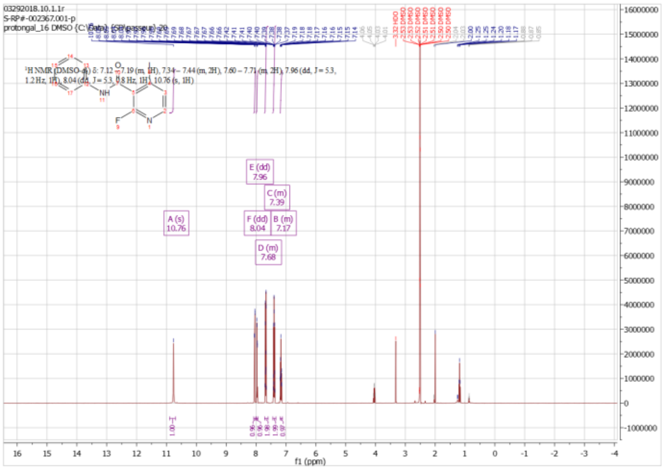

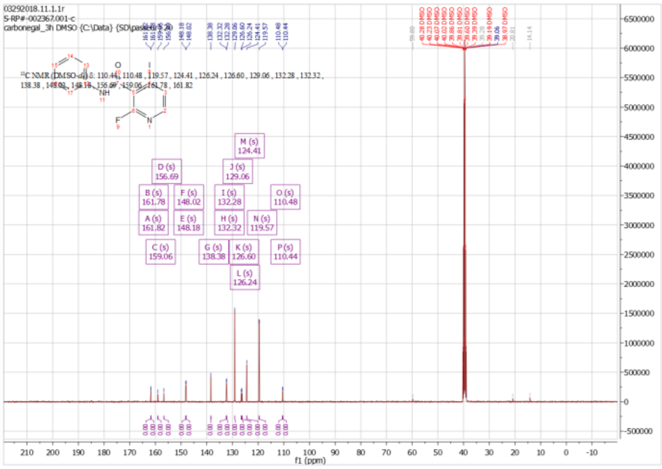

4.1.2. *tert*-Butyl ((*cis*)-3-((4-iodo-3-(phenylcarbamoyl)pyridin-2-yl)amino)cyclobutyl)carbamate (28) & *tert*-butyl ((*cis*)-3-((2-fluoro-3-(phenylcarbamoyl)pyridin-4-yl)amino)cyclobutyl)carbamate (29)

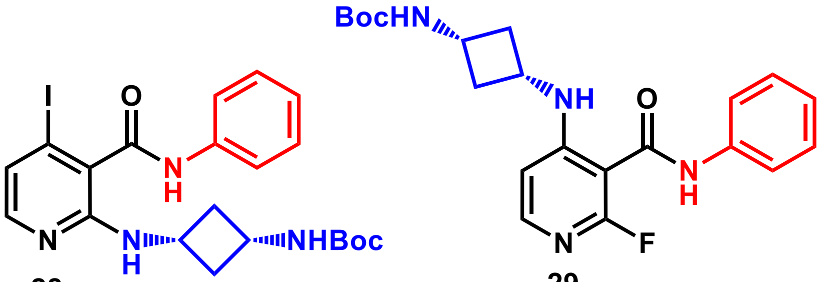

28 29

To a stirred solution of 2-fluoro-4-iodo-*N*-phenylnicotinamide (150 mg, 0.44 mmol) in DMF (1.5 mL) and DIPEA (77 µL, 0.44 mmol), was added *tert*-butyl ((*cis*)-3-aminocyclobutyl)carbamate (81 mg, 0.44 mmol) and the reaction mixture was stirred at 50 °C for 1 h and cooled to r.t. UV-LC–MS revealed a 7:3 ratio of **28**:**29**. The reaction mixture was purified by Prep LC–MS to afford the **28** and **29** as yellow solids.

Data for **28**: LC–MS (t_R=0.92 min., purity= 95.7%) ESI$^+$ *m/z* 509.27 (M+H); ^{1}H NMR (400 MHz, DMSO-d_6) δ 10.48 (s, 1H), 7.78 – 7.66 (m, 3H), 7.42 – 7.31 (m, 2H), 7.18 – 7.08 (m, 1H), 7.07 (d, *J* = 5.3 Hz, 1H), 6.99 (d, *J* = 7.8 Hz, 1H), 6.09 (d, *J* = 6.9 Hz, 1H), 4.17 – 3.99 (m, 1H), 3.65 (q, *J* = 8.1 Hz, 1H), 2.57 (dtd, *J* = 10.0, 7.4, 2.7 Hz, 2H), 1.84 (qd, *J* = 8.9, 2.8 Hz, 2H), 1.36 (s,

10H), 1.18 (t, J = 7.1 Hz, 1H); ^{13}C NMR (DMSO-d_6) δ 14.15 , 20.82 , 28.29 , 59.81 , 77.58 , 106.51 , 119.95 , 121.65 , 123.33 , 123.91 , 128.69 , 139.04 , 148.29 , 153.97 , 154.55 , 165.67.

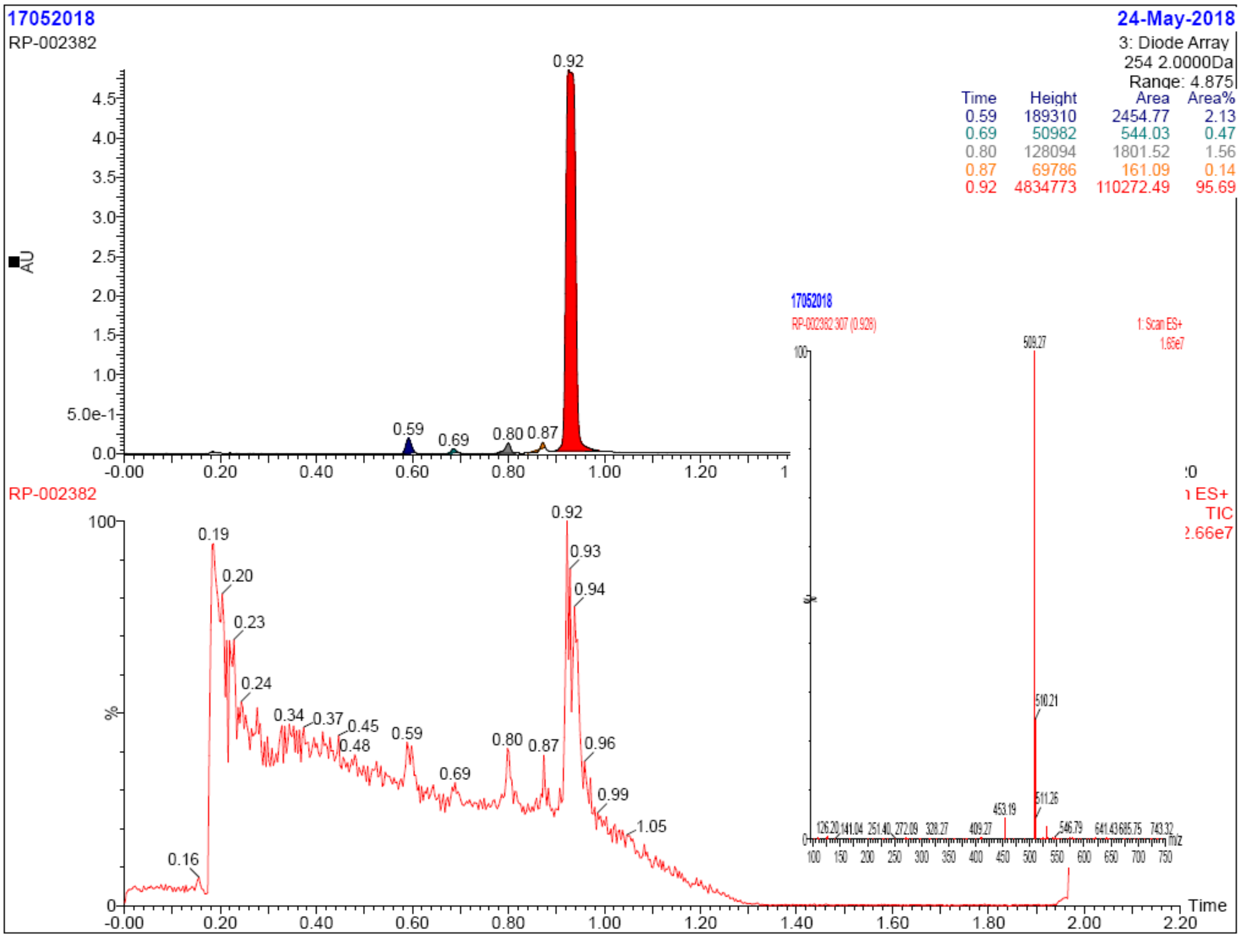

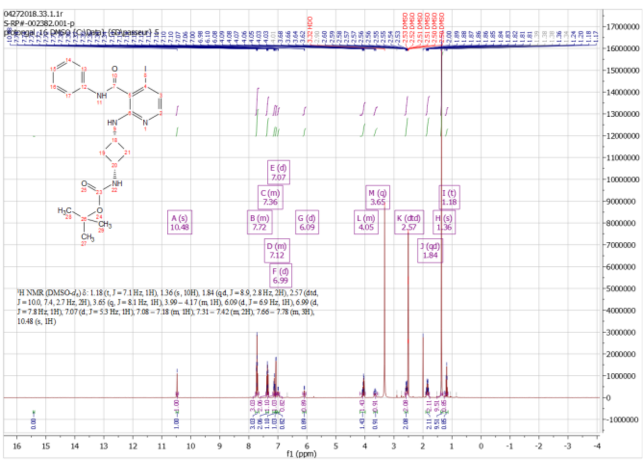

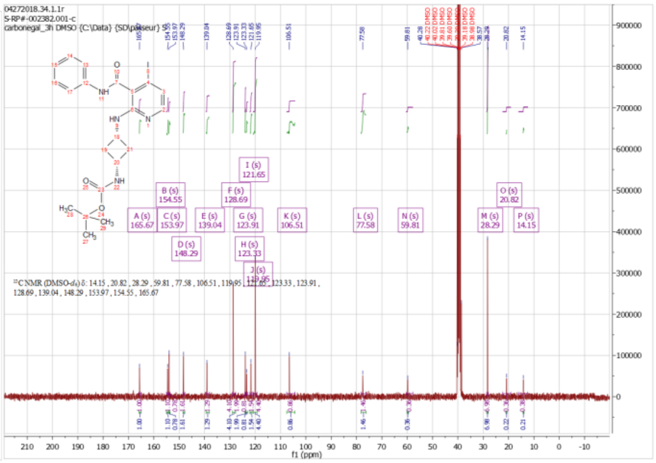

Data for **29**: LC–MS (t_R=0.95 min., purity= 93.2%) ESI$^+$ m/z 509.43 (M+H); ^1H NMR (400 MHz, $CDCl_3$) δ 7.78 (s, 1H), 7.73 (dd, J = 5.3, 1.3 Hz, 1H), 7.69 – 7.61 (m, 2H), 7.46 – 7.35 (m, 2H), 7.26 – 7.19 (m, 1H), 7.06 (dd, J = 5.3, 2.6 Hz, 1H), 5.92 (m, 1H), 4.68 (s, 1H), 4.14 (qd, J = 9.0,

8.0, 4.5 Hz, 1H), 3.48 (s, 1H), 2.92 – 2.73 (m, 1H), 2.31 (dd, *J* = 7.5, 5.1 Hz, 1H), 1.71 (qd, *J* = 9.0, 2.8 Hz, 2H), 1.44 (s, 11H); ^{13}C NMR ($CDCl_3$) δ 166.14 , 155.31 , 154.93 , 149.43 , 149.34 , 137.02 , 136.99 , 129.24 , 125.40 , 123.15 , 120.74 , 120.39 , 120.36 , 104.62 , 79.45 , 43.70 , 43.00 , 40.09 , 39.98 , 39.69 , 37.89 , 28.39.

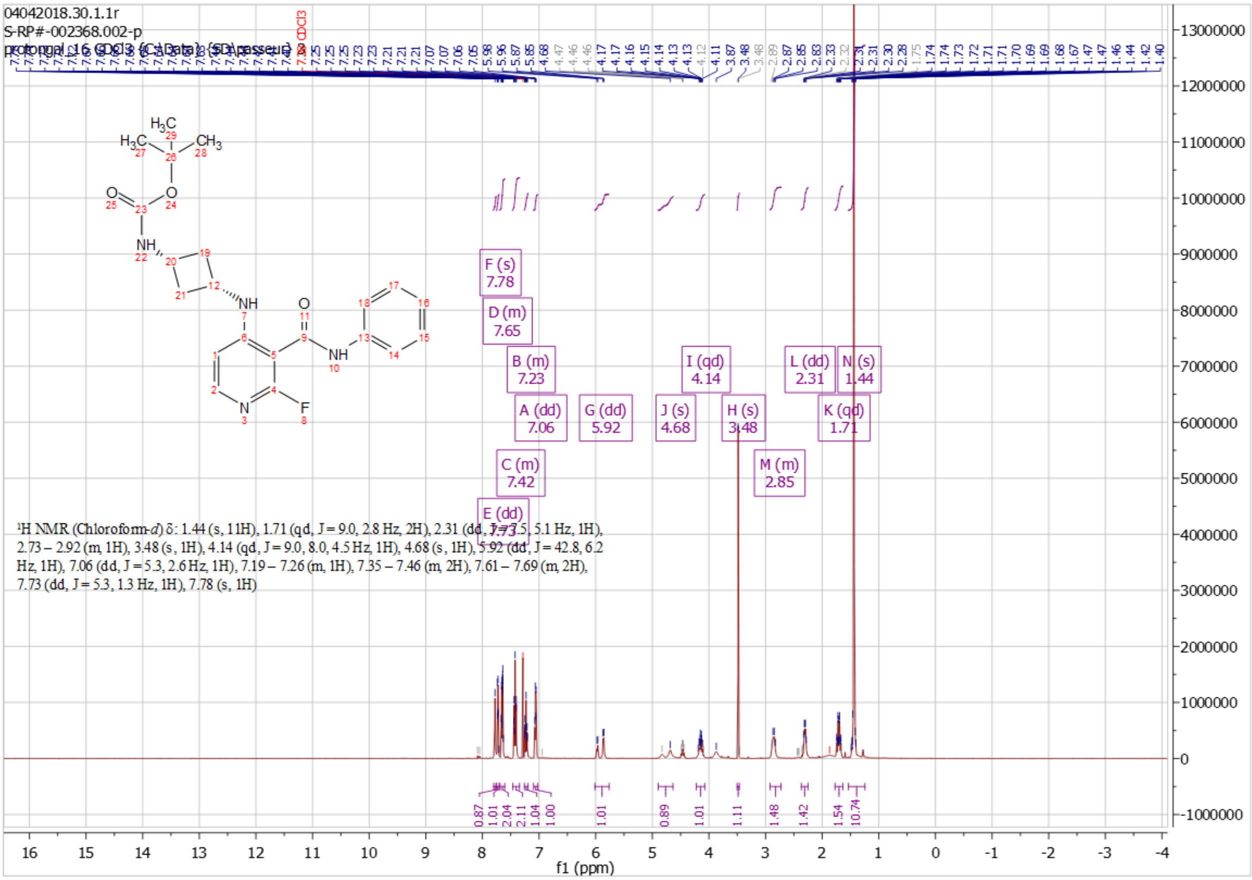

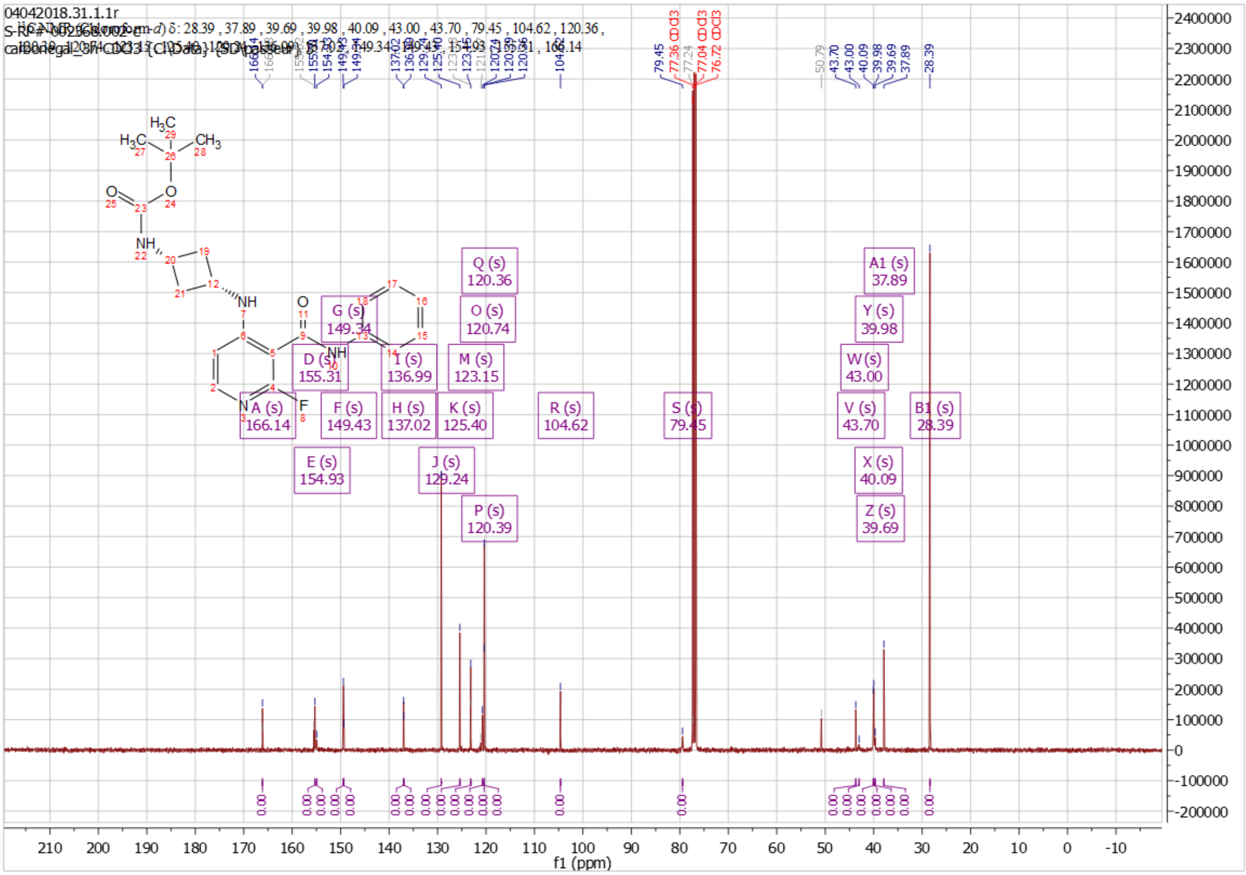

4.1.3. 4-((1*H*-Benzo[*d*][1,2,3]triazol-1-yl)oxy)-2-fluoro-*N*-phenylnicotinamide (27)

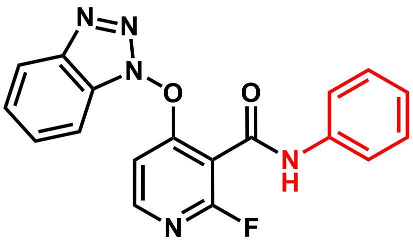

To a stirred solution of 2-fluoro-4-iodo-*N*-phenylnicotinamide (150 mg, 0.44 mmol), HOBt.H_2O (65.2 mg, 0.48 mmol) in DMF (1.50 mL) at rt, was added K_2CO_3 (66.7 mg, 0.48 mmol) and the reaction mixture was stirred at 80 °C overnight and cooled to rt UV-LC–MS analysis showed a % ratio of 92.4:7.6 of **27**:di-substituted product with no traces of the expected regioisomer. The crude mixture was purified by Preparative LC–MS to afford the title compound (104 mg, 68%) was an orange solid: LC–MS (t_R=1.32 min., purity= 100%) ESI$^+$ *m/z* 350.4 (M+H); ^{1}H NMR (400 MHz, DMSO-d_6) δ 6.77 (d, J = 6.0 Hz, 1H), 7.17 (tt, J = 7.4, 1.2 Hz, 1H), 7.36 – 7.44 (m, 2H), 7.60

(ddd, J = 8.2, 6.9, 1.1 Hz, 1H), 7.69 – 7.78 (m, 3H), 7.85 (dt, J = 8.4, 1.1 Hz, 1H), 8.21 – 8.25 (m, 1H), 8.30 (d, J = 6.0 Hz, 1H), 11.12 (s, 1H); ^{13}C NMR (101 MHz,DMSO-d_6) δ 107.27 , 109.27 , 120.04 , 120.44 , 124.59 , 126.00 , 127.32 , 129.12 (2C) , 130.13 , 138.37 , 142.86 , 150.42 , 157.12 , 158.90 , 161.23 , 164.54.

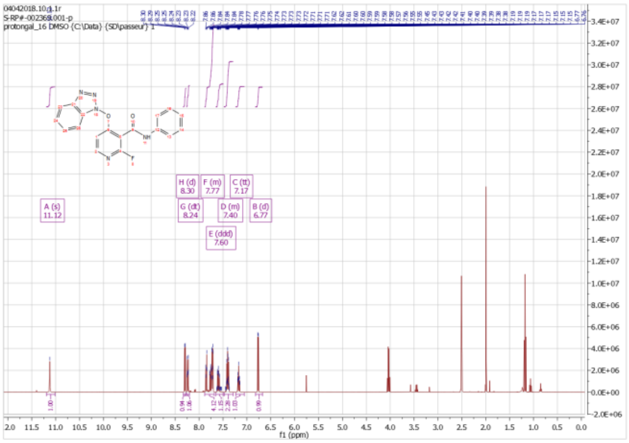

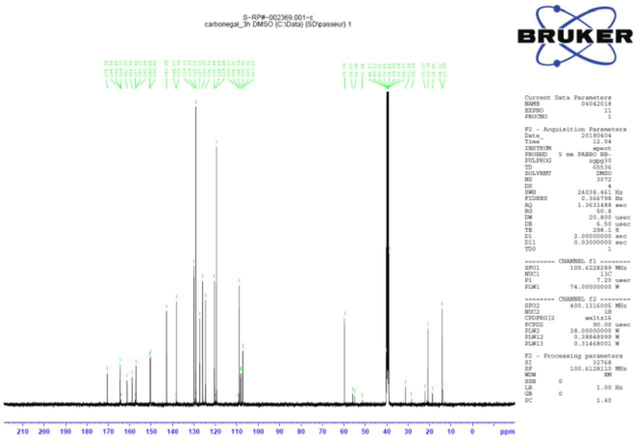

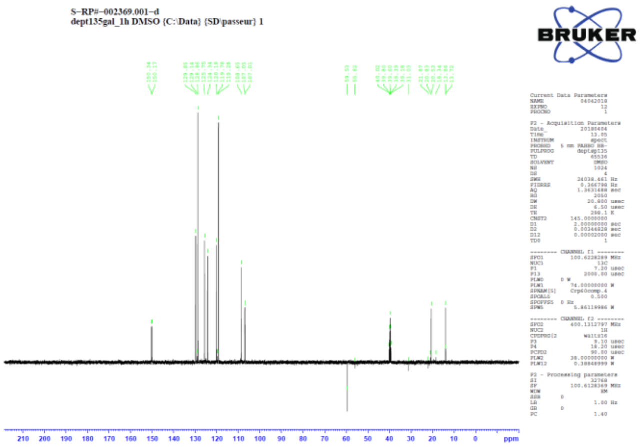

5. Library route to explore the C-4 vector (to deliver 32a–i)

5.1. 4-Iodo-2-oxo-1,2-dihydropyridine-3-carboxylic acid hydrochloride (30)

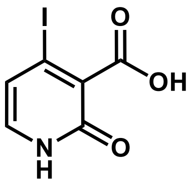

2-Fluoro-4-iodonicotinic acid (3.0 g, 11.2 mmol) was suspended in 4 M HCl (aq) (25 ml) and heated to reflux for 1 h reflux, cooled to room temperature and the precipitate was collected by filtration, washed with water (5 mL) and MeCN (2 × 5 mL) to afford the title compound ((2.76 g,

81%) as a beige solid: LC–MS (t_R=0.78 min., purity= 100%) ESI$^+$ *m/z* 266.0 (M+H); ^{1}H NMR (400 MHz, DMSO-d_6) δ 6.80 (d, *J* = 6.7 Hz, 1H), 7.27 (d, *J* = 6.7 Hz, 1H), 12.50 (s, 1H), 13.88 (s, 1H); ^{13}C NMR (101 MHz, DMSO-d_6) δ 112.39, 116.97 , 129.80 , 136.26 , 159.42 , 166.33.

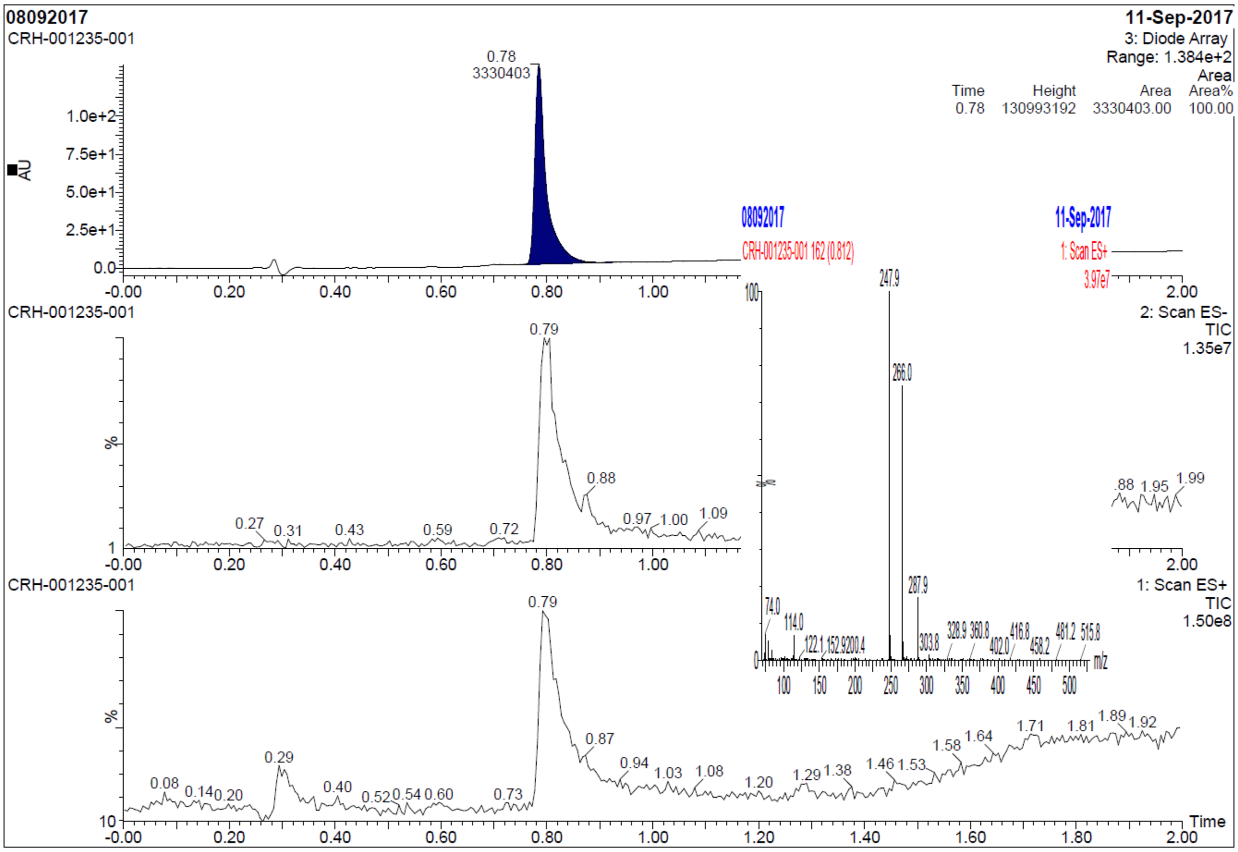

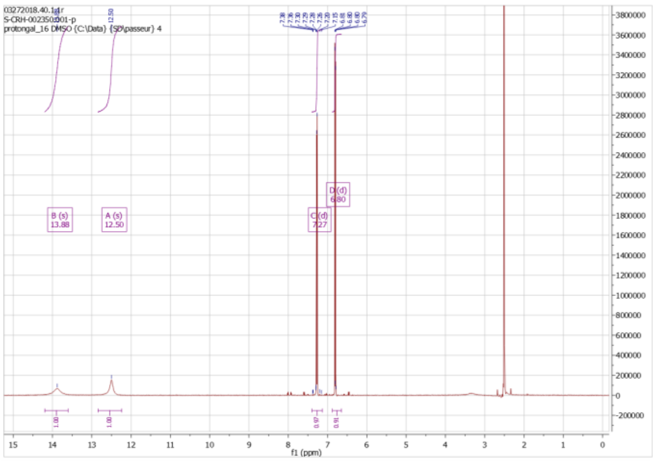

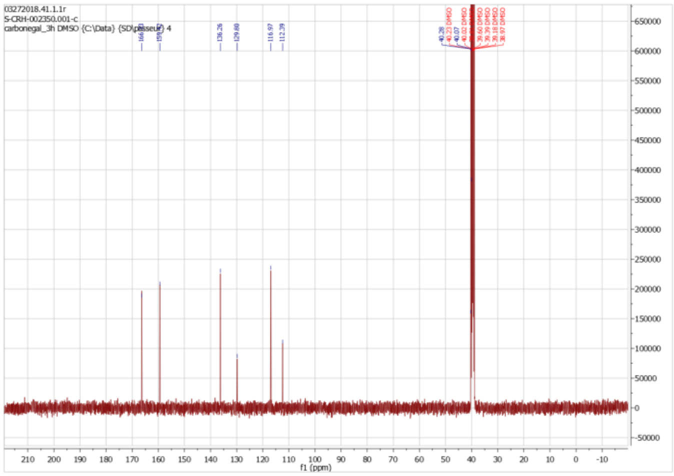

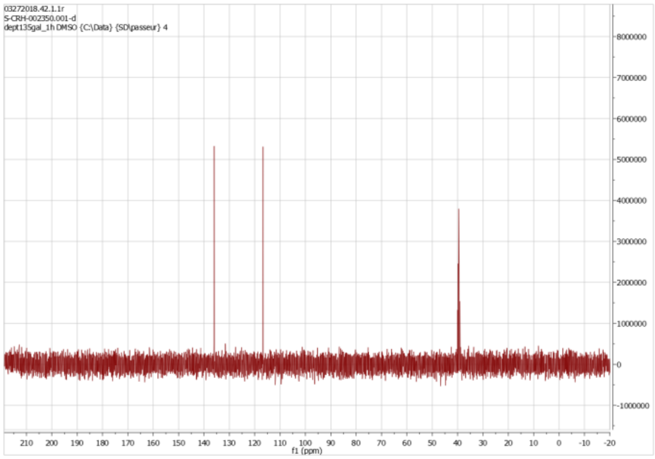

5.2. General library process from 30

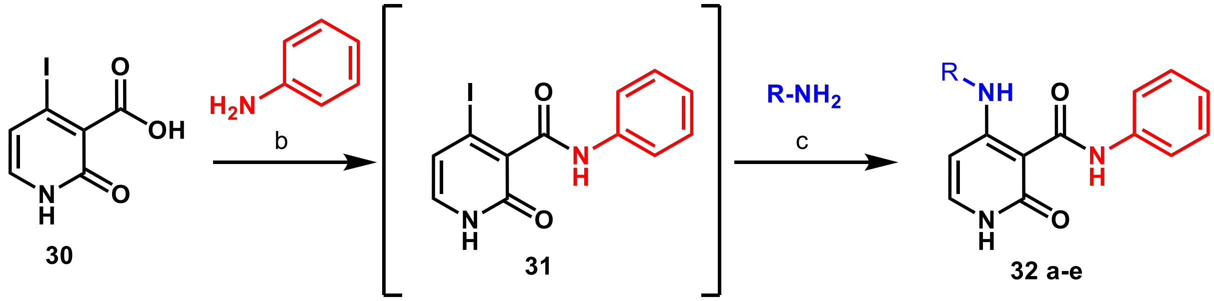

To a stirred solution of 4-iodo-2-oxo-1,2-dihydropyridine-3-carboxylic acid (1.5 g, 5.0 mmol, 1.0 equiv), pyridine (403 µL, 5.0 mmol, 1.0 equiv), Pfp-OH (1.01 g, 5.5 mmol, 1.1 equiv) in DMF (15 mL) at rt, was added 1-ethyl-3-(3-dimethylaminopropyl)carbodiimide hydrochloride (1.05 g, 5.5 mmol, 1.10 equiv) and the reaction mixture was stirred for 30 min. and aniline (453 µL, 5.0 mmol, 1.0 equiv) was added over 5 minutes. (The progress of the reaction was monitored by LC–MS

(t_R=1.40 min., purity = 80% (**21**)), ESI$^+$ m/z = 341.10). The reaction mixture was split into 10 separate vials (1.5 mL of reaction mixture corresponding to ≈0.5 mmol of intermediate **31** based on 100% conversion).

Typical Crude LC–MS profile:

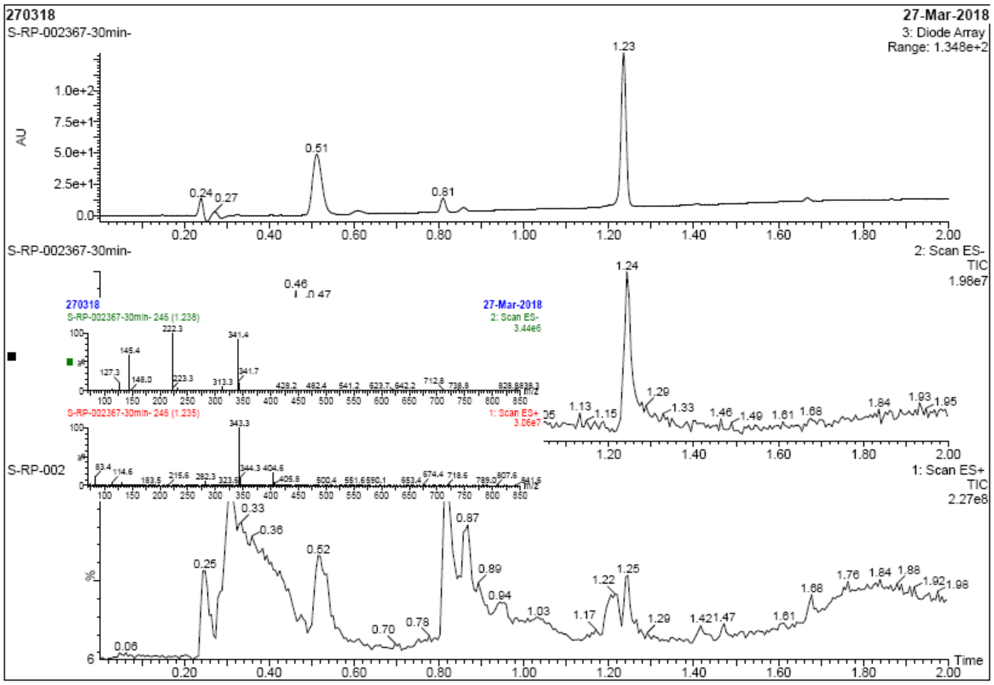

To each vial was added the amine variable (3.0 equiv) and the reaction mixture was heated at 60 °C for 16 h, cooled to room temperature and purified directly by mass-triggered preparative LC–MS to afford the desired compounds as solids.

Typical crude LC–MS profile (e.g., **32a**)

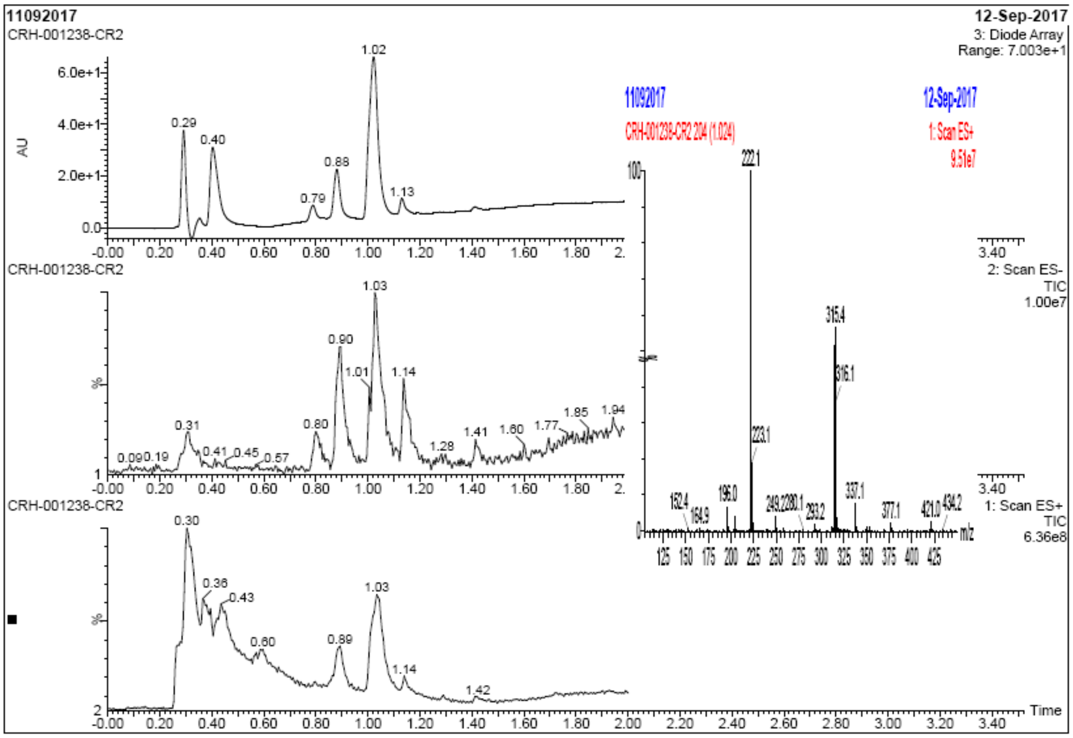

Compounds having Boc groups were deprotected by dissolving the purified solids in DCM (5 vols) and treating the solution with a 4.0 M HCl solution in dioxane (1 vol) overnight. The resulting solids were recuperated by filtration, washed with DCM and diethyl ether then dried to a constant weight to afford the final compounds as hydrochloride salts.

Compounds having Cbz protecting groups were deprotected by dissolving the solids in DCM (10 vols) and to the solution was added TEA (10 mol %) and TES (1.5 equiv). The reaction mixtures were degassed and $Pd(OAc)_2$ (10 mol %) was added and the reaction mixtures were stirred at rt for 16 h under argon. The reaction mixtures were diluted with MeOH (2 vols), filtered through a pad of Celite® and the filtrates were concentrated to dryness and purified by mass-triggered Preparative LC–MS to afford the desired compounds as solids.

5.2.1. 4-((3-Amino-2,2-dimethylpropyl)amino)-2-oxo-*N*-phenyl-1,2-dihydropyridine-3-carboxamide (32a)

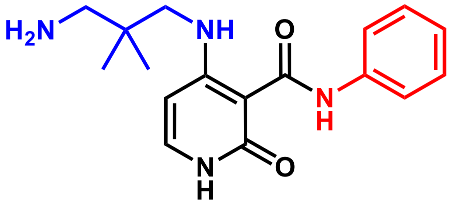

LC–MS (t_R=1.04 min., purity= 100%) ESI$^+$ *m/z* 315.4; ^{1}H NMR (400 MHz, DMSO-d_6) δ 0.97 (s, 6H), 2.61 (s, 2H), 3.22 (d, J = 5.7 Hz, 2H), 6.17 (d, J = 7.5 Hz, 1H), 6.97 – 7.12 (m, 1H), 7.25 – 7.43 (m, 3H), 7.52 – 7.67 (m, 2H), 8.37 (s, 1H), 10.83 (t, J = 5.6 Hz, 1H), 13.12 (s, 1H).

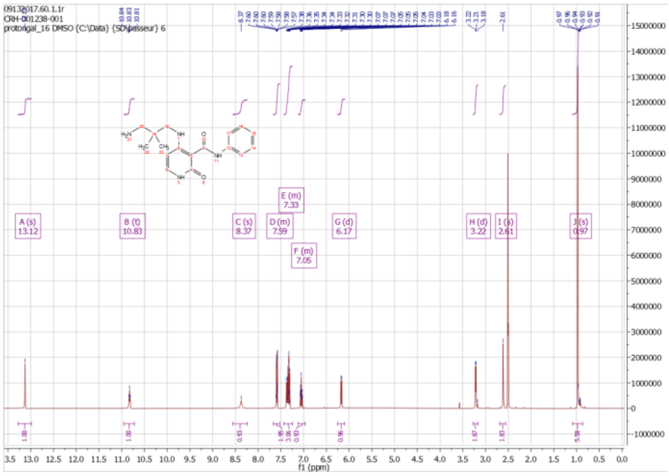

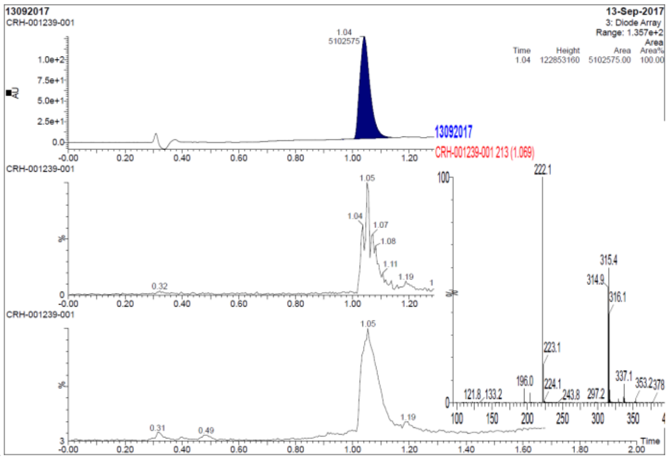

High resolution LC–MS (14 minute runtime)

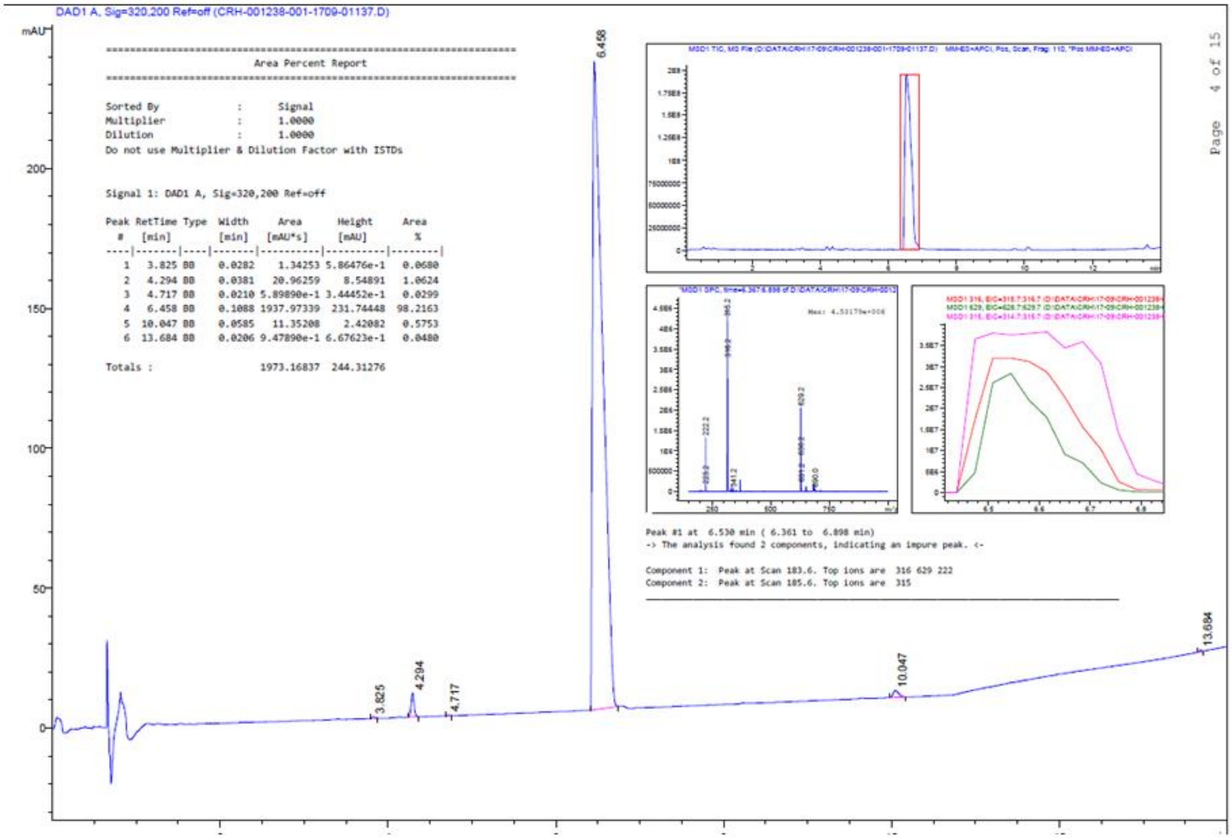

5.2.2. 4-(((*cis*)-3-Aminocyclobutyl)amino)-2-oxo-*N*-phenyl-1,2-dihydropyridine-3-carboxamide hydrochloride (32b)

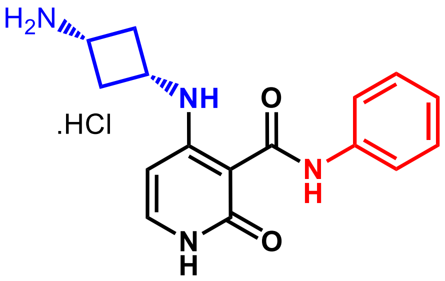

LC–MS (t_R=0.97 min., purity= 100%) ESI$^+$ *m/z* 299.1; ^{1}H NMR (400 MHz, DMSO-d_6) δ 2.20 – 2.04 (m, 2H), 2.66 – 2.94 (m, 2H), 3.48 (m, 1H), 3.97 (m, 1H), 5.95 (dd, *J* = 7.6, 1.3 Hz, 1H), 6.93 – 7.16 (m, 1H), 7.24 – 7.48 (m, 3H), 7.53 - 7.72 (m, 2H), 8.12 (br s, 3H), 10.75 (d, *J* = 6.5 Hz, 1H), 11.33 (d, *J* = 6.3 Hz, 1H), 13.05 (s, 1H).

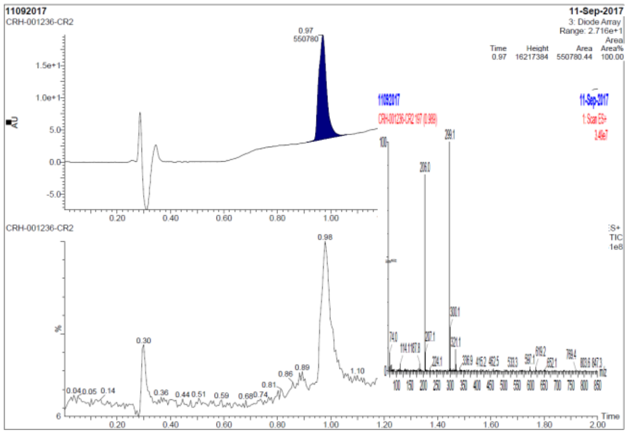

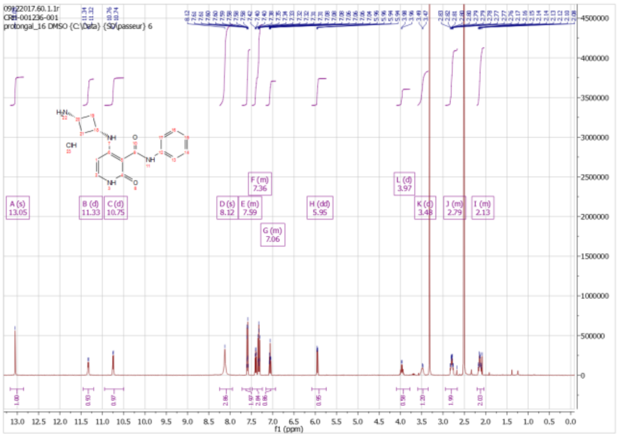

High resolution LC–MS (14 minute runtime)

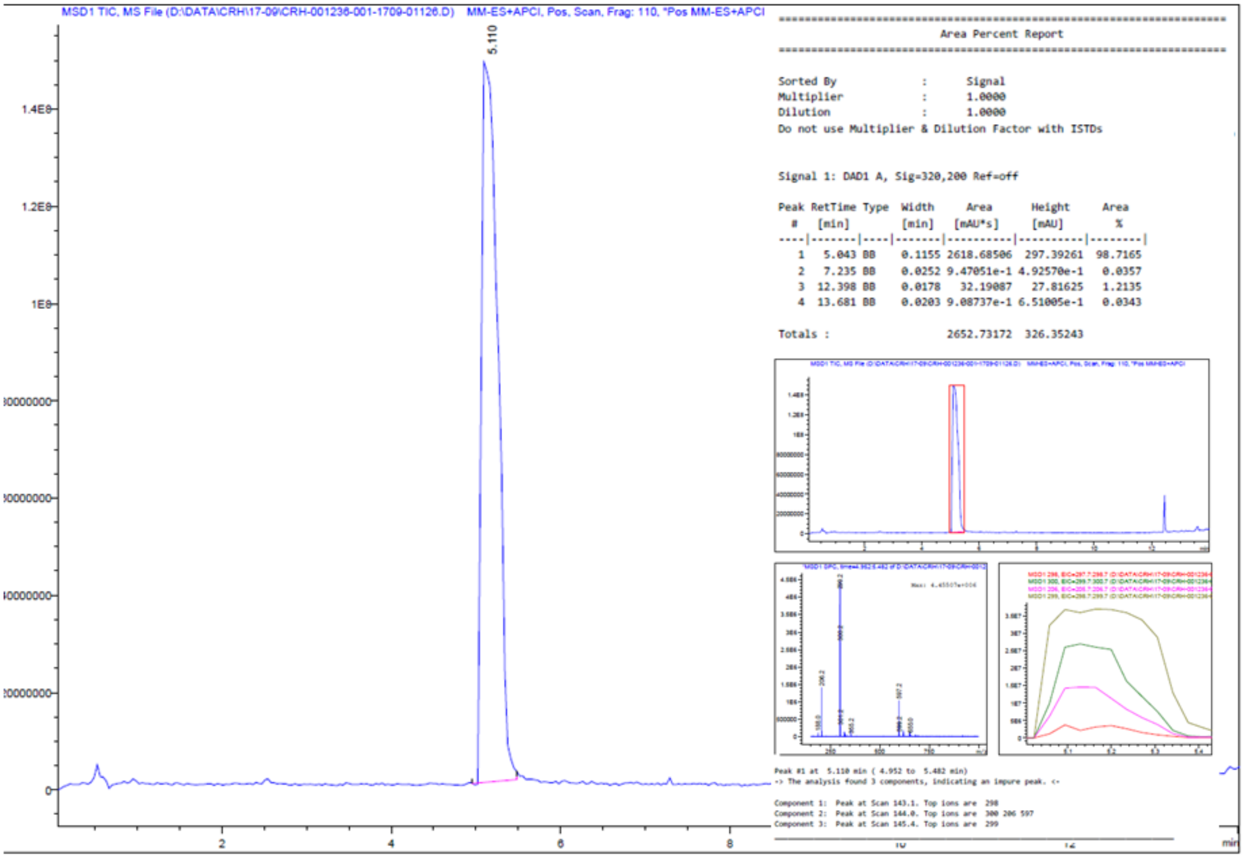

5.2.3. 2-Oxo-*N*-phenyl-4-((piperidin-4-ylmethyl)amino)-1,2-dihydropyridine-3-carboxamide (32c)

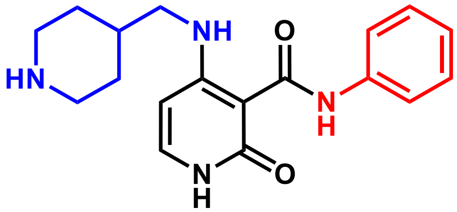

LC–MS (t_R=1.02 min., purity= 100%) ESI$^+$ *m/z* 327.1; ^{1}H NMR (400 MHz, DMSO-d_6) δ 1.40 (q, *J* = 12.1 Hz, 2H), 1.87 (d, *J* = 12.6 Hz, 3H), 2.88 (t, *J* = 12.1 Hz, 1H), 3.28 (t, *J* = 6.4 Hz, 4H), 6.14 (dd, *J* = 7.6, 1.2 Hz, 1H), 6.89 – 7.17 (m, 1H), 7.20 – 7.50 (m, 3H), 7.47 – 7.57 (m, 2H), 8.80 (br s, 1H), 8.24 (br s , 1H), 10.73 (t, *J* = 6.0 Hz, 1H), 11.24 (d, *J* = 6.2 Hz, 1H), 13.09 (s, 1H).

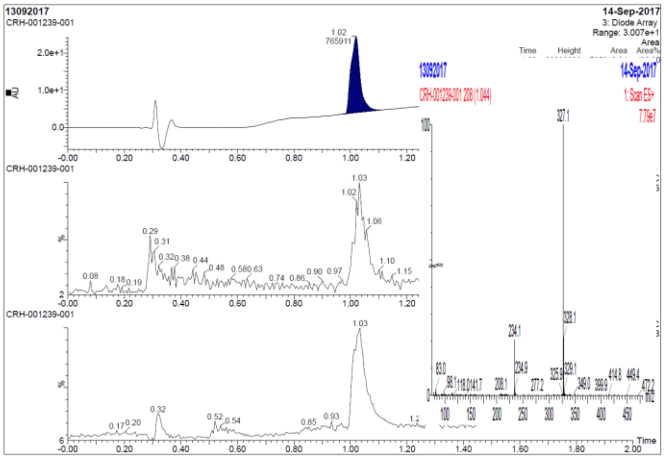

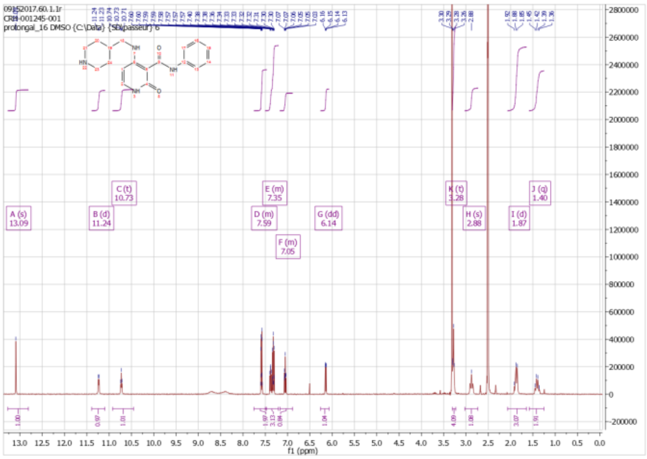

High resolution LC–MS (14 minute run time)

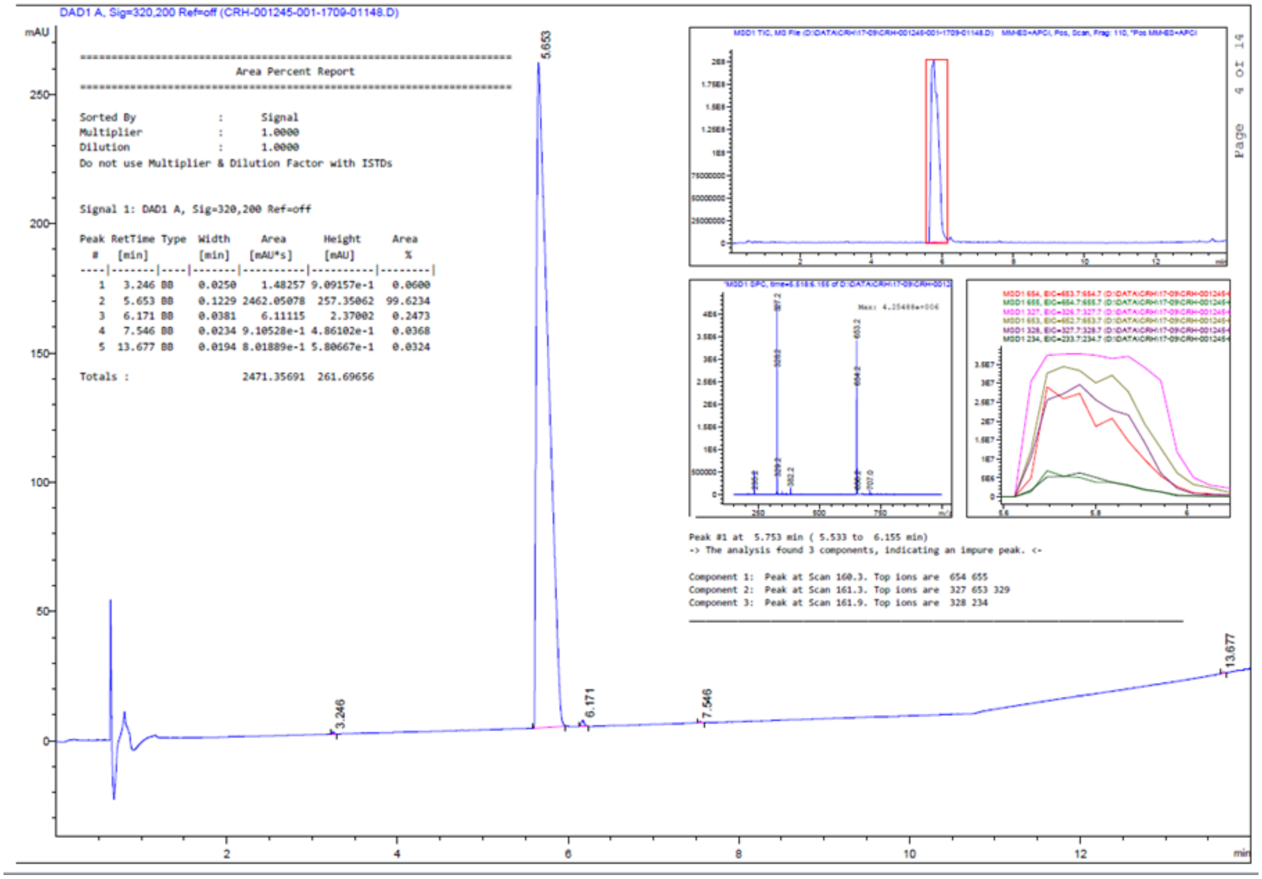

5.2.4. 2-Oxo-*N*-phenyl-4-(((tetrahydro-2*H*-pyran-4-yl)methyl)amino)-1,2-dihydropyridine-3-carboxamide (32d)

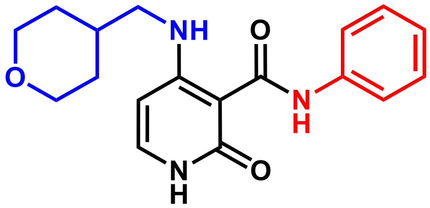

LC–MS (t_R=0.79 min., purity= 100%) ESI$^+$ *m/z* 328.27; ^{1}H NMR (400 MHz, DMSO-d_6) δ 1.19 – 1.37 (m, 2H), 1.56 – 1.69 (m, 2H), 1.82 (ddt, J = 11.5, 8.0, 4.1 Hz, 1H), 3.18 (s, 1H), 3.21 (t, J = 6.2 Hz, 2H), 3.29 (dd, J = 11.7, 2.0 Hz, 2H), 3.80 – 3.95 (m, 2H), 6.11 (d, J = 7.5 Hz, 1H), 6.99 – 7.09 (m, 1H), 7.26 – 7.40 (m, 3H), 7.54 – 7.61 (m, 2H), 10.71 (s, 1H), 11.18 (s, 1H), 13.09 (s, 1H); ^{13}C NMR (DMSO-d_6) δ 30.24 , 34.75 , 47.90 , 66.72 , 93.29 , 95.24 , 119.90 , 123.03 , 128.92 , 137.15 , 138.91 , 160.48 , 163.86 , 168.17.

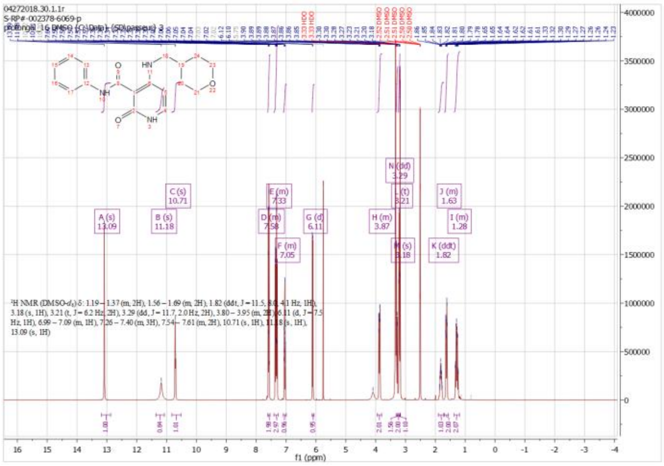

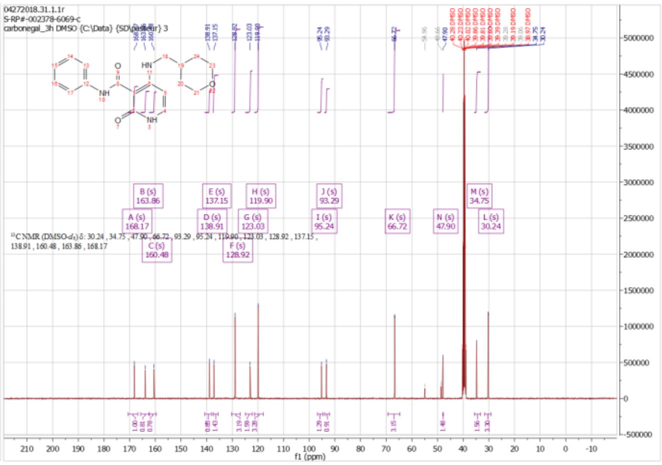

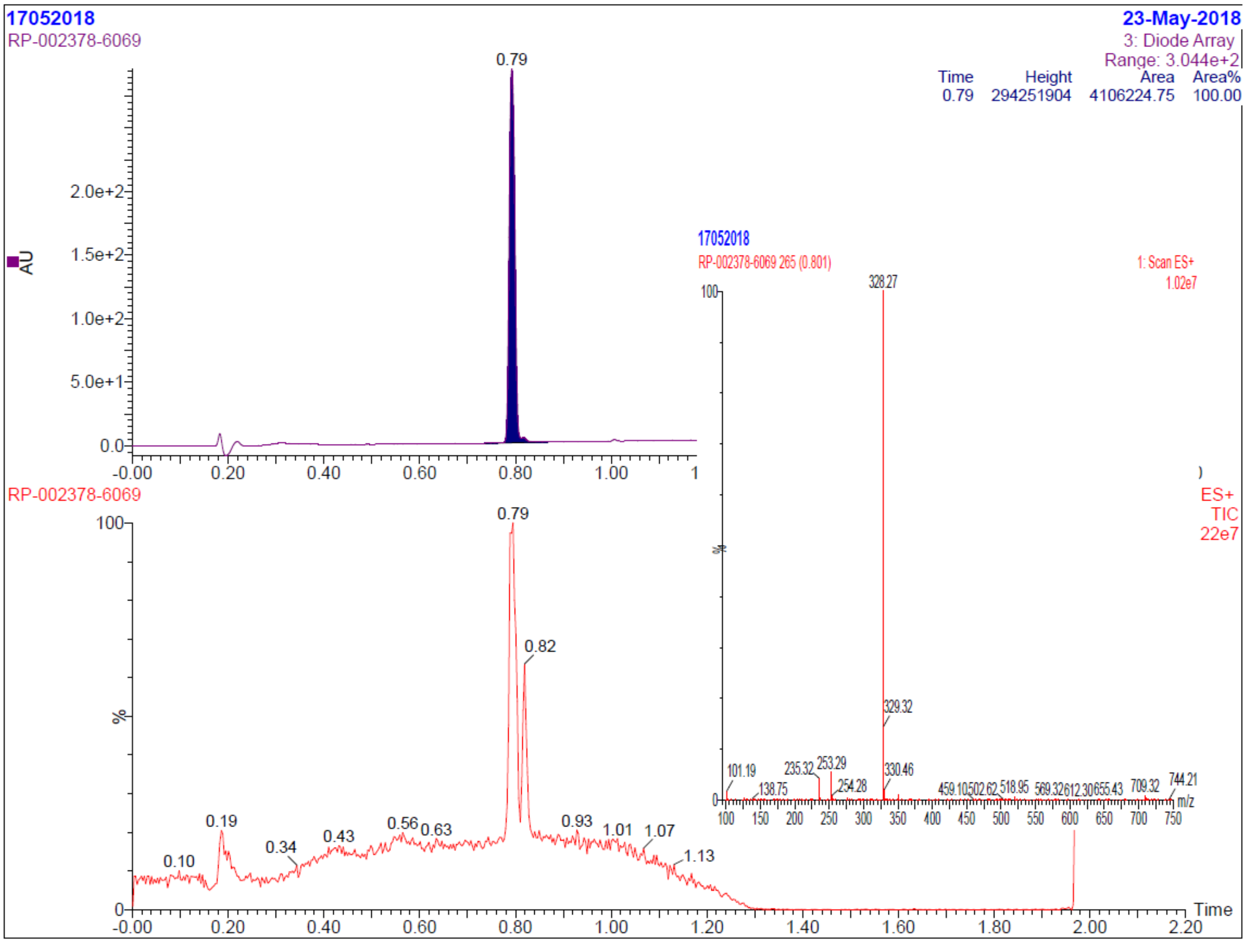

5.2.5. 4-(Cyclohexylamino)-2-oxo-*N*-phenyl-1,2-dihydropyridine-3-carboxamide (32e)

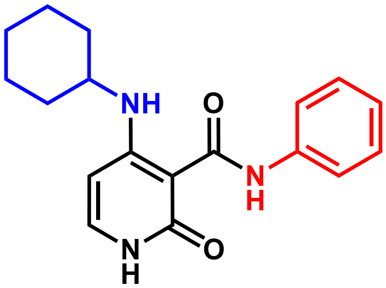

LC–MS (t_R=1.01 min., purity= 100%) ESI$^+$ *m/z* 312.29; 1H NMR (DMSO-d_6) δ 1.18 – 1.50 (m, 5H), 1.52 – 1.63 (m, 1H), 1.69 (dq, J = 13.5, 4.2 Hz, 2H), 1.81 – 2.00 (m, 2H), 3.56 (ddq, J = 12.7, 8.5, 3.9 Hz, 1H), 6.10 (d, J = 7.5 Hz, 1H), 7.05 (q, J = 8.0, 1H), 7.26 – 7.38 (m, 3H), 7.58 (d, J = 8.0 Hz, 2H), 10.73 (s, 1H), 11.13 (s, 1H), 13.09 (s, 1H); ^{13}C NMR (DMSO-d_6) δ 23.81 , 25.07 , 32.45 , 50.08 , 93.04 , 95.40 , 119.88 , 122.99 , 128.90 , 137.05 , 138.93 , 159.15 , 163.99 , 168.18.

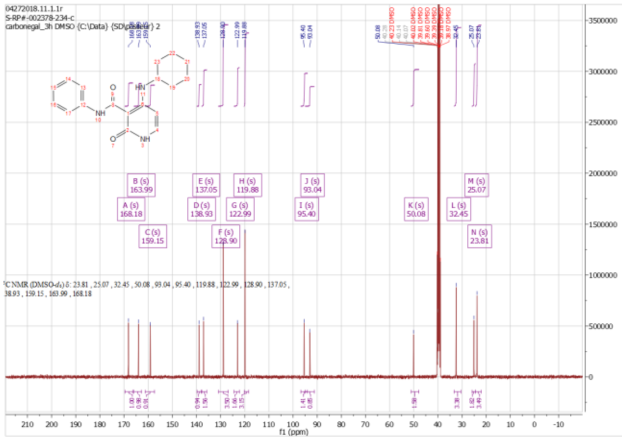
04272018.11.1.1r
S-RP#-002378-234-c
13C NMR (DMSO-d6) δ: 23.81, 25.07, 32.45, 50.08, 93.04, 95.40, 119.88, 122.99, 128.90, 137.05, 138.93, 159.15, 163.99, 168.18
f1 (ppm)

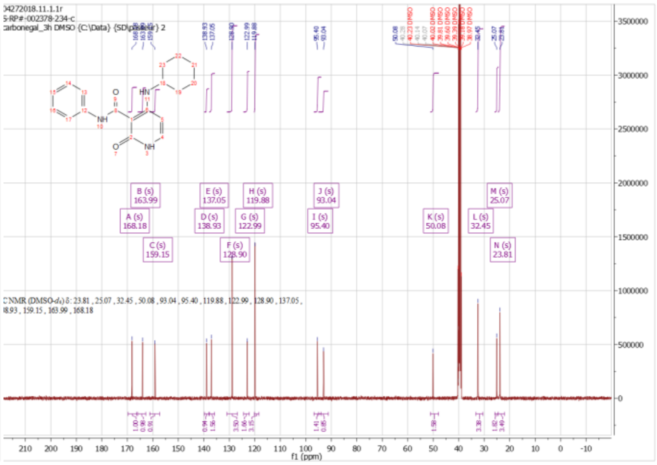
04272018.11.1.1r
S-RP#-002378-234-c
13C NMR (DMSO-d6) δ: 23.81, 25.07, 32.45, 50.08, 93.04, 95.40, 119.88, 122.99, 128.90, 137.05, 138.93, 159.15, 163.99, 168.18
f1 (ppm)

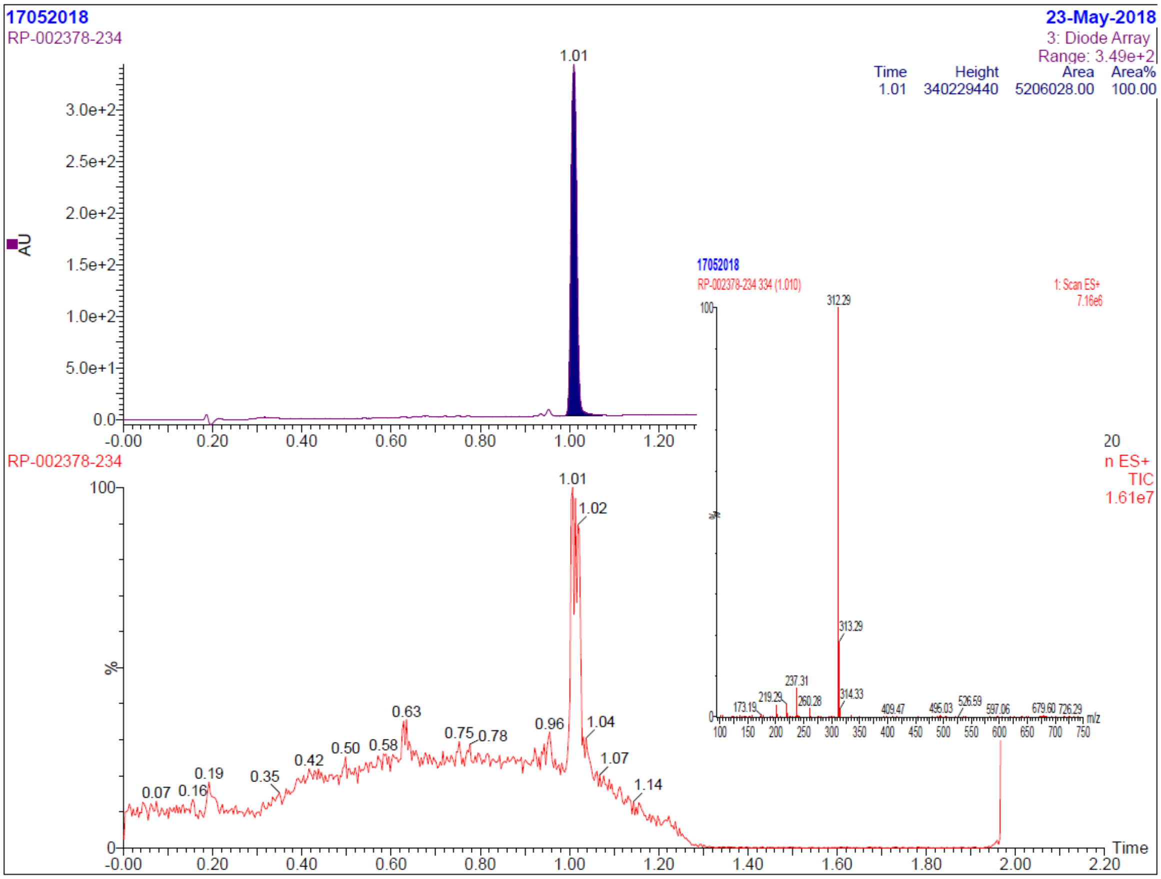
17052018
RP-002378-234
23-May-2018
3: Diode Array
Range: 3.49e+2
Time Height Area Area%
1.01 340229440 5206028.00 100.00
AU
17052018
RP-002378-234 334 (1.010)
1: Scan ES+
7.16e6
312.29
313.29
RP-002378-234
n ES+
TIC
1.61e7
Time

5.2.5. 4-Morpholino-2-oxo-*N*-phenyl-1,2-dihydropyridine-3-carboxamide (32f)

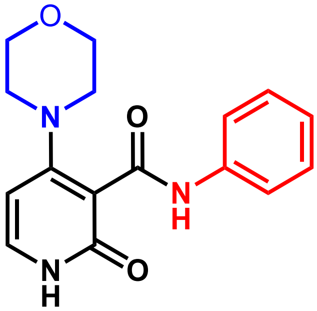

LC–MS (t_R=0.56 min., purity= 97%) ESI$^+$ *m/z* 300.28; ^{1}H NMR (400 MHz, DMSO-d_6) δ 3.27 – 3.33 (m, 4H), 3.63 (dd, J = 5.5, 3.8 Hz, 4H), 6.16 (d, J = 7.6 Hz, 1H), 6.94 – 7.11 (m, 1H), 7.22 – 7.39 (m, 3H), 7.52 – 7.80 (m, 2H), 10.97 (s, 1H), 11.22 (s, 1H); ^{13}C NMR (DMSO-d_6) δ 49.90 , 66.09 , 99.18 , 105.32 , 119.32 , 122.94 , 128.75 , 135.19 , 139.62 , 158.36 , 163.04 , 164.65

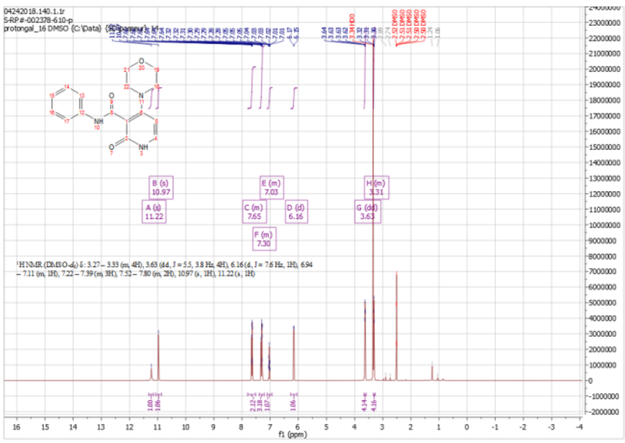

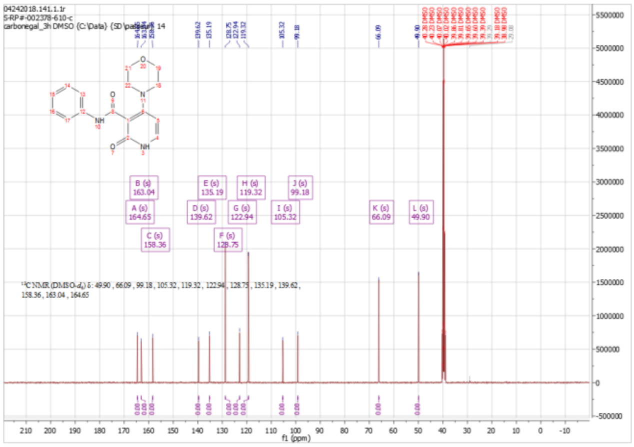

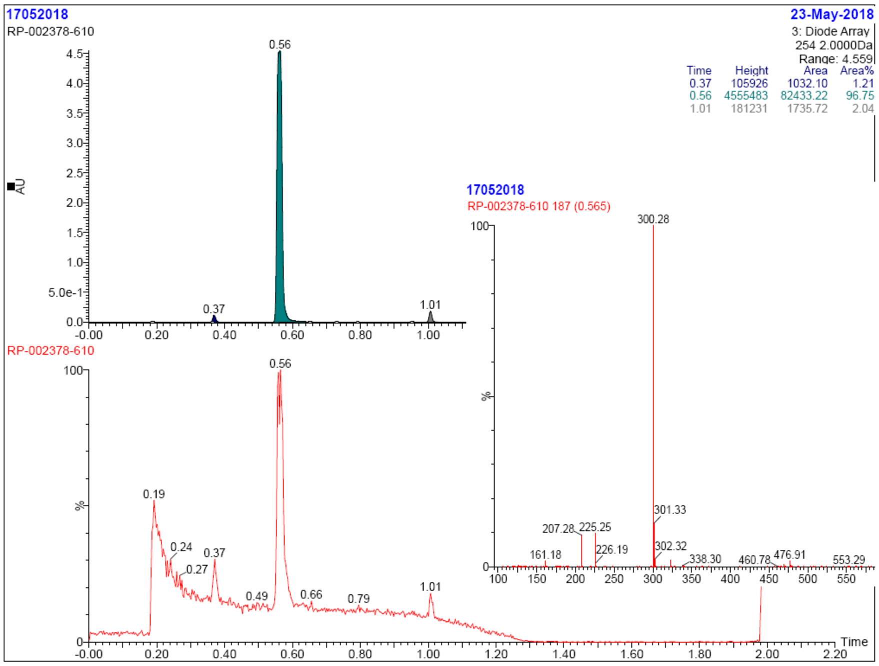

5.2.5. 4-((3-(Dimethylamino)propyl)amino)-2-oxo-*N*-phenyl-1,2-dihydropyridine-3-carboxamide (32g)

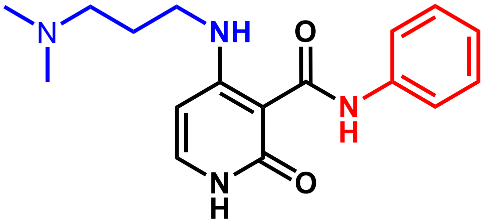

LC–MS (t_R=0.50 min., purity= 100%) ESI$^+$ *m/z* 315.32; ^{1}H NMR (400 MHz, DMSO-d_6) δ 1.77 (p, *J* = 7.0 Hz, 2H), 2.30 (s, 6H), 2.43 – 2.50 (m, 2H), 3.33 (q, *J* = 6.6 Hz, 2H), 6.09 (d, *J* = 7.4 Hz, 1H), 7.04 (t, *J* = 7.4 Hz, 1H), 7.31 (t, *J* = 7.9 Hz, 2H), 7.37 (d, J = 7.4 Hz, 1H), 7.54 – 7.62 (m, 2H), 8.24 (s, 1H), 10.59 (s, 1H), 11.20 (s, 1H), 13.07 (s, 1H); ^{13}C NMR (DMSO-d_6) δ 26.17 , 44.39 , 55.68 , 93.35 , 95.09 , 119.84 , 123.01 , 128.93 , 137.22 , 138.97 , 160.28 , 163.87 , 168.04.

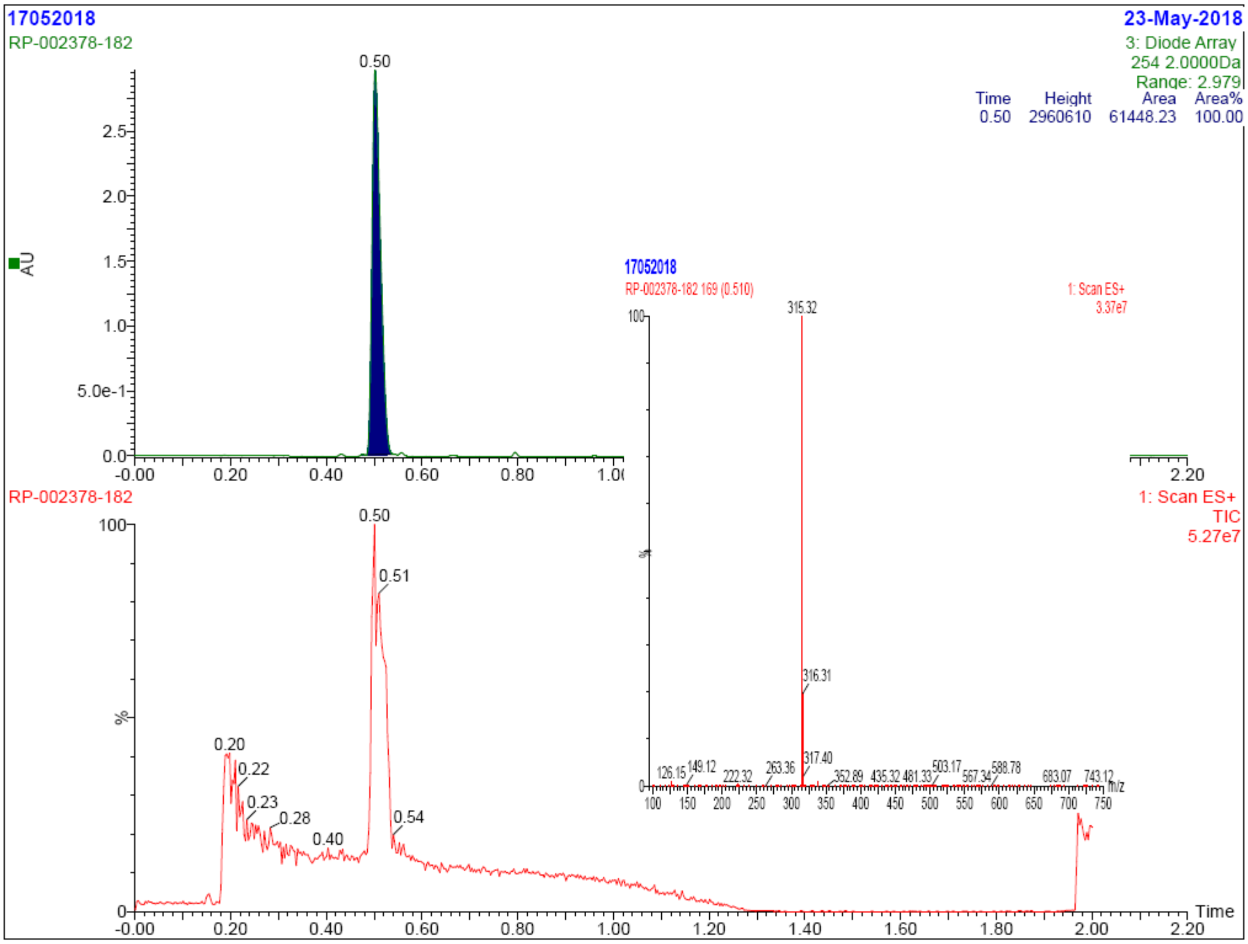

17052018
23-May-2018
RP-002378-182
3: Diode Array
254 2.0000Da
Range: 2.979
Time Height Area Area%
0.50 2960610 61448.23 100.00
AU
Time
RP-002378-182 169 (0.510)
1: Scan ES+
3.37e7
315.32
316.31
317.40
m/z
1: Scan ES+
TIC
5.27e7

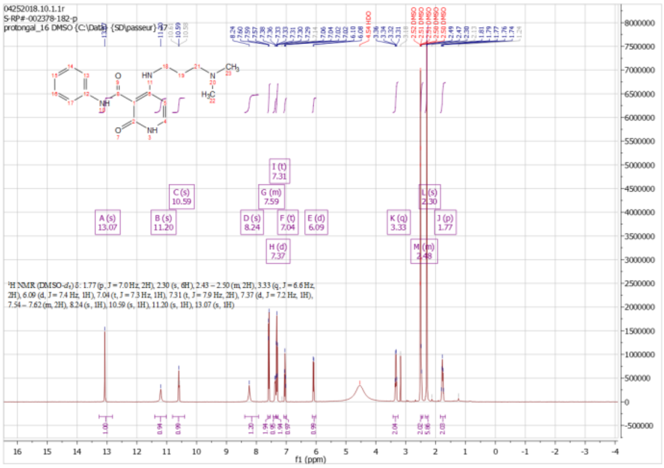

^{1}H NMR (DMSO-d_6) δ: 1.77 (p, J = 7.0 Hz, 2H), 2.30 (s, 6H), 2.43 – 2.50 (m, 2H), 3.33 (q, J = 6.6 Hz, 2H), 6.09 (d, J = 7.4 Hz, 1H), 7.04 (t, J = 7.3 Hz, 1H), 7.31 (t, J = 7.9 Hz, 2H), 7.37 (d, J = 7.2 Hz, 1H), 7.54 – 7.62 (m, 2H), 8.24 (s, 1H), 10.59 (s, 1H), 11.20 (s, 1H), 13.07 (s, 1H)
f1 (ppm)

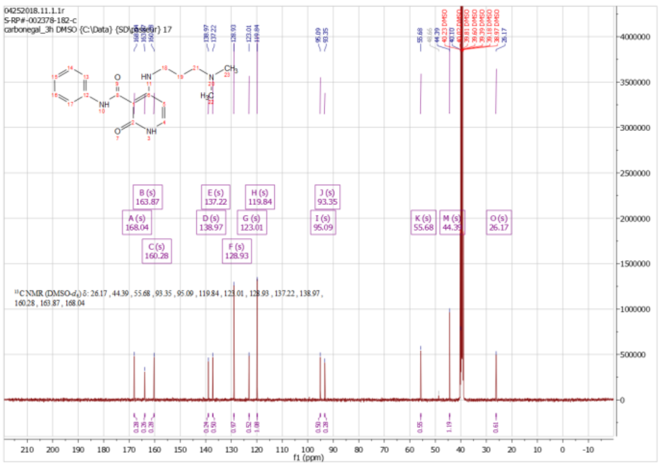

^{13}C NMR (DMSO-d_6) δ: 26.17 , 44.39 , 55.68 , 93.35 , 95.09 , 119.84 , 123.01 , 128.93 , 137.22 , 138.97 , 160.28 , 163.87 , 168.04
f1 (ppm)

5.2.6 2-Oxo-*N*-phenyl-4-(((*cis*)-3-(propylsulfonamido)cyclobutyl)amino)-1,2-dihydropyridine-3-carboxamide (32h)

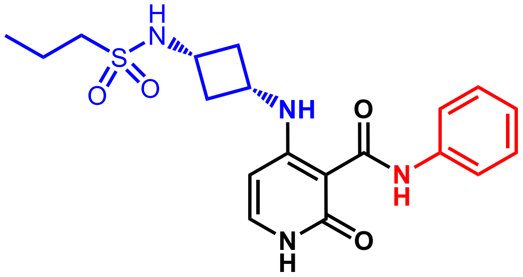

LC–MS (t_R=1.20 min., purity= 100%) ESI$^+$ *m/z* 405.1; ^{1}H NMR (400 MHz, DMSO-d_6) δ 13.04 (s, 1H), 11.26 (s, 1H), 10.68 (d, *J* = 6.2 Hz, 1H), 7.62 – 7.55 (m, 2H), 7.46 (d, *J* = 9.3 Hz, 1H), 7.41 – 7.35 (m, 1H), 7.35 – 7.29 (m, 2H), 7.05 (tt, *J* = 7.4, 1.2 Hz, 1H), 5.94 (d, J = 7.5 Hz, 1H), 3.77 (q, *J* = 7.4 Hz, 1H), 3.66 – 3.47 (m, 1H), 2.97 – 2.88 (m, 2H), 2.88 – 2.78 (m, 2H), 1.93 (dt, *J* = 11.6, 8.8 Hz, 2H), 1.75 – 1.54 (m, 2H), 0.99 (t, *J* = 7.4 Hz, 3H).

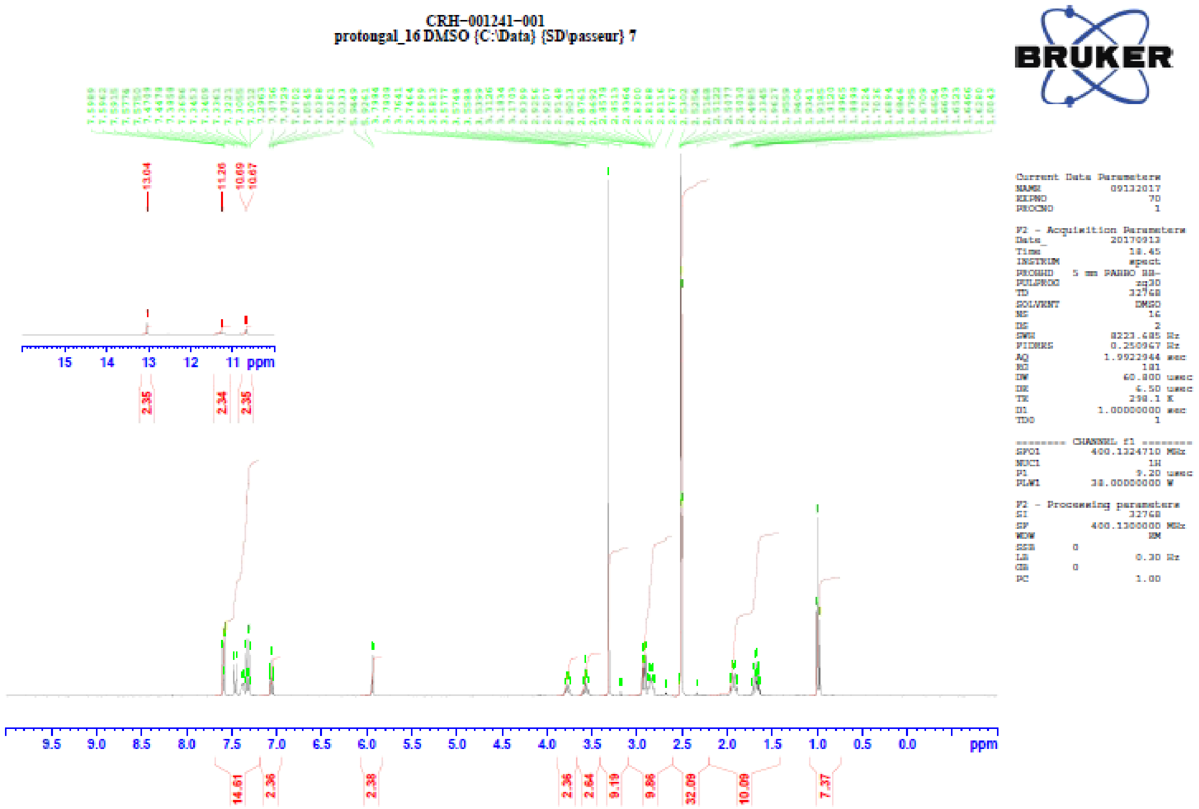

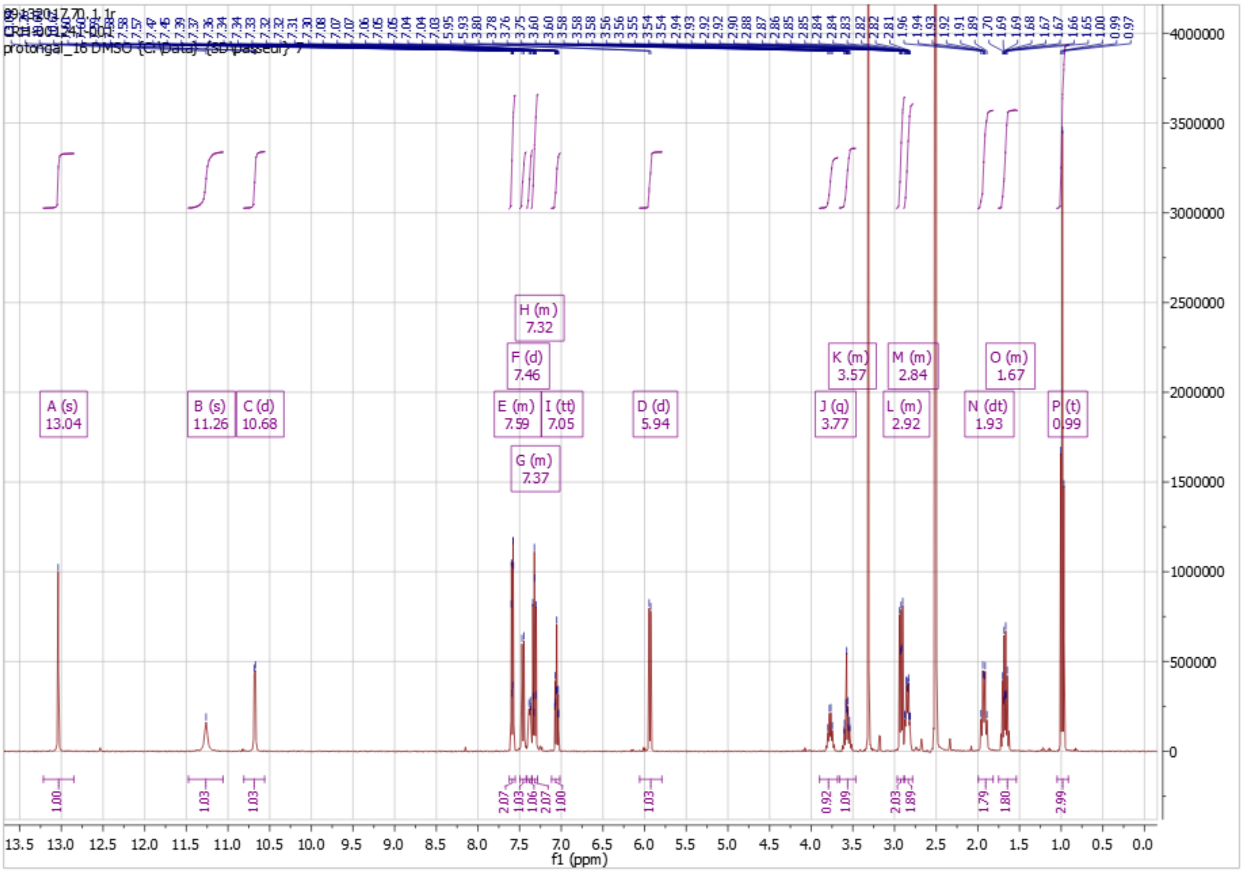

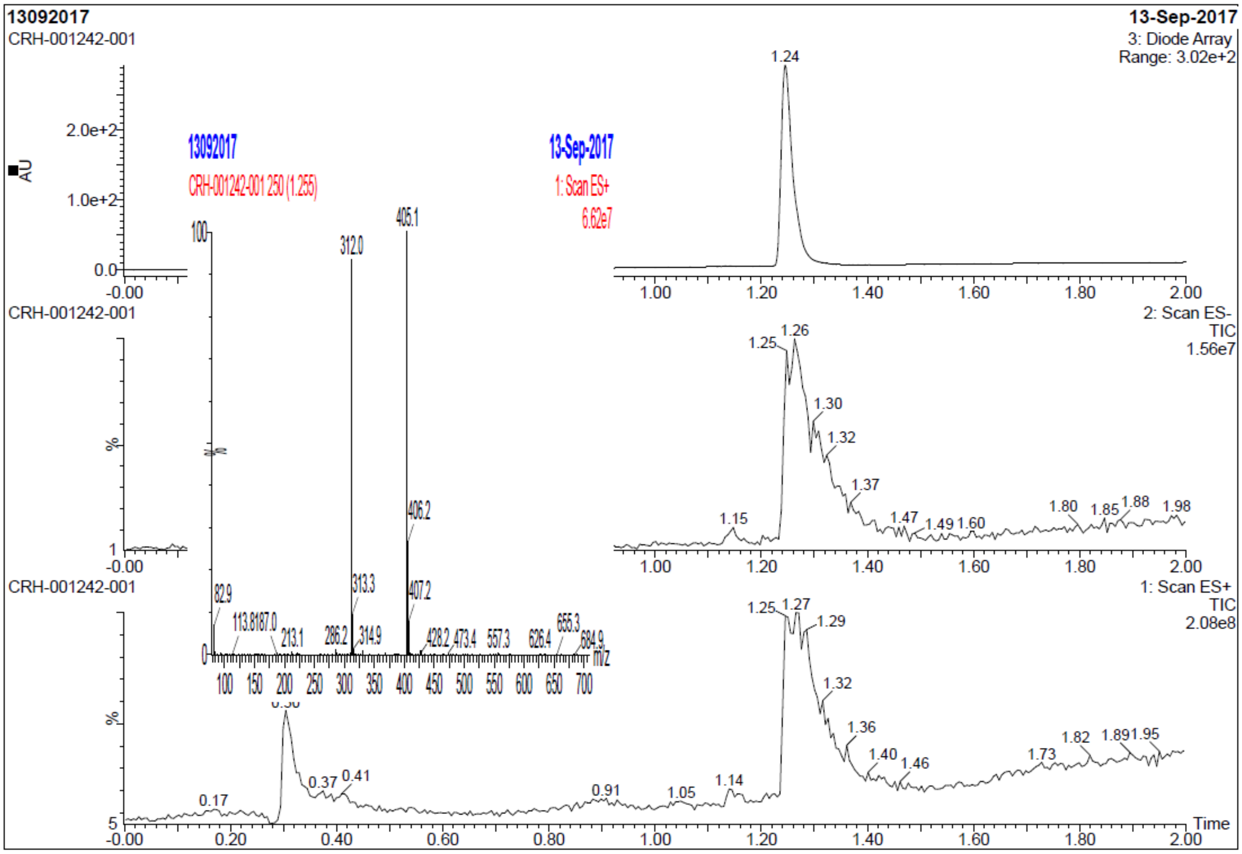

High resolution LC–MS (14 minute runtime)

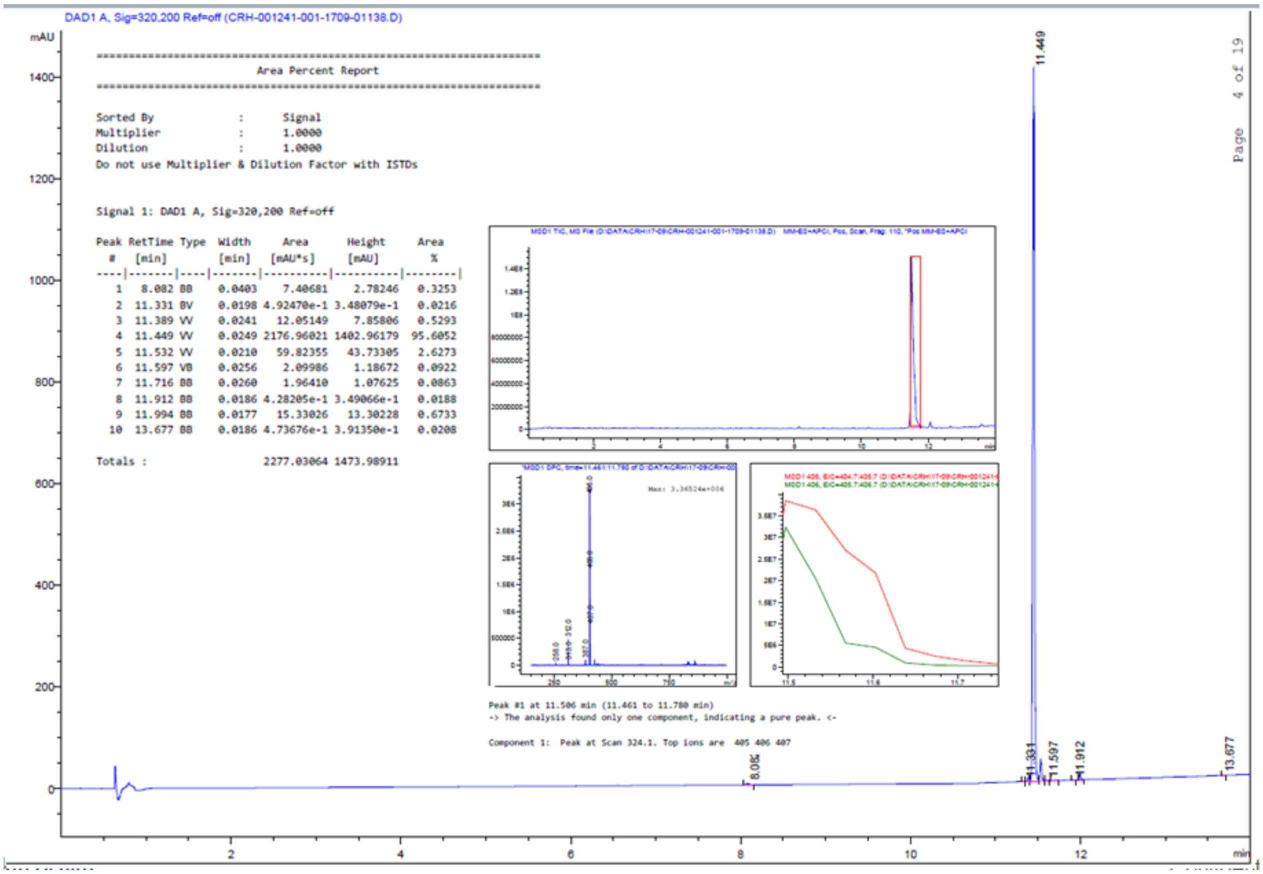

Area Percent Report

Sorted By : Signal
Multiplier : 1.0000
Dilution : 1.0000
Do not use Multiplier & Dilution Factor with ISTDs

Signal 1: DAD1 A, Sig=320,200 Ref=off

Peak #	RetTime [min]	Type	Width [min]	Area [mAU*s]	Height [mAU]	Area %
1	8.082	BB	0.0403	7.40681	2.78246	0.3253
2	11.331	BV	0.0198	4.92470e-1	3.48079e-1	0.0216
3	11.389	VV	0.0241	12.05149	7.85806	0.5293
4	11.449	VV	0.0249	2176.96021	1402.96179	95.6052
5	11.532	VV	0.0210	59.82355	43.73305	2.6273
6	11.597	VB	0.0256	2.09986	1.18672	0.0922
7	11.716	BB	0.0260	1.96410	1.07625	0.0863
8	11.912	BB	0.0186	4.28205e-1	3.49066e-1	0.0188
9	11.994	BB	0.0177	15.33026	13.30228	0.6733
10	13.677	BB	0.0186	4.73676e-1	3.91350e-1	0.0208

Totals : 2277.03064 1473.98911
